# Development and validation of prediction models for stroke and myocardial infarction in type 2 diabetes based on health insurance claims: does machine learning outperform traditional regression approaches?

**DOI:** 10.1186/s12933-025-02640-9

**Published:** 2025-02-18

**Authors:** Anna-Janina Stephan, Michael Hanselmann, Medina Bajramovic, Simon Schosser, Michael Laxy

**Affiliations:** 1 Professorship for Public Health and Prevention, TUM School of Medicine and Health, Technical University of Munich, Munich, Germany; 2 German Center for Diabetes Research (DZD), Munich, Germany; 3 Department of Statistics, Ludwig-Maximilians-Universität München, Munich, Germany

**Keywords:** Machine learning, Health insurance, Claims database analysis, Predictive algorithms, Prediction model, Risk scores, Type 2 diabetes, Myocardial infarction, Stroke, Logistic regression, Deep learning

## Abstract

**Background:**

Digitalization and big health system data open new avenues for targeted prevention and treatment strategies. We aimed to develop and validate prediction models for stroke and myocardial infarction (MI) in patients with type 2 diabetes based on routinely collected high-dimensional health insurance claims and compared predictive performance of traditional regression with state-of-the-art machine learning including deep learning methods.

**Methods:**

We used German health insurance claims from 2014 to 2019 with 287 potentially relevant literature-derived variables to predict 3-year risk of MI and stroke. Following a train-test split approach, we compared the performance of logistic methods with and without forward selection, LASSO-regularization, random forests (RF), gradient boosting (GB), multi-layer-perceptrons (MLP) and feature-tokenizer transformers (FTT). We assessed discrimination (Areas Under the Precision-Recall and Receiver-Operator Curves, AUPRC and AUROC) and calibration.

**Results:**

Among *n* = 371,006 patients with type 2 diabetes (mean age: 67.2 years), 3.5% (*n* = 13,030) had MIs and 3.4% (*n* = 12,701) strokes. AUPRCs were 0.035 (MI) and 0.034 (stroke) for a null model, between 0.082 (MLP) and 0.092 (GB) for MI, and between 0.061 (MLP) and 0.073 (GB) for stoke. AUROCs were 0.5 for null models, between 0.70 (RF, MLP, FTT) and 0.71 (all other models) for MI, and between 0.66 (MLP) and 0.69 (GB) for stroke. All models were well calibrated.

**Conclusions:**

Discrimination performance of claims-based models reached a ceiling at around 0.09 AUPRC and 0.7 AUROC. While for AUROC this performance was comparable to existing epidemiological models incorporating clinical information, comparison of other, potentially more relevant metrics, such as AUPRC, sensitivity and Positive Predictive Value was hampered by lack of reporting in the literature. The fact that machine learning including deep learning methods did not outperform more traditional approaches may suggest that feature richness and complexity were exploited before the choice of algorithm could become critical to maximize performance. Future research might focus on the impact of different feature derivation approaches on performance ceilings. In the absence of other more powerful screening alternatives, applying transparent regression-based models in routine claims, though certainly imperfect, remains a promising scalable low-cost approach for population-based cardiovascular risk prediction and stratification.

**Graphical abstract:**

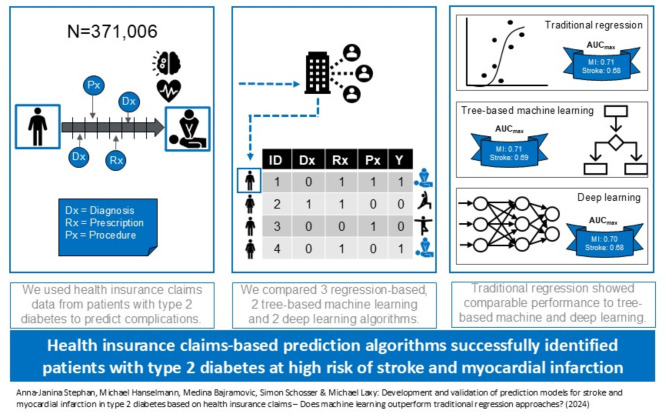

**Supplementary Information:**

The online version contains supplementary material available at 10.1186/s12933-025-02640-9.

## Background

Diabetes complications have a high economic impact on healthcare systems [1, 2]. Especially for macrovascular complications such as stroke and myocardial infarction (MI), prevention is still feasible and effective even within relatively short time distance to the event. Potential secondary prevention measures include pharmaceutical interventions (anticoagulants, statins, angiotensin-converting enzyme inhibitors), and stenting procedures. However, if such interventions are offered to individuals with low cardiovascular risk, they may entail unnecessary psychological burden, physiological risks and deplete the healthcare system of resources needed elsewhere. Targeted screen-and-treat approaches for patients at high complication risk may therefore be more (cost-)effective than universal screening [3]. As a consequence, reliable, simple, and practical prediction models are increasingly used for treatment decisions. For example, the American Diabetes Association recommends use of a validated 10-year risk score for first atherosclerotic cardiovascular events to target new expensive lipid-lowering therapies towards high-risk patients [4]. Similarly, risk scores are already used in practice to guide other therapeutic and clinical decisions, such as anticoagulant prescriptions and intensive care transfers [5]. In Germany, a bill for the usage of healthcare data, the “Gesundheitsdatennutzungsgesetz” was passed in March 2024, and now allows health insurances to use risk scores on their insurees’ claims for targeted screening without prior individual consent. The aim is to establish alert systems that inform and animate insurees to participate in tailored programs for specific case- or disease management [6]. One prerequisite for successful implementation are available, practically applicable, well-calibrated, discriminative models that are internally and externally validated. However, existing models were mostly developed on selected or deeply phenotyped populations and are therefore neither useful nor applicable in real-world contexts with limited available information and broad populations to be screened [7]. In this respect, data routinely collected within the healthcare system, such as electronic health records or data used by health insurances for reimbursement purposes, offer novel, but underexploited opportunities to develop generalizable models that identify diabetes patients at high complication risk. The evolution of artificial intelligence and state-of-the-art machine learning including deep learning (ML/DL) methods hold promise to further improve risk predictions within big health system data such as insurance claims, as these algorithms may outperform traditional regression and classification methods in identifying potentially overlooked predictors and nonlinear relationships [8]. However, this expectation has also been questioned [9]. Our objective was to develop and validate prediction models for stroke and MI in patients with type 2 diabetes based on German Statutory Health Insurance (SHI) claims data that can realistically be applied in routine care settings. Specifically, we wanted to investigate how well prediction models that use only information available in routinely collected real-world secondary data predict diabetes complications. Second, we wanted to investigate if state-of-the-art ML/DL methods outperform traditional regression and classification approaches in this task.

## Methods

Our project “Moving to Next Generation Healthcare (MNGHC)” addressed the objectives stated above across two project parts. In MNGHC-ML we developed the study design and trained and validated models using regression and tree-based machine learning algorithms. In MNGHC-DL, we added models based on two deep learning algorithms. We pre-specified and published model development plans [10, 11] for both project parts before carrying out the analyses, considering potential sources of bias outlined in the Prediction model Risk Of Bias ASsessment Tool (PROBAST) [12]. We aligned conceptual study design decisions with the overall aim to develop prediction models that can be applied in real-world health system contexts for risk stratification. Deviations from the original model development plans with reasons are summarized in Additional files 1–2. Reporting follows the Transparent Reporting of a multivariable prediction model for Individual Prognosis Or Diagnosis (TRIPOD) [13] guidelines (Additional file 3). Note that even though we fully pre-specified all study design aspects before starting data analysis activities, at the time of analysis plan development data collection was already completed (i.e. there is a retrospective aspect to this study). Nevertheless, data collection of health insurance claims occurs strictly prospectively with calendar time, and also the analysis perspective that we took for model development from cohort creation via predictor to outcome measurement was prospective in nature.

### Data sources

In Germany, 85–90% of the population is insured under the SHI scheme offered through > 90 health insurance companies. All SHI companies are legally obliged to cover the same services, charge the same income-dependent contributions, and collect the same data in comparable formats to process their insurees’ claims. We used claims data from one of the largest German SHIs (5.5 million insurees) covering the period from 01/01/2014 to 09/30/2019. Provided datasets covered utilization of outpatient, inpatient, ambulatory inpatient, and rehabilitation services (ICD-10 diagnoses, admission and discharge dates, performed procedure codes (OPS), diagnosis related groups (DRGs), and participation in disease management programs (DMPs)), outpatient prescriptions of medications, devices, aids and remedies, and socio-demographic characteristics from the master beneficiary file [11].

### Study population

We included individuals aged ≥ 18 years with a documented diagnosis for type 2 diabetes following a published [1] algorithm (Additional files 4–5). Patients who deceased were not removed from the dataset, because such competing events to predicted outcomes occur. However, we restricted the study population to individuals < 80 years, as mortality from competing causes in older diabetes patients increases sharply. We did not implement eligibility restrictions with regard to previous MI or stroke events or duration of diabetes (prevalent vs. newly recorded diagnosis). Instead, this information was incorporated as potential predictors (features) in the models. Further details on cohort definition and eligibility criteria are provided in Additional file 4. Eligibility criteria were applied to an observation period from 01/01/2015 to 12/31/2015, on which we trained our prediction models (further explanations below). Only patients who died during this observation period were removed from the dataset for logical reasons.

### Primary outcomes: stroke and myocardial infarction

We chose stroke and MI as target outcomes because they are incident events with a clearly identifiable onset in health insurance claims, potentially preventable even within short lead times, not necessarily foreseeable (i.e. there is a potential for algorithms to support and guide care), and show relatively high per case costs [1]. Outcome identification in the data was based on ICD-10-GM codes (Additional files 6–7) [1].

### Feature engineering

Ex ante, we defined 394 potentially informative predictors (“features”) based on published machine learning projects in claims and clinical data, other existing diabetes-related prediction models and further features available in German claims [11]. This initial list was reduced to 287 features before training, mainly to reduce collinearities and eliminate features with large shares of missing values and little or no observed variation.

The final feature list and reasons for deviations from the originally assembled feature pool are provided in Additional files 9–23. More information on feature engineering is provided in Additional file 24.

### Participant timeline

We defined a one-year observation (Q1-Q4 2015), a six-month buffer (Q1-Q2 2016) and a three-year target period (Q3 2016-Q2 2019). Features were coded for the observation period and used to predict outcomes in the target period. While outcomes occurring in the buffer period were ignored, the respective patients were kept in the dataset such that they were able to contribute potential recurrent instances of these outcomes in the subsequent target period. The buffer period takes into account the time-lagged availability of outpatient claims in Germany (the longest delay among all sectors, which is around six months). Not having the buffer period would imply predicting events after their occurrence in real-world prospective applications, precluding any potential preventative action [12].

The three-year target period was selected as the longest possible (for ease of interpretation) full-year period after defining observation and buffer periods. For a graphical display see Additional file 25.

The observation period was set to 2015 to allow inclusion of a feature that classified the type 2 diabetes condition as either newly recorded (“incident”) or prevalent. Patients who were coded as diabetes-free in 2014 (Additional files 4–5), and subsequently as having type 2 diabetes in 2015 (Additional files 9–10), were defined as newly recorded.

### Training and test sets

We followed an out-of-sample internal model validation approach. Observations were randomly divided into training and test data using a split ratio of 80%:20% [14]. The former were exclusively used for model development but not for performance assessment. Vice versa, test data were exclusively used for performance assessment but not training.

### Software and program codes

Details on the software we used and a data dictionary are available in Additional file 26, which also contains weblinks to the publicly available program codes we developed for data preparation, model training, and performance evaluation.

### Model development

We trained three logistic regression models (a full model using all features, a model with forward selection, and a model employing Least Absolute Shrinkage and Selection Operator (LASSO) regularization), and two models employing tree-based machine learning (random forests (RF), gradient boosting (GB)), and deep learning algorithms (multi-layer perceptron (MLP), feature tokenizer transformer (FTT)), respectively. In addition, we created a null model without any predictors and therefore without any explanatory power for comparison. More information on the different modelling methods is presented in Additional file 27. Technical details on parameter tuning can be found in Additional files 28–29. To ensure comparability of performance metrics, the same set of features was used for training all models without further pre-selection.

### Model validation

Predictive performance was evaluated in terms of *discrimination*, *calibration* and *classification*. *Discrimination* describes the ability to differentiate between individuals who will develop the outcome and those that will not. *Calibration* describes the agreement between predicted probabilities and observed event rates. Once a specific cut-off within the range of a model’s predicted probabilities has been chosen to classify new observations as either predicted cases or predicted non-cases, *classification* performance (i.e. the degree to which individuals are correctly classified) at this specific threshold can be evaluated. Due to the high outcome imbalance (low event incidence), we chose the area under the precision-recall curve (AUPRC) as primary discrimination performance metric [15]. In addition, we report the area under the receiver-operator curve (AUROC) as a widely used discrimination metric.

Models that perform well in terms of discrimination may still considerably under- or overestimate individual risk, with undesirable consequences in practice if treatment decisions are based on the absolute value of predicted risk estimates. So-called mean, weak, and moderate calibration was therefore assessed to understand the accuracy of our models’ predicted probabilities [16].

Classification metrics were plotted against the full range of possible thresholds, and additionally compared using the optimized prediction threshold as identified through the F1 score. We prioritized classification metrics that evaluate performance with regard to the positive minority class (individuals with MI or stroke event), such as sensitivity and positive predictive value.

The Brier score was calculated as a measure of overall performance [17]. Brief definitions of all employed performance metrics can be found in Additional files 30–32. The models were not updated after performance assessment and no risk groups were created.

Relative variable importance was assessed for all logistic and tree-based models. For DL models, variable importance was not estimated. For details see Additional file 27.

## Results

### Study population

Of the *n* = 371,006 individuals with a verified type 2 diabetes diagnosis in the final data set (Additional file 33, 55.3% were female. Mean age was 67.2 years. Average follow-up duration was 2.87 years (1,047.42 days, range: 0–1,094 days). Additional file 34 shows further sample baseline characteristics. Additional file 35 provides a comparison between training and test sets and Additional file 36 reports the number of events per variable.

### Outcomes

During the target period, *n* = 12,701 (3.42%) individuals had at least one stroke and *n* = 13,030 (3.51%) at least one MI record. Also, *n* = 32,237 individuals died during the target period, thereof *n* = 1,593 (*n* = 3,568) during hospitalizations for stroke (MI). Descriptives on the buffer period can be found in Additional file 37.

### Performance results

#### Discrimination

**Fig. 1:**
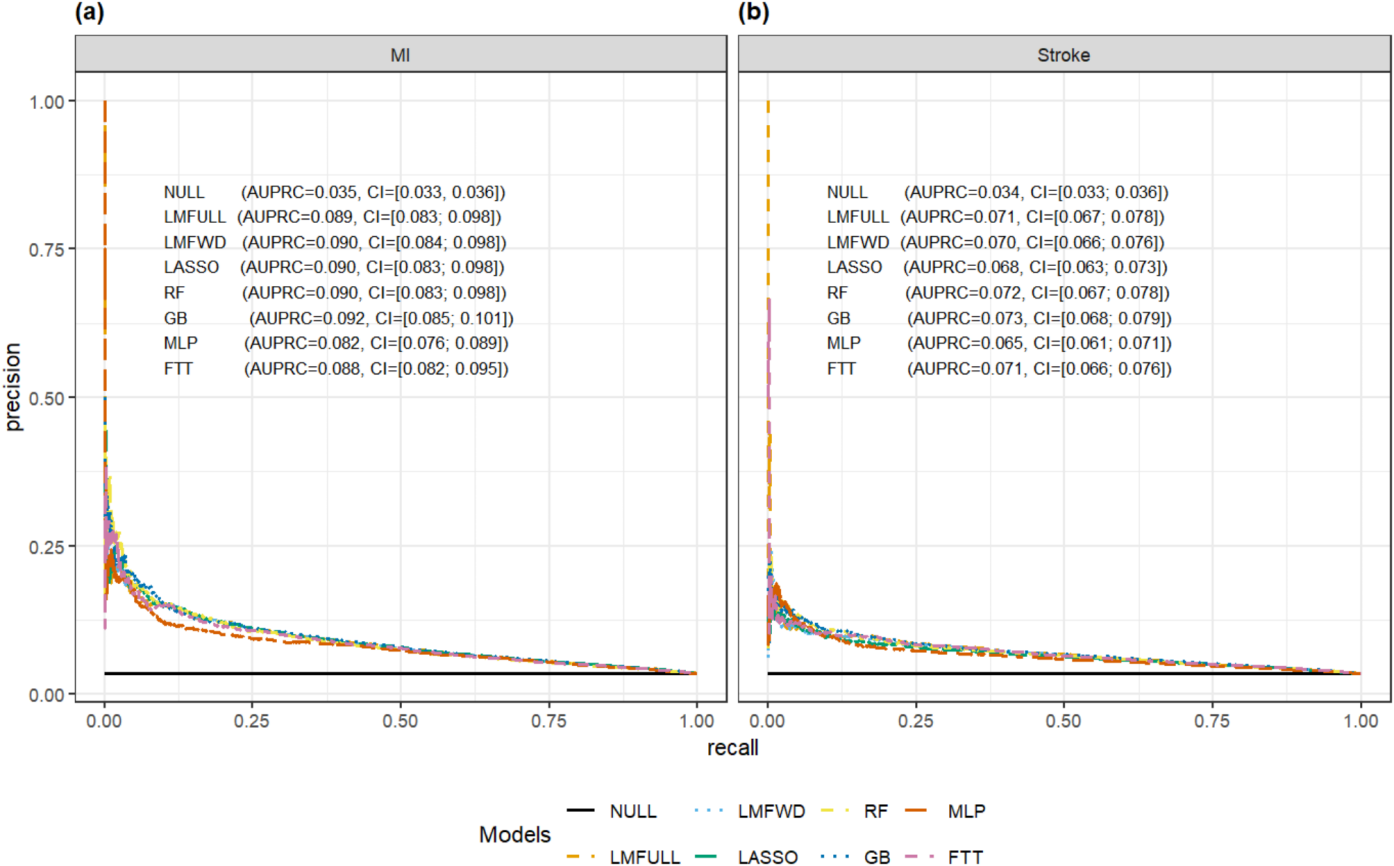
Area under the precision-recall curve for **a** MI (left) and **b** stroke (right) prediction models. *Note:* Both panels represent recall on the x-axis and precision on the y-axis. Model abbreviations and color codes: FTT (pink lines) = Feature-Tokenizer Transformer, GB (dark blue lines) = Gradient Boosting, LASSO (green lines) = Least Absolute Shrinkage and Selection Operator, LMFULL (orange lines) = Full Logistic Model with all features, LMFWD (light blue lines) = Logistic Model with Forward Selection, MLP (red lines) = Multi-Layer Perceptron, NULL (black lines) = Null model without predictors, RF (yellow lines) = Random Forest.

AUPRCs (Fig. 1, Additional file 38) increased from 0.035 for the null model without explanatory power to between 0.082 (MLP) and 0.092 (GB) for MI. For stroke, AUPRCs increased from 0.034 for the null model to between 0.065 (MLP) and 0.073 (GB).

**Fig. 2:**
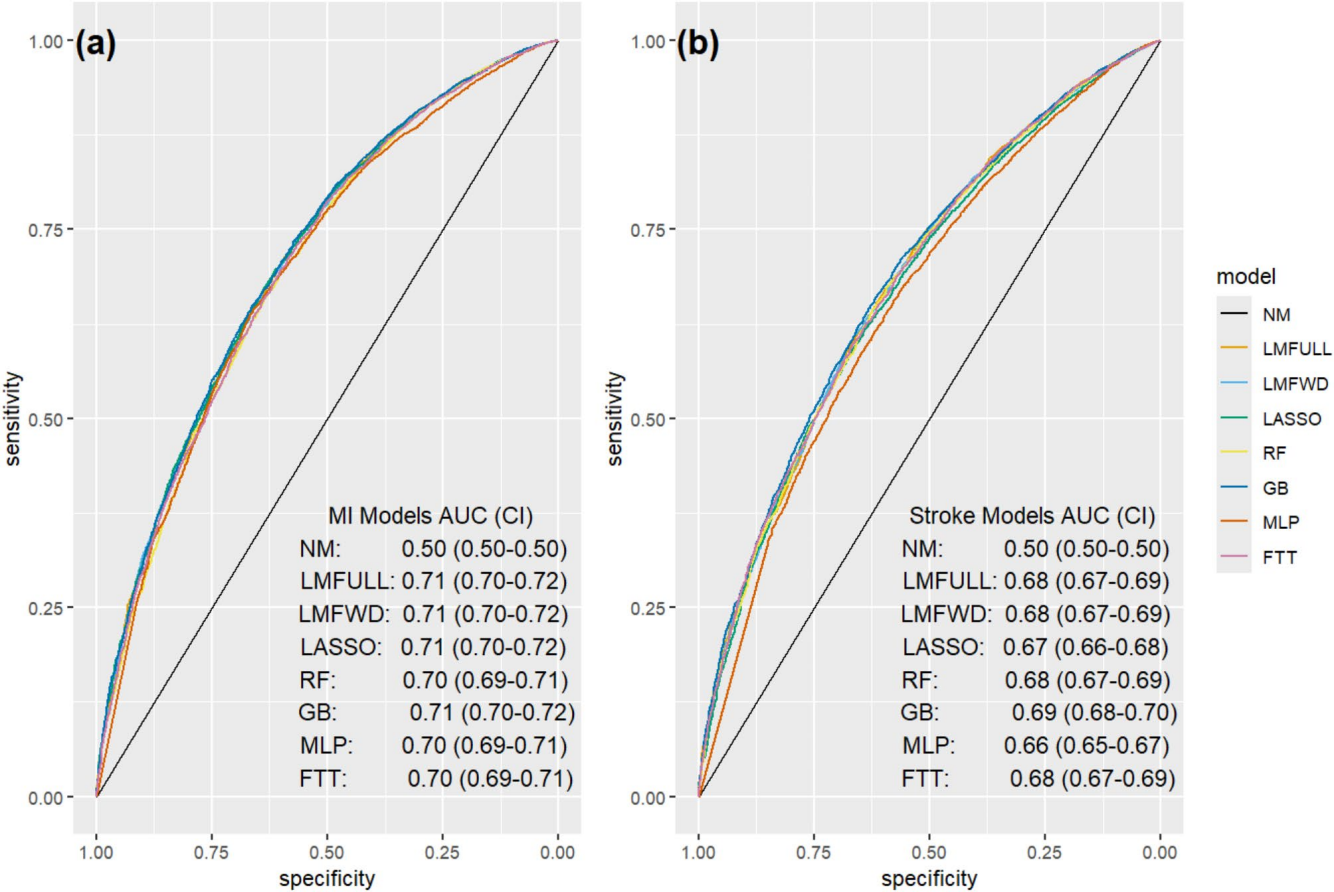
Area under the receiver-operator curve for **a** MI (left) and **b** stroke (right) prediction models. *Note:* Both panels represent specificity on the x-axis and sensitivity on the y-axis. Model abbreviations and color codes: FTT (pink lines) = Feature-Tokenizer Transformer, GB (dark blue lines) = Gradient Boosting, LASSO (green lines) = Least Absolute Shrinkage and Selection Operator, LMFULL (orange lines) = Full Logistic Model with all features, LMFWD (light blue lines) = Logistic Model with Forward Selection, MLP (red lines) = Multi-Layer Perceptron, NULL (black lines) = Null model without predictors, RF (yellow lines) = Random Forest.

AUROCs increased from 0.5 for the null model without explanatory power to between 0.70 (RF, MLP, FTT) and 0.71 (all other methods) for MI and between 0.66 (MLP) and 0.69 (GB) for stroke (Fig. 2, Additional file 39).

#### Calibration

**Fig. 3:**
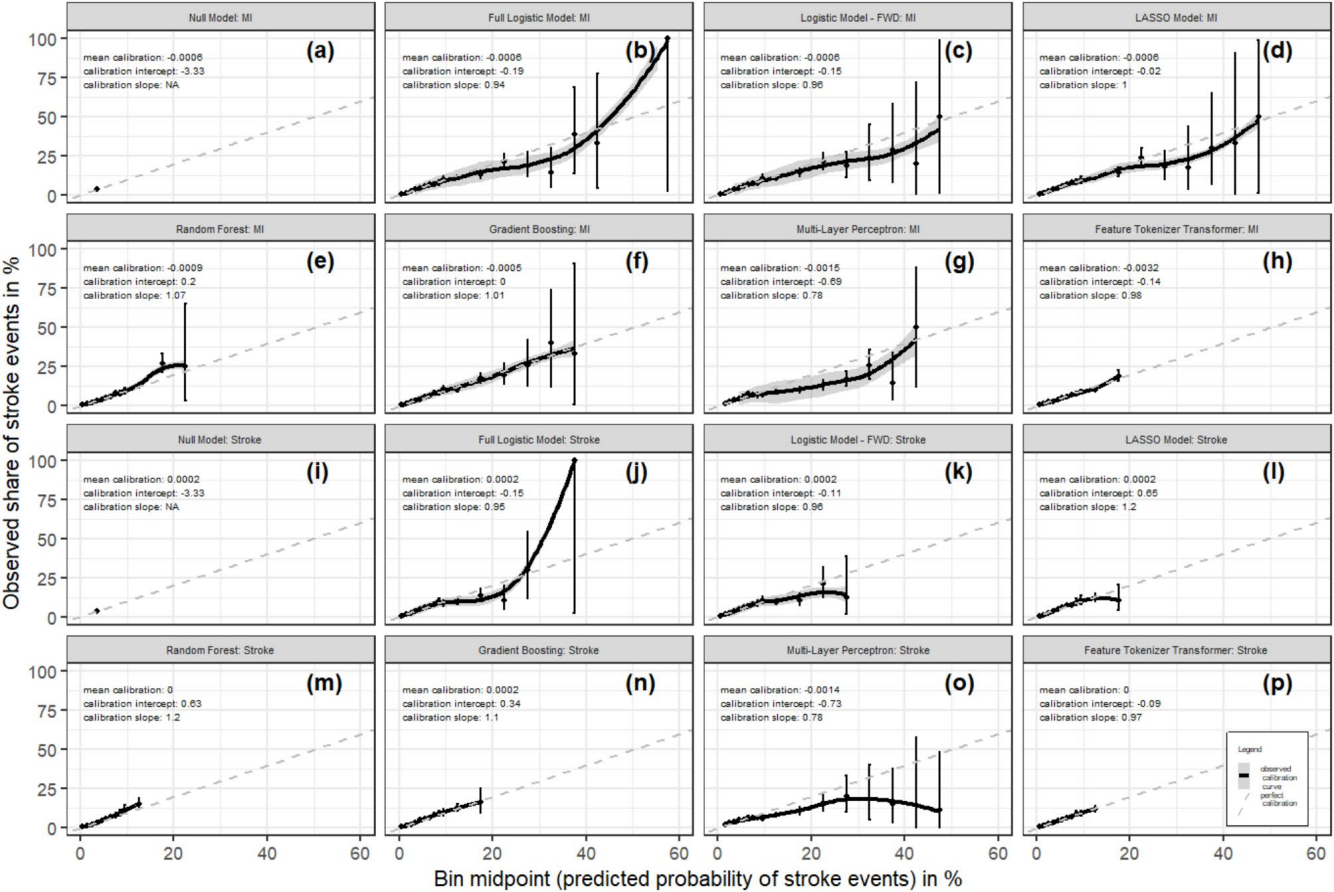
Mean, weak and moderate calibration performance of prediction models for MI **a**–**h** and stroke** i**–**p**. *Note* Mean calibration represents the absolute difference between average predicted risk and overall event rate. Weak calibration represents the calibration slope (spread of the estimated risks), with a target value of 1 and the calibration intercept with a target value of 0. Moderate calibration represents the relationship between predicted values plotted against mean observed values in bins of width 0.01 between 0 and 0.1 and of width 0.05 thereafter. Error bars represent confidence intervals for point estimates in each bin. Predicted probabilities for the Multi-Layer-Perceptrons and Feature-Tokenizer Transformers were additionally scaled with histogram binning.

For MI (Fig. 3), visual assessment of moderate calibration suggested the narrowest range of risk predictions for the FTT and the highest range for the full logistic model, with a tendency to overestimate high risks in all models except RF and GB which slightly underestimated higher risks, and FTT (neither risk over- nor underestimation of higher risks), with the latter three calibration curves deviating least from the diagonal.

For stroke (Fig. 3), visual assessment of moderate calibration suggested the narrowest range of risk predictions for RF and FTT and the highest for the full logistic model and MLP, with a tendency to overestimate high risks in the full and forward selected logistic models and the MLP, and little to no deviations from the diagonal for LASSO, RF, GB and FTT.

#### Classification

Sensitivity at the exemplary cut-off selected based on the maximized F1 score was highest for the FTT MI (0.342) and the full logistic stroke model (0.345), while PPV at this cut-off was highest for the forward selected MI (0.119) and the GB stroke model (0.094).

Density plots of predicted probabilities for cases and non-cases are shown in Additional files 39–40, plots of classification performance in Additional file 41–42. For additional results on classification performance at F1 score optimized thresholds and overall model performance see Additional files 43–44.

#### Variable importance

**Fig. 4:**
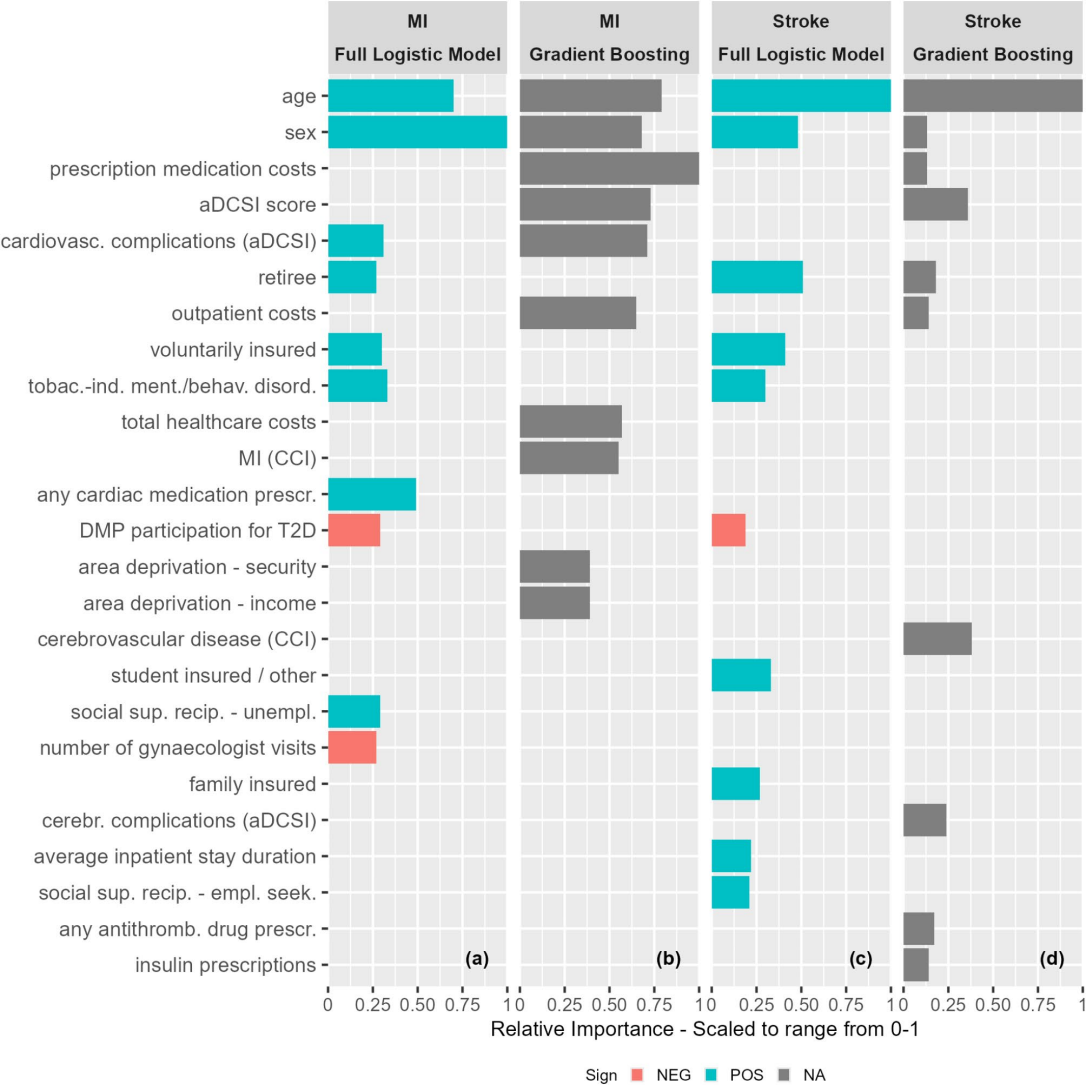
Ten most important variables for full logistic and gradient boosting MI and stroke models. *Note*: For each panel, the x-axis represents the relative variable importance of the variables depicted on the y-axis in the respective model. The y-axis lists all variables that are among the ten most important variables in at least one of the four depicted models. Variable importance is scaled to range between 0 (not important) and 1 (highest importance). Bar lengths indicate relative variable importance, but do not convey information on absolute importance. Variables without bars may have scaled importance values > 0 for a model, but the respective values are not shown if a variable does not fall under the respective model’s ten most important variables. Color codes for bars in the logistic regression-based models indicate the direction of the respective variable’s coefficient sign (negative coefficient indicating lower risk: red; positive coefficient indicating increased risk: blue). Random Forests and Gradient Boosting have grey instead of red or blue bars as they do not provide regression coefficients which could be positive or negative (not applicable). aDSCI = adapted Diabetes Complication Severity Index, cardiovasc. = cardiovascular, CCI = Charlson Comorbidity Index, cerebr. = cerebrovascular, empl. seek. = employment seeking, MI = myocardial infarction, NA = not applicable, NEG = negative, POS = positive, prescr. = prescription, social sup. recip. = social support recipient, T2D = diabetes type 2, tobac.-ind. ment./behav. disord. = mental and behavioral disorders due to use of tobacco, unempl. = unemployed.

The 10 most important variables according to the full logistic (our “state-of-the-art reference case”) and GB (our overall “best performing approach”) models are depicted in Fig. 4.

While some overlap in important variables existed across methods and outcomes, other variables resulted as important only for single methods or outcomes. For MI, both algorithms identified age, sex, previous cardiovascular complications, and either a previous MI, ischemic heart disease, or cardiac medication as important features.

For stroke, both algorithms identified age, sex, and being retired as important.

Additional files 45–46 include variable importance plots of the 10 most important variables and Additional files 47–48 list the 30 model-specific most important variables for all logistic and tree-based models. Previous events and prescribed antihypertensive, antithrombotic, and antidiabetic agents as well as gynaecologist visits were important in multiple models for both indications. Neurological disorders (including dementia) were important for several stroke, mental disorders due to use of alcohol or tobacco, and paralysis for several MI models. Generally, continuous features such as area deprivation, healthcare contact frequencies and costs showed higher importance for tree-based models, and categorical non-dichotomous features (insurance status, area type) showed higher importance for logistic regression-based models.

## Discussion

Using German SHI data, we showed that health insurance claims, which are in many countries the largest source of routinely collected real-world secondary healthcare data, can be used to develop and validate prediction models for stroke and MI in patients with type 2 diabetes. Notably, using a broad set of literature-derived features, we did not find noteworthy performance differences across a broad range of logistic, tree-based and deep learning methods, suggesting that when based on these types of features alone, simpler algorithms may be the preferred choice for the purpose of population-wide screening.

All models were well calibrated and clearly outperformed the respective null model in terms of discrimination, more than doubling AUPRCs and yielding AUROCs of 0.70–0.71 (MI) and 0.66–0.69 (stroke). This is within the AUROC range of other published and validated models of 0.68 [18], 0.69 [19], 0.76 [20], and 0.78 [20] for MI and between 0.64 [21] and 0.88 [22] for stroke [19–28]. Published models used various data sources (cohort data, combinations of claims and lab results) and methods. Previous claims-based models from South Korea [18, 24] and Taiwan [26] included anthropometric and laboratory parameters, information types unavailable in German and many other health systems’ claims. These models yielded AUROCs of 0.70 [24] and 0.72 [26] for 5- and 3-year stroke risk and 0.68 for 5-year MI risk [18]. Two had limited age ranges (40–64 years) and excluded > 20% of observations due to missing values. Notably, despite our data type restrictions and broader population, our models reached comparable AUROC performance. This is in line with one previous study which developed prediction models for major adverse cardiovascular events in type 2 diabetes solely based on U.S. private health insurance claims without anthropometric and laboratory information, with comparable AUROCs of 0.70–0.72 [29]. We are aware of one study [30] that validated the UKPDS-OM2 10-year risk equations for stroke (AUROC = 0.57) and MI (AUROC = 0.58) for Germany using epidemiological cohort data. The considerably longer target window and external validation potentially explain their poor performance.

Previous target windows ranged from 3 [26] to 10 [19] years. As prediction gets more difficult with increasing lead time (visible e.g. in Li et al. 2018, where the 3-year AUROC for stroke of 0.72 decreased to an 8-year AUROC of 0.67) [26], our models might actually perform slightly worse than some predecessors due to unavailability of laboratory or anthropometric information. Also, most validated models use time-to-event analyses combined with simpler regression-based methods as opposed to our simpler classification task with more complex ML/DL methods. We came across one unvalidated MI model [31], but no other internally or externally validated models for stroke or MI in diabetes patients that used more complex ML approaches, even though some efforts seem to be ongoing [32].

Most published models included smaller numbers of features (4 [27] to 24 [28]). However, data hungriness may be a minor practical issue for models which exclusively use readily available data.

It should be noted that the informative value of AUROCs is limited in situations with high outcome class imbalance, as high AUROCs may be driven by high specificity (i.e. good predictions of the negative majority class even in the absence of good predictions of the positive minority class). However, comparison of our models to the literature in terms of better suited performance metrics like AUPRC, PPV and sensitivity was hindered by the fact that previously published models rarely reported these metrics. One exception is the 5-year stroke risk models developed by Yang et al. 2007 [27], who reported a PPV of 0.116 and a sensitivity of 0.657 at their suggested performance threshold. At the optimized F1 value, our stroke model with the highest PPV of 0.094 (GB) reached a sensitivity of 0.212, while the stroke model with the highest sensitivity of 0.345 reached a PPV of 0.08. While cut-offs for our models could be chosen to reach higher PPVs, sensitivity at such cut-offs would be considerably lower and vice versa. Again, the model by Yang et al. incorporated laboratory information unavailable at population level in most health systems’ claims, including Germany and the U.S., and for most other published stroke and MI models, classification performance for the positive minority class remains unclear due to lack of reporting.

Apart from the fact that there are currently no low-cost alternatives with clearly superior performance on relevant metrics for population-based screening, the question remains if the performance of our models is sufficient to justify real-world application. This depends largely on the costs of implementation and consequences of misclassification, which may both be lower for high-level population risk screening than in many clinical settings which directly entail decisions on the use of costly and potentially risky mitigation strategies to avoid event occurrence. In a population-based screening setting, model signals might simply trigger a subsequent contact with the health care system to take more detailed anthropometric and laboratory measurements, which might then in turn allow for more targeted application of prediction models with higher positive predictive values at the cost of more granular data requirements.

All our modelling approaches yielded similar performance results, with Gradient Boosting only marginally outperforming other methods. Even though regularization, tree-based and deep-learning methods make fewer assumptions and adapt more flexibly to the data, in our application they did not seem to “tease out” more information than simpler logistic regression. Potentially SHI data do not contain complex predictive patterns from which adaptive methods could derive increased performance. Alternatively, the literature-based derivation of features, although yielding a well-defined and explainable basis for model development, may have reduced the richness and complexity of information available in the data. This further substantiates that complex ML/DL methods may be advantageous in some, but overhyped in other (data) contexts, highlighting the need to differentiate between preconditions that enable and impede such algorithms to outperform traditional regression [33]. For example, tree-based and deep learning approaches have previously been found to improve claims-based predictions for opioid overdose [34], but not for hospitalization following emergency department visits [35]. For diabetes onset, comparative studies using various data types show equivocal results: In some cases, logistic regression was equally good or better than, only marginally outperformed, or clearly outperformed by more complex methods, or only started outperforming simple logistic regression under specific conditions [36].

Regarding stroke and MI prediction in patients with type 2 diabetes, when using an extensive set of pre-selected literature-derived features, based on our results, we would recommend to either use the GB algorithm for potential future external validation efforts, or even stick to full or forward selection logistic methods for their higher simplicity and explainability and lower computational requirements. However, future projects might want to systematically compare the explanatory power of different sets of features derived from broader and more agnostic approaches than mere literature-based feature identification. For additional technical remarks, see Additional file 49.

All our models identified established risk factors such as age, sex, previous events, and medications indicating presence of specific risks or higher underlying type 2 diabetes severity. Variables such as gynecologist visits, which showed high (protective) importance in some models illustrate that importance does not equal causality [37]: Most probably, gynecologist visits themselves do not affect stroke or MI risk, but carry other relevant information such as identifying as female and higher health literacy (many gynecologist visits are conducted for preventative purposes).

Strengths and limitations of our modelling decisions should be considered when interpreting our results. These concern the (1) underlying data and eligibility criteria, (2) feature identification strategy, (3) feature engineering steps, (4) data splits, (5) modeling methods and (6) validation strategy.

Misclassification errors (for example regarding the differentiation between incident and prevalent diabetes) are possible, as diagnosis records reflect only the formally diagnosed subset of those affected by a disease, and records of diagnoses depend on additional preconditions such as symptoms and resulting contact with the health care system. Conditions which affect physician reimbursement may be over-, and less reimbursement-relevant conditions undercoded. Presence of such misclassification in the training data may have reduced the predictive ability of our models. Most importantly, causes of death are not captured if individuals died without healthcare system contact, thus entailing outcome misclassification of those deaths that were caused by acute MIs and strokes but cannot be identified as such. For stroke, death outside the healthcare system occurs in < 10% of cases [38]. Prehospital MI deaths amount to ~ 24% [39]. Our outcome classification thus represents a selection of healthier individuals (either survived the event or died in care).

Loss to follow-up due to death for other reasons than identifiable (i.e. inpatient) cause-specific (stroke or MI) deaths during the three-year target period was ignored (i.e. treated as “no MI event” / “no stroke event”, respectively), carrying the potential for bias due to competing risks for those individuals who died for reasons other than stroke or MI.

As we limited eligibility to adults < 80 years, extrapolation of predictions to older age remains questionable. Some previously published models included adults above this age cut-off [21–23, 26, 27], while others did not [19, 20, 24–26].

Claims data are prospectively collected with low risk of recall bias but higher risk of incorporation bias (recorded diagnoses may be affected by the physician’s knowledge of patient history) [12]. However, this risk is irrelevant for features and rather low for relatively “hard” endpoints such as MI or stroke.

Our literature-driven feature identification may have limited the potential for new feature discovery to improve prediction. An agnostic strategy could have attempted to create an exhaustive feature list from the available data. However, this would have entailed new challenges (increased multicollinearity, computational resource requirements, suboptimal events-per-variable ratios) and implicit decisions (e.g. dummy coding vs. count variables, derivation of chronological patient pathways, aggregation level for hierarchically structured code systems).

We decided to reduce data cleaning to a minimum, mirroring real-world application. Not imputing missing values limits our models to the > 98% of the target population with complete information.

There were trade-offs between possible lengths of observation, buffer and target periods. A longer observation period may have increased feature informativeness and thereby model performance. From an applied clinical perspective, longer target periods are desirable. Most validated models for stroke and MI use 5-year target windows [18, 20–24, 26, 27], which was impossible with our total of 5.75 available years. Future projects aiming at longer-term claims-based predictions might require time-to-event instead of dichotomous outcomes due increased censoring and competing events [12].

The ideal test set would have been out-of-time or drawn from an entirely different population [12], as mere out-of-sample internal validation can bias performance results upwards. Further external validation in independent samples (e.g. another insurance company or a later time period) is therefore desirable. External validation in claims from other healthcare systems would additionally require an assessment of data comparability in terms of content, structure, and underlying data generating processes (e.g. physician coding behaviors). Lastly, it should be noted that treatment innovations (e.g. newer GLP1 receptor agonists such as semaglutide or tirzepatide for type 2 diabetes) may affect a model’s predictive power over time. Therefore, the models presented here (as, in fact, any prediction model intended for real-world use) will need periodical re-validations to ensure their sustained performance, and updates of the underlying feature sets should be considered as needed.

After external validation, acceptance and (cost-)effectiveness of real-world model application should be evaluated. Meanwhile, legislatory developments are paving the ground for such real-world application. Ideally, this should combine risk prediction with targeted preventative interventions. These might comprise messages to high-risk insurees suggesting to seek care or participate in mitigation measures such as case or disease management programs, and physician alerts to check for derailed risk factors. It will be important to ensure both availability of such interventions and willingness of payers and providers to implement them.

## Conclusions

Using data from Germany, we demonstrate that, despite the fact that secondary big health system data such as insurance claims are inherently noisy due to real-world recording practices, claims-based prediction models for stroke and MI in patients with type 2 diabetes may be used for population-based screening purposes to identify a share of those individuals at higher risk of stroke or MI. Notably, complex machine learning including deep learning methods did not outperform conventional regression approaches, suggesting that either the richness of claims or of the specific literature-based features derived from these data posed a ceiling to model performance. Consequently, when using literature-based features, simpler algorithms may be preferable for population wide screening. In addition, different approaches to feature derivation in these data should be further investigated in the future. Before such models can be implemented in practice, external validation is needed, for which we recommend using either the Gradient Boosting algorithm, or even a logistic method due to their higher simplicity and explainability and lower computational requirements.

## Electronic supplementary material

Below is the link to the electronic supplementary material.

**Additional File 1**: Additional File 1. Summary of deviations from the model development plan. Additional File 2. Deviations in the set of features during actual implementation compared to the pre-specified model development plan. Additional File 3. TRIPOD Checklist. Additional File 4. Definition of the study population. Additional File 5. Algorithm for the identification of type 2 diabetes patients. Additional File 6. Description of the outcome selection process. Additional File 7. Selection algorithm for identification of outcomes following [2]: Stroke and myocardial infarction. Additional File 8. Identification of targeted macrovascular complications based on ICD-10-GM codes. Additional File 9. Definition of newly recorded diabetes. Additional File 10. Additional criteria (applied to the run-in period Q1-Q4 2014) to identify prevalent diabetes diagnoses in the observation period. Additional File 11. Potential socio-demographic and socio-economic predictors based on previous models. Additional File 12. Potential predictors related health care resource utilization and costs based on previous models. Additional File 13. Identification of other diabetes-related complications and events based on ICD-10-GM, OPS- and EBM-codes. Additional File 14. Potential predictors related to multimorbidity or constituting dimensions. Additional File 15. Operationalisation of the individual dimensions of the aDSCI score. Additional File 16. Potential comorbidity related predictors based on previous models. Additional File 17. Potential medication related predictors based on previous models: Antidiabetic drugs. Additional File 18. Potential medication related predictors based on previous models: Antithrombotic drugs (Anticoagulants). Additional File 19. Potential medication related predictors based on previous models: Anti-hypertensive drugs. Additional File 20. Potential medication related predictors based on previous models: Lipid modifying agents. Additional File 21. Potential medication related predictors based on previous models: Cardiac Medications. Additional File 22. Potential medication related predictors based on previous models: CNS-related drugs. Additional File 23. Potential medication related predictors based on previous models: Other drugs. Additional File 24. Feature engineering– technical details. Additional File 25. Time windows used for prediction modeling. Additional File 26. Software and program codes. Additional File 27. Model development– Technical details. Additional File 28. Details on parameter tuning. Additional File 29. Initial ranges and final values for tuning parameters. Additional File 30. Overview and definition of discrimination metrics. Additional File 31. Overview and definition of calibration metrics. Additional File 32. Overview and definition of classification metrics. Additional File 33. Participant flow chart. Additional File 34. Sample characteristics. Additional File 35. Training and test set characteristics. Additional File 36. Events per variable. Additional File 37. Descriptives on the buffer period. Additional File 38. Results on discrimination performance. Additional File 39. Density plot of predicted MI probability for MI cases and non-cases. Additional File 40. Density plot of predicted Stroke probability for Stroke cases and non-cases. Additional File 41. Plot of classification metrics for MI against threshold. Additional File 42. Plot of classification metrics for Stroke against threshold. Additional File 43. Results on classification performance at the respective optimized classification threshold. Additional File 44. Brier score. Additional File 45. Variable Importance for MI models (10 most important variables for each model). Additional File 46. Variable Importance for stroke models (10 most important variables for each model). Additional File 47. Most important variables for MI prediction models. Additional File 48. Most important variables for stroke prediction models. Additional File 49. Discussion of additional technical aspects.

## Data Availability

The two pre-specified model development plans (published May 17, 2022 and November 15, 2022), a data dictionary defining the variables in the dataset as well as the programs used for data management, model development, and model evaluation are publicly available at the OpenScienceFramework (OSF) under the following link: https://osf.io/v2h7d. Analyses of SHI claims data in Germany are allowed only under strict data protection requirements. Therefore, data used for this analysis cannot be made publicly available by the authors nor shared upon individual request to authors. To access the data, approval by the regulatory authority, the German Federal Office for Social Security, must be obtained from researchers through the health insurance.

## Abbreviations

AUPRC: Area under the precision-recall curve
AUROC: Area under the receiver-operator curve
DL: Deep learning
DMP: Disease management program
DRG: Diagnosis related group
FTT: Feature-Tokenizer transformer
GB: Gradient boosting
ICD-10-GM: International classification of diseases, 10th version, german modification
LASSO: Least absolute shrinkage and selection operator
MI: Myocardial infarction
ML: Machine learning
MLP: Multi-layer perceptron
MNGHC: Moving to next generation healthcare
OSF: Open science framework
PROBAST: Prediction model risk of bias assessment tool
OPS: Operation and procedure codes
RF: Random forest
SHI: Statutory health insurance
T2D: Type 2 diabetes
TRIPOD: Transparent reporting of a multivariable prediction model for individual prognosis or diagnosis
