# Supplementary material for: Development and validation of prediction models for stroke and myocardial infarction in type 2 diabetes based on health insurance claims: does machine learning outperform traditional regression approaches?
Source: Cardiovasc Diabetol. 2025 Feb 18;24:80. doi: 10.1186/s12933-025-02640-9 (PMC11837347; doi:10.1186/s12933-025-02640-9)
Supplement: Supplementary file 1 — Additional File 1: Additional File 1. Summary of deviations from the model development plan. Additional File 2. Deviations in the set of features during actual implementation compared to the pre-specified model development plan. Additional File 3. TRIPOD Checklist. Additional File 4. Definition of the study population. Additional File 5. Algorithm for the identification of type 2 diabetes patients. Additional File 6. Description of the outcome selection process. Additional File 7. Selection algorithm for identification of outcomes following [2]: Stroke and myocardial infarction. Additional File 8. Identification of targeted macrovascular complications based on ICD-10-GM codes. Additional File 9. Definition of newly recorded diabetes. Additional File 10. Additional criteria (applied to the run-in period Q1-Q4 2014) to identify prevalent diabetes diagnoses in the observation period. Additional File 11. Potential socio-demographic and socio-economic predictors based on previous models. Additional File 12. Potential predictors related health care resource utilization and costs based on previous models. Additional File 13. Identification of other diabetes-related complications and events based on ICD-10-GM, OPS- and EBM-codes. Additional File 14. Potential predictors related to multimorbidity or constituting dimensions. Additional File 15. Operationalisation of the individual dimensions of the aDSCI score. Additional File 16. Potential comorbidity related predictors based on previous models. Additional File 17. Potential medication related predictors based on previous models: Antidiabetic drugs. Additional File 18. Potential medication related predictors based on previous models: Antithrombotic drugs (Anticoagulants). Additional File 19. Potential medication related predictors based on previous models: Anti-hypertensive drugs. Additional File 20. Potential medication related predictors based on previous models: Lipid modifying agents. Additional File 21. Potential medica [file 12933_2025_2640_MOESM1_ESM.pdf]

Additional Material

Development and validation of prediction models for stroke and myocardial infarction in type 2 diabetes based on health insurance claims – Does machine learning outperform traditional regression approaches?

Anna-Janina Stephan, Ph.D.[1,2], Dr. Michael Hanselmann[1,2], Medina Bajramovic, M.Sc.[1,3], Simon Schosser, M.Sc.[1,3], Michael Laxy, Ph.D[1,2]

[1]Professorship for Public Health and Prevention, TUM School of Medicine and Health, Technical University of Munich, Munich, Germany

[2]German Center for Diabetes Research (DZD), Munich, Germany

[3]Department of Statistics, Ludwig-Maximilians-Universität München, Munich, Germany

Contents

Additional File 1. Summary of deviations from the model development plan 5

Additional File 2. Deviations in the set of features during actual implementation compared to the pre-specified model development plan 8

Additional File 3. TRIPOD Checklist 10

Additional File 4. Definition of the study population 12

Additional File 5. Algorithm for the identification of type 2 diabetes patients (2) 13

Additional File 6. Description of the outcome selection process 14

Additional File 7. Selection algorithm for identification of outcomes following (2): Stroke and myocardial infarction 15

Additional File 8. Identification of targeted macrovascular complications based on ICD-10-GM codes (2) 15

Additional File 9. Definition of newly recorded diabetes 16

Additional File 10. Additional criteria (applied to the run-in period Q1-Q4 2014) to identify prevalent diabetes diagnoses in the observation period 16

Additional File 11. Potential socio-demographic and socio-economic predictors based on previous models 17

Additional File 12. Potential predictors related to health care resource utilization and costs based on previous models 18

Additional File 13. Identification of other diabetes-related complications and events based on ICD-10-GM, OPS- and EBM-codes (2) 19

Additional File 14. Potential predictors related to multimorbidity or constituting dimensions 21

Additional File 15. Operationalisation of the individual dimensions of the aDSCI score 24

Additional File 16. Potential comorbidity related predictors based on previous models 26

Additional File 17. Potential medication related predictors based on previous models: Antidiabetic drugs* 27

Additional File 18. Potential medication related predictors based on previous models: Antithrombotic drugs (Anticoagulants) * 29

Additional File 19. Potential medication related predictors based on previous models: Anti-hypertensive drugs * 30

Additional File 20. Potential medication related predictors based on previous models: Lipid modifying agents * 31

Additional File 21. Potential medication related predictors based on previous models: Cardiac Medications * 32

Additional File 22. Potential medication related predictors based on previous models: CNS-related drugs * 33

Additional File 23. Potential medication related predictors based on previous models: Other drugs * 34

Additional File 24. Feature engineering – technical details 35

Additional File 25. Time windows used for prediction modeling 35

Additional File 26. Software and program codes 35

Additional File 27. Model development – Technical details 35

Additional File 28. Details on parameter tuning 39

Additional File 29. Initial ranges and final values for tuning parameters 39

Additional File 30. Overview and definition of discrimination metrics 40

Additional File 31. Overview and definition of calibration metrics 42

Additional File 32. Overview and definition of classification metrics 43

Additional File 33. Participant flow chart. 45

Additional File 34. Sample characteristics 46

Additional File 35. Training and test set characteristics 47

Additional File 36. Events per variable 47

Additional File 37. Descriptives on the buffer period 48

Additional File 38. Results on discrimination performance 49

Additional File 39. Density plot of predicted MI probability for MI cases and non-cases 50

Additional File 40. Density plot of predicted Stroke probability for Stroke cases and non-cases 51

Additional File 41. Plot of classification metrics for MI against threshold. 52

Additional File 42. Plot of classification metrics for Stroke against threshold. 53

Additional File 43. Results on classification performance at the respective optimized classification threshold 54

Additional File 44. Brier score 55

Additional File 45. Variable Importance for MI models (10 most important variables for each model) 56

Additional File 46. Variable Importance for stroke models (10 most important variables for each model) **57**

Additional File 47. Most important variables for MI prediction models **58**

Additional File 48. Most important variables for stroke prediction models **60**

Additional File 49. Discussion of additional technical aspects **62**

References **63**

Additional File 1. Summary of deviations from the model development plan

**Topic**	**Model development plan pre-specification**	**Actual implementation**	**Rationale for deviation**
**Participant selection**			
Individuals with missing values	Keep in the data set and define a separate "missing value" category or use imputation techniques.	Individuals with missing values on AGS codes for place of living (1.36%) were removed from the data set.	Multiple other variables were derived from this information, introducing perfect collinearity on the missing value category between geographical location, settlement structure types and location of home address district, German Index of multiple deprivation total score and all sub-dimensions in the data set.<br>Risk for biased predictor - outcome associations and biased predictive model performance was considered to be low as missingness mechanism was confirmed to be completely at random by the data provider.<br>Imputation of local area code based on other information in the data not considered sensible.
Individuals with sex categorized as "diverse"	Case not considered.	Removed from the data set.	We decided to remove individuals with diverse sex because this category occurred with a frequency <5 in the data set and inclusion seemed to pose both risk of de-identification and computational challenges.
**Data preparation**			
Variable pool	394 pre-specified features	287 features used during training	Reduction of collinearities, large shares of missing values on single variables, features with little or no observed variation across observations. See **Additional Files 2** and **5-23** for details.
Treatment of negative costs	Exclude.	Negative costs were kept in the data set.	Negative costs usually reflect actual payment corrections and should not generally be considered implausible. A future model would also need to deal with such data in a real-world application.
Treatment of duplicate observations	Exclude.	No exclusions based on duplicate observations.	Case did not occur.
Treatment of missing values	Categorical features with rare cases of missing values → exclude single observations with missing values.	No exclusions based on categorical features with rare cases of missing values	Case did not occur.
**Training & test samples**			
Split ratio	75%:25%.<br>If cross-validation within the training set is computationally infeasible, additionally create a distinct validation data set through a 20% random take-out from the training data set.	80%:20%.<br>For the deep learning models, we used 75% of the training set for training a learner and dealing with early stopping to avoid overfitting and 25% of the training set for hyperparameter tuning.	The initially planned split ratio was adapted to allow an additional comparison with performance of deep learning methods in the sub-project "Moving to next generation Health care – Deep Learning" (MNGHC – DL), which needed a larger training sample. The ratio of the random validation take-out from this training set for tuning was

Topic	Model development plan pre-specification	Actual implementation	Rationale for deviation
			adapted to retain the initially planned training sample size (60%).
**Benchmark**			
Null model	Initially not planned.	Added.	Some performance metrics are more easily interpretable in relation to their values in a null model scenario.
Logistic regression with forward selection	10-fold cross validation within the training set.	No cross validation applied.	Considered not useful, as no parameters were tuned in this model.
Full logistic model	Initially not planned.	Added.	Considered an interesting additional comparison.
**Machine learning models (MNGHC-ML)**			
Random forests loss function	Choice of split points based on the Gini Index.	Choice of split points based on the Hellinger Distance.	Hellinger Distance gives high scores to a split separating the classes in the best way relative to the parent population instead of favoring splits which result in an uneven class distribution (as in the case of Gini Index as split criterion, for example). Therefore, we considered Hellinger Distance more adequate in our case with highly imbalanced outcome data.(1)
Random forests parameter tuning	Iterative variation of each of the values in order to identify the combination which results in the lowest prediction error.	Multiple iterative grid searches with 20 different combinations of these parameters each, selected based on latin hypercube sampling.	A space-filling design that aims to optimally cover the hyperparameter space with a grid was deemed to cover all potentially relevant combinations more efficiently.
Random forests parameter tuning	Using out-of-bag observations	Using 10-fold cross-validation	10-fold cross validation was originally intended for all models and mentioned accordingly in other sections of the protocol.
Random forests variable importance	Defined as the total reduction in Gini index through splits over a given predictor, averaged over all trees: Compare the average Gini index over all trees before and after random permutation of the values of each predictor variable.	Defined as the total reduction in Gini index through splits over a given predictor, averaged over all trees: Averaging each variable's tree-specific contribution to the decrease in node impurity across all trees in the random forest. Impurity is measured by the Gini index.	The original phrasing was equivocal, as one can either assess importance based on permutation (e.g. with AUC) or impurity (with Gini).
**Deep Learning Models (MNGHC-DL)**			
Feature Tokenizer Transformer (FTT)	Optional implementation of the FTT. A simple model will be constructed, and complexity systematically added.	The FTT was implemented and tested for various settings.	Available GPU resources allowed to create and train this architecture.
Multi-Layer-Perceptron (MLP) and Feature Tokenizer Transformer (FTT)	No rescaling of predicted probabilities planned	Testing of Histogram Binning und Temperature Scaling	Predicted probabilities after training with over- and undersampling were not directly interpretable. Temperature Scaling could not adequately bridge the large discrepancies between predictions and observed probabilities and was therefore considered inadequate. Histogram binning was therefore considered as an alternative and assessed as suitable based on maximum calibration error.
**Reporting**			
Guidelines	TRIPOD and STARD	Focus on TRIPOD	STARD is for diagnostic and not prognostic models.
**Model evaluation**			
Reported measures	Youden index	Not reported	The optimized prediction threshold which balances sensitivity and specificity was

Topic	Model development plan pre-specification	Actual implementation	Rationale for deviation
			deemed not very informative for the imbalanced dataset at hand.
Decision curve analysis	optional	not reported	May be reported in another manuscript, would overburden the present one
**Secondary analyses with additional restrictions of the study population**			
to those with incident diabetes	optional	newly recorded diabetes was included as potential feature in the present models	Further secondary analyses would overburden the present manuscript
to healthier diabetic individuals without certain macrovascular diabetes complications (e.g. the target complications) during the observation period.	optional	Target complications during the observation period were included as potential features in the present models	Further secondary analyses would overburden the present manuscript

Additional File 2. Deviations in the set of features during actual implementation compared to the pre-specified model development plan

**Feature category**	**Originally planned number of variables**	**Final number of variables used for training**	**Explanation**
Demographic factors and diabetes history	4	5	3 variables unchanged<br><br>1 three-level factor turned into 2 dummies
Socio-economic indicators	37	12	2 variables for further characterizations of home address districts added → 1 removed again to reduce collinearity<br><br>2 geographical variables removed to reduce collinearity<br><br>24 categorizations of continuous GIMD variables removed to avoid unnecessary categorization and reduce collinearity
Health Care Resource Utilization	141	69	32 GIMD variables pertaining to location of most frequently visited hospital removed due to the large share of missing values (68.18%)<br><br>13 specialist dummies removed due to <15 individuals with visits to the respective specialty<br><br>29 specialist dummies removed due to heterogenous meaning between KVs<br><br>1 variable on number of rehabilitation stays added to ensure congruency with hospital stay operationalization<br><br>1 variable on DMP participation added<br><br>1 alternative USP operationalization added and removed again before training was initialized to avoid perfect correlation
Healthcare costs	8	8	
Complications	17	21	2 variables representing occurrence of the two outcomes in the observation period added<br><br>1 variable for "any complication" added<br><br>1 variable for dialysis as independent feature added
Indices of Multimorbidity	3	7	4 variables added representing two alternative scoring procedures for CCI and EHI, respectively
Specific Comorbidity Index Dimensions	49	49	
Specific Diagnoses / Comorbidities	27	26	Duplicate variable in model development plan
Anti-diabetic drugs	40	23	10 variables removed because the medications they reflected were never prescribed<br><br>3 variables removed for perfect correlation with others<br><br>5 dummy variables merged into 1 new categorical count variable to reduce collinearities
Antithrombotic drugs	11	11	
Anti-hypertensive drugs	14	14	

Lipid modifying agents	9	8	1 variable removed because the medications it reflected were never prescribed
Cardiac Medications	4	4	
CNS-related drugs	15	15	
Other drugs	15	15	
**SUM**	**394**	**287**	

Additional File 3. TRIPOD Checklist

TRIPOD Checklist: Prediction Model Development and Validation

Section/Topic	Item		Checklist Item	Page
**Title and abstract**				
Title	1	D;V	Identify the study as developing and/or validating a multivariable prediction model, the target population, and the outcome to be predicted.	1
Abstract	2	D;V	Provide a summary of objectives, study design, setting, participants, sample size, predictors, outcome, statistical analysis, results, and conclusions.	2
**Introduction**				
Background and objectives	3a	D;V	Explain the medical context (including whether diagnostic or prognostic) and rationale for developing or validating the multivariable prediction model, including references to existing models.	3
	3b	D;V	Specify the objectives, including whether the study describes the development or validation of the model or both.	3,4
**Methods**				
Source of data	4a	D;V	Describe the study design or source of data (e.g., randomized trial, cohort, or registry data), separately for the development and validation data sets, if applicable.	4
	4b	D;V	Specify the key study dates, including start of accrual; end of accrual; and, if applicable, end of follow-up.	4
Participants	5a	D;V	Specify key elements of the study setting (e.g., primary care, secondary care, general population) including number and location of centres.	4
	5b	D;V	Describe eligibility criteria for participants.	4
	5c	D;V	Give details of treatments received, if relevant.	n/a
Outcome	6a	D;V	Clearly define the outcome that is predicted by the prediction model, including how and when assessed.	5
	6b	D;V	Report any actions to blind assessment of the outcome to be predicted.	n/a
Predictors	7a	D;V	Clearly define all predictors used in developing or validating the multivariable prediction model, including how and when they were measured.	5
	7b	D;V	Report any actions to blind assessment of predictors for the outcome and other predictors.	n/a
Sample size	8	D;V	Explain how the study size was arrived at.	4,5
Missing data	9	D;V	Describe how missing data were handled (e.g., complete-case analysis, single imputation, multiple imputation) with details of any imputation method.	5
Statistical analysis methods	10a	D	Describe how predictors were handled in the analyses.	5
	10b	D	Specify type of model, all model-building procedures (including any predictor selection), and method for internal validation.	6
	10c	V	For validation, describe how the predictions were calculated.	6
	10d	D;V	Specify all measures used to assess model performance and, if relevant, to compare multiple models.	6,7
	10e	V	Describe any model updating (e.g., recalibration) arising from the validation, if done.	n/a
Risk groups	11	D;V	Provide details on how risk groups were created, if done.	n/a
Development vs. validation	12	V	For validation, identify any differences from the development data in setting, eligibility criteria, outcome, and predictors.	n/a
**Results**				
Participants	13a	D;V	Describe the flow of participants through the study, including the number of participants with and without the outcome and, if applicable, a summary of the follow-up time. A diagram may be helpful.	7
	13b	D;V	Describe the characteristics of the participants (basic demographics, clinical features, available predictors), including the number of participants with missing data for predictors and outcome.	7
	13c	V	For validation, show a comparison with the development data of the distribution of important variables (demographics, predictors and outcome).	7
Model development	14a	D	Specify the number of participants and outcome events in each analysis.	7
	14b	D	If done, report the unadjusted association between each candidate predictor and outcome.	n/a
Model specification	15a	D	Present the full prediction model to allow predictions for individuals (i.e., all regression coefficients, and model intercept or baseline survival at a given time point).	/
	15b	D	Explain how to the use the prediction model.	/
Model performance	16	D;V	Report performance measures (with CIs) for the prediction model.	7-10
Model-updating	17	V	If done, report the results from any model updating (i.e., model specification, model performance).	n/a
**Discussion**				
Limitations	18	D;V	Discuss any limitations of the study (such as nonrepresentative sample, few events per predictor, missing data).	12-15

Interpretation	19a	V	For validation, discuss the results with reference to performance in the development data, and any other validation data.	/
	19b	D;V	Give an overall interpretation of the results, considering objectives, limitations, results from similar studies, and other relevant evidence.	15
Implications	20	D;V	Discuss the potential clinical use of the model and implications for future research.	15
**Other information**				
Supplementary information	21	D;V	Provide information about the availability of supplementary resources, such as study protocol, Web calculator, and data sets.	4
Funding	22	D;V	Give the source of funding and the role of the funders for the present study.	16

*Items relevant only to the development of a prediction model are denoted by D, items relating solely to a validation of a prediction model are denoted by V, and items relating to both are denoted D;V. We recommend using the TRIPOD Checklist in conjunction with the TRIPOD Explanation and Elaboration document.

Additional File 4. Definition of the study population

Data were provided by the health insurance for all individuals aged ≥18 years with at least one inpatient or verified outpatient E11-E14 diagnosis or an inscription in a diseases management program (DMP) for diabetes mellitus type 1 or type 2 during the period from January 1st 2014 to September 30th 2019 and insurance gaps <30 consecutive days, as German SHIs are legally obliged to cover services during contract gaps of ≤1 month (§ 19 Paragraph 2 Social Code, Book V) (n=912,035).*

Within this roughly pre-filtered data set, we identified individuals with T2D in following a published selection algorithm for Type 2 diabetes patients (**Additional File 5).**(2)

In the outpatient domain, verified ICD-10-GM (International Statistical Classification of Diseases and Related Health Problems, German Modification), diagnoses in at least two out of four consecutive quarters were required for meeting the diabetes definition (M2Q criterion). This requirement was applied to outpatient E11 diagnoses, as shown in the first row of **Additional File 5**, but also incorporated in the combined E11-E14 outpatient diagnoses as specified in the second row of **Additional File 5** and in the E14 outpatient diagnoses as shown in the fifth row ("unspecified with T2D indication") of **Additional File 5**. In addition, requirements for diabetes definition based on outpatient diagnoses were considered fulfilled if there was at least one outpatient verified T2D diagnosis and either an enrollment in a T2D DMP or prescriptions of oral antidiabetics (as reflected in rows 3 and 4 of **Additional File 5**). In the inpatient domain, both primary and secondary diagnoses as well as information on main discharge diagnosis were used. Inpatient diagnoses were attributed to each quarter covered by the respective hospital stay.

For those rows of **Additional File 5** that required more than one criterion to be fulfilled within a time period of four consecutive quarters to include a person as patient with type 2 diabetes, we defined the quarter where the first (as opposed to the last) of these criteria was documented as the start of the diabetes diagnosis. The rationale was that even though as a claims data analyst the second criterion is needed to identify mis- or overcoding in the first instance, for those patients where mis- or overcoding in this instance can be excluded with reasonable certainty, the actual diagnosis in practice existed at least already from the time of first documentation.

For those inpatient cases, where no diagnosis was recorded as primary or secondary diagnosis in the inpatient diagnosis data set, the inpatient main discharge diagnosis available in the inpatient case data set was used instead.

We further restricted the study population to individuals <80 years, as mortality from competing causes in older diabetes patients increases sharply.(3) Additionally, changes in treatment-seeking behaviors for these acute events may occur at high ages, for example in the context of palliative care, potentially decreasing completeness and accuracy of information on stroke and MI.

Exclusion criteria comprised participation in a type 1 diabetes Disease Management Program (DMP), and records for gestational diabetes mellitus (ICD-10 code O24), pancreoprivic diabetes (E13), and pancreatic cancer (C25) (one verified outpatient or one inpatient code was sufficient for exclusion).

* Footnote: Administrative reasons for termination of insurance in Germany other than death comprise 1) moving outside of Germany, or switching the health insurance provider. Provider switches can occur either 2) to another statutory health insurance or 3) to a private health insurance company.

All statutory health insurances in Germany are by law obliged to offer basically the same services, and statutory health insurance contributions are income-dependent and determined by law (i.e. independent from the specific statutory health insurance provider). We consider it reasonable to assume that switches between statutory health insurances and moves to places outside of Germany are independent of individual MI and stroke risk.

Switching to private health insurances is only possible under strict preconditions, including monthly income above a certain high-income threshold (6,150 Euro in 2025) or being self-employed, and is subject to disclosure of one's disease history and health risk behaviors such as cigarette smoking. This information can be used in addition to demographic characteristics such as age to determine the private health insurance tariff. As a consequence, switches to private health insurances are mainly attractive for younger working-age, healthy adults with relatively high socioeconomic status, and less frequent in populations with a chronic disease that becomes more prevalent with age such as type 2 diabetes.

Additional File 5. Algorithm for the identification of type 2 diabetes patients (2)

**Criteria/ diabetes group**	**ICD*-E11 (IPC)**	**ICD-E11 (OPC)**	**ICD-E10 (IPC)**	**ICD-E10 (OPC)**	**ICD-E14 (IPC)**	**ICD-E14 (OPC)**	**OAD†**	**DMP type 2**
**(1) Type 2**	(≥1 OR	≥2) AND	0 AND	0				
		≥1 AND	0 AND	0 AND	(≥1 OR	≥1)		
		≥1 AND	0 AND	0 AND			(Yes OR	Yes)
**(2) Unclear with type 2 indication**	(≥1 OR	≥1) AND	(≥1 OR	≥1)		AND	(Yes OR	Yes)
**(3) Unspecified with type 2 indication**	0 AND	0 AND	0 AND	0 AND	(≥1 AND	≥2) AND	(Yes OR	Yes)

* E10, type 1 diabetes; E11, type 2 diabetes, E14, unspecified diabetes.
† At least one prescription of oral antidiabetics (ATC-code A10B) in the rolling 1-year run-in period.
Abbrevations: DMP, disease management program; ICD, international classification of diseases; ipc, inpatient care; OAD, oral antidiabetic drugs; opc, outpatient care.

After applying eligibility criteria and removing individuals who missed crucial information as described in **Additional File 1**, n=371,006 individuals from the original data set delivery (n=912,035) had a validated diagnosis of type 2 diabetes (**Additional File 34**).

Additional File 6. Description of the outcome selection process

Based on an expert consultation with two leading endocrinologists from Germany, we decided to focus on prediction of stroke and myocardial infarction. In this expert consultation, the suitability of various microvascular (retinopathy, blindness, renal insufficiency, end stage renal disease, amputation, and neuropathy including diabetic foot syndrome, peripheral angiopathy, polyneuropathy) and macrovascular (angina pectoris, congestive heart failure, myocardial infarction, other ischemic heart diseases, and stroke) complications for this research project was discussed. Criteria along which suitability of outcomes for the project was assessed included 1) preventability, 2) time needed to take effective preventive measures, 3) ease of clinical detection, 4) possibility of defining complication onset and 5) burden on the health care system. The first criterion was applied to make sure the ~~algorithm~~ model would aim at identifying a complication which would in theory be preventable with clinical treatment or lifestyle changes. With the second criterion we aimed at complications against which preventive action would still be effective if taken within a relatively short time period before the event. The third criterion was supposed to identify complications for which an alert by an algorithm would provide additional information to those apparent to the treating physician, such as visible (clinical) patient characteristics. The fourth criterion implied that it should be possible to clearly identify the complication as either present or not present with reasonable certainty using claims data information. This criterion would for example be violated for complications which reflect a gradually deteriorating process where some patients might be detected and diagnosed at earlier stages than others as opposed to acute emerging events. Finally, with the fifth criterion, we tried to target more frequent and/or more expensive complications over less frequent and/or less expensive complications.

Additional File 7. Selection algorithm for identification of outcomes following (2): Stroke and myocardial infarction

Identification of the selected complications in the available claims data sets followed the ICD-10-GM codes used in a previous analysis (**Additional File 8**).(2) As stroke and myocardial infarction represent acute macrovascular complications which usually require inpatient treatment, according to this algorithm they were defined solely through inpatient records with primary diagnosis in order to reduce the potential for outcome misclassification.(4)

Additional File 8. Identification of targeted macrovascular complications based on ICD-10-GM codes (2)

**Cardiovascular complications**	
Myocardial infarction/cardiac arrest	ICD-codes I21 (acute myocardial infarction) OR<br>I46.0 or .9 (cardiac arrest)
**Cerebrovascular complications**	
Stroke*	ICD-codes I60 (subarachnoidal haemorrhage) OR<br>I61 (intracerebral bleeding) OR<br>I62 (other non-traumatic intracranial bleeding) OR<br>I63 (brain infarction) OR<br>I64 (stroke)

*Stroke includes bleeding inside the brain (hemorrhagic stroke).
Abbreviations: GM, German modification; ICD, International Classification of Diseases.

Additional File 9. Definition of newly recorded diabetes

New diabetes records were defined as including all patients who did not have exclusion criteria as defined for the observation period (participation in a DMP for type 1 diabetes, records for gestational diabetes mellitus (ICD-10 O24), pancreoprivic diabetes (E13), and pancreatic cancer (C25)) in a one-year run-in period between Q1 and Q4 2014 and who did additionally not have any diabetes-related ICD-10 records (E10-E14) as defined in **Table S5** during this run-in period.

Additional File 10. Additional criteria (applied to the run-in period Q1-Q4 2014) to identify prevalent diabetes diagnoses in the observation period

**ICD*-E11 (IPC)**	**ICD-E11 (OPC)**	**ICD-E10 (IPC)**	**ICD-E10 (OPC)**	**ICD-E14 (IPC)**	**ICD-E14 (OPC)**	**OAD†**	**DMP type 2**	**Insulin**
(≥1 OR	≥2 OR	≥1 OR	≥2 OR	≥1 OR	≥2 OR	Yes OR	Yes OR	Yes
	1 AND				1			
			1 AND		1			
	1 AND		1					

* E10, type 1 diabetes; E11, type 2 diabetes, E14, unspecified diabetes.
† At least one prescription of oral antidiabetics (ATC-code A10B) in the rolling 1-year run-in period.
Abbrevations: DMP, disease management program; ICD, international classification of diseases; ipc, inpatient care; OAD, oral antidiabetic drugs; opc, outpatient care.

Additional File 11. Potential socio-demographic and socio-economic predictors based on previous models

**Demographic factors and diabetes history**	
Age	
Sex	
Diabetes disease duration: newly recorded ("incident") / prevalent diagnosis *	
Diabetes subtype at inclusion (T2D, unclear with T2D indication) §‡	
**Socio-economic indicators**	**Reference**
Geographical location (federal states: BW, BY, BE, BB, HB, HH, HE, MV, NI, NW, RP, SL, SN, ST, SH, TH) †‖	
Settlement structure types of home address district: free urban municipalities, urban districts, rural districts with population concentrations, sparsely populated rural districts ("siedlungsstrukturelle Kreistypen": Kreisfreie Großstädte, Städtische Kreise, Ländliche Kreise mit Verdichtungsansätzen, Dünn besiedelte ländliche Kreise) †	
Location of home address district: central, very central, peripheral, very peripheral§	
German Index of multiple deprivation on district level (total score) †¶	(5-8)
German Index of multiple deprivation on district level (income dimension) †¶	
German Index of multiple deprivation on district level (employment dimension) †¶	
German Index of multiple deprivation on district level (education dimension) †¶	
German Index of multiple deprivation on district level (communal income dimension) †¶	
German Index of multiple deprivation on district level (social capital dimension) †¶	
German Index of multiple deprivation on district level (environmental dimension) †¶	
German Index of multiple deprivation on district level (security dimension) †¶	
Insurance status (employed, caregiver, unemployed recipient of social benefits, voluntarily insured, pensioner, other, family insurance coverage, employment-seeking recipient of social benefits) †#	

* For a definition of newly recorded diabetes, see **Text S9 and Table S10**.
† Exploratory features not identified through use in previous models
‡ Feature "unspecified with T2D indication" was removed from the dataset due to frequency <15
§ Feature added after publication of model development plan before start of model development
‖ Alternative operationalizations for geographical location (former East vs. West German areas and North/South/East/West Germany) and characterization of home address district as rural/urban (tentatively introduced after finalization of model development plan) were removed from the final data set to reduce collinearity issues.
¶ GIMD total score and subscores were used as continuous variables. The originally additionally planned less informative categorical tertiles, quintiles and deciles of the variable were removed from the final data set to reduce collinearity issues.
# Categories "student" and "other" were merged due to frequency <10 in category "student"

Additional File 12. Potential predictors related to health care resource utilization and costs based on previous models

**Health Care Resource Utilization***	**Reference**
Number of hospital stays †	(9, 10)
Total number of days spent in hospital ‡	
Average duration of hospital stays	(9)
Number of outpatient physician visits in total and by physician specialty (defined as days with EBM codes) §	(9)
Number of emergency department visits	(8-10)
Number of rehabilitation stays ¶	
Total number of days spent in rehabilitation ‡	
Average duration of rehabilitation stays ‡	
Number of prescriptions for podologists: Heilmittelpositionsnummernverzeichnis code 7XXXX – 7XXXX ‡	
Number of prescriptions for physiotherapists: Heilmittelpositionsnummernverzeichnis codes 2XXXX) ‡	
Continuity of Care as measured by the Usual Provider Index (UPC) ‡ ||	(11)
T2D DMP participation ¶	
**Healthcare costs**	
Overall health care costs #	(9)
Inpatient healthcare costs ‡ #	
Outpatient healthcare costs #	(9)
Costs of outpatient pharmaceuticals #	(9)
Costs of devices ‡ #	
Costs of aids & remedies ‡ #	
Ambulatory inpatient healthcare costs ‡ #	
Costs of rehabilitation ‡ #	

* Feature “Hospital location area deprivation of most frequently visited hospital”(5-7) excluded from final data set due to large numbers of missing values (68.18%)

† For the number of hospital stays, we counted every episode separately where a) a stay was initiated directly after the completion of the previous stay (counted as two separate stays) or b) a shorter stay was recorded in the middle of a longer stay (counted as three separate stays)

‡ Exploratory features not identified through use in previous models

§ The number of visits to the following physician specialties were removed from the final data set due to <15 individuals with visits to the respective specialty: geriatrician, infectiologist, children’s hematologist or -oncologist, children’s cardiologist, neonatologist, neuropediatrician, children’s pneumologist, children’s hematologist or -oncologist (specialist), children’s cardiologist (specialist), neonatologist (specialist), children’s cardiologist (specialist), neuropediatrician (specialist), forensic psychiatrist. In addition, visits to specialties with KV specific codes and therefore no global common meaning were also not considered.

|| Two different categorical variables were created for this index: USP Index > 0.8 (high continuity of care), Index > 0.50 and ≤ 0.8 USP (medium continuity of care), USP Index ≤ 0.5 (low continuity of care), less than 3 outpatient visits (low outpatient care users);(12) Tertiles plus “less than 3 outpatient visits (low outpatient care users)” category.(13) The latter operationalization was again removed from the dataset before training was initialized to reduce collinearities.

¶ Feature added after publication of model development plan before start of model development

Changed into a continuous variable before start of model development instead of the quartiles originally noted in the model development plan to avoid unnecessary categorization

Additional File 13. Identification of other diabetes-related complications and events based on ICD-10-GM, OPS- and EBM-codes (2)

**Any Complication***	
**Microvascular complications**	
**Eye complications**	
Retinopathy	ICD-codes E1*.3- (diabetes with eye complications) OR<br>H36.0 (diabetic retinopathy) OR<br>H35.0 (background retinopathy and retinal vascular changes) OR<br>H35.2 (other proliferative retinopathy) OR
Blindness in one or two eyes	ICD-codes H54.0 (blindness, both eyes) OR H54.4 (blindness, one eye)
**Renal complications**	
Renal insufficiency	ICD-codes E1*.2- (diabetes with renal complications) OR<br>N17 (acute renal failure) OR<br>N18 (chronic renal failure, without N18.5) OR<br>N19 (not other specified renal failure)
ESRD	ICD-code N18.5 (terminal renal insufficiency)
➔ with or without dialysis †	ICD-codes Z49 (dialysis) OR<br>Z99.2 (long-term dialysis in renal insufficiency) OR<br><br>OPS-codes 8-854 (hemodialysis) OR<br>8-855 (hemodiafiltration) OR<br>8-857 (peritoneal dialysis) OR<br>8-85a (dialysis after failed kidney transplant) OR<br>EBM-codes 13602-13622 w/o 13621 (dialysis fees) OR<br>40815-40838 (material cost fee)
**Neuropathic complications**	
Diabetic foot syndrome (with polyneuropathy and peripheral angiopathy)	ICD-codes E1*.74 and .75 (diabetes with multiple complications, with diabetic foot syndrome)<br>OR<br>ICD-code for peripheral neuropathy G63.2 (diabetic polyneuropathy)<br>AND<br>one of the ICD-codes for PVD: E1*.5 (diabetes with peripheral vascular complications) OR<br>I70.2 (atherosclerosis of extremities) OR<br>I73.9 (peripheral vascular disease, not other specified) OR<br>I79.2 (diabetic peripheral angiopathy) OR<br>R02 (gangrene)<br>OR<br>EBM-code 02311 (treatment of diabetic foot)
Amputation of lower extremities	OPS-codes 5-864 (amputation of lower extremity)<br>5-865 (amputation of the foot)
**Macrovascular complications**	
**Cardiovascular complications**	
Angina pectoris	ICD-code I20 (angina pectoris)
Chronic heart failure (CHF)	ICD-codes I50 (heart failure)<br>I11.0 (hypertensive heart disease with heart failure),<br>I13.0 (hypertensive heart and chronic kidney disease with heart failure),<br>I13.2 (Hypertensive heart and chronic kidney disease with heart failure and with end stage renal disease)
Other IHD	ICD-codes I22 (recurrent myocardial infarction),<br>I24 (other acute ischemic heart disease),<br>I25 (chronic ischemic heart disease)
Abbreviations: CHF, chronic heart failure; EBM, uniform value scale for outpatient services; ESRD, end-stage renal disease; GM, German Modification; ICD, International Classification of Diseases; IHD, ischemic heart disease; OPS, operation procedure codes; PVD, peripheral vascular disease.<br>* Feature added after publication of model development plan before start of model development	

† Dialysis was additionally added as independent feature after publication of the model development plan and before start of model development

Additional File 14. Potential predictors related to multimorbidity or constituting dimensions

**Indices of Multimorbidity**		**Reference**
Charlson Comorbidity Index (CCI)* - unweighted count of dimensions (14) †		(15-17)
Charlson Comorbidity Index (CCI)* - original dimension weights based on (15, 16)		
Charlson Comorbidity Index (CCI)* - updated dimension weights based on (18) †		
Elixhauser Index (EHI)* - unweighted count of groups ‡†		(15, 19)
Elixhauser Index (EHI)* - original weights based on (20)		
Elixhauser Index (EHI)* - updated Swiss weights based on (21) †		
Adapted Diabetes Complications Severity Index (aDCSI)§		(2, 22, 23)
**Specific Comorbidity Index Dimensions**	**ICD-codes**	**Reference**
CCI dimension: Myocardial Infarction*	I21, I22, I25.2	
CCI / EHI dimension: Congestive heart failure	I43, I50, I09.9, I11.0, I13.0, I13.2, I25.5, I42.0, I42.5, I42.6, I42.7, I42.8, I42.9, P29.0	
CCI /EHI dimension: Peripheral vascular disease	I70, I71, I73.1, I73.8, I73.9, I77.1, I79.0, I79.2, K55.1, K55.8, K55.9, Z95.8, Z95.9	
CCI dimension: Cerebrovascular Disease*	G45, G46, I60, I61, I62, I63, I64, I65, I66, I67, I68, I69, H34.0	
CCI / EHI dimension: Chronic pulmonary disease	J40, J41, J42, J43, J44, J45, J46, J47, J60, J61, J62, J63, J64, J65, J66, J67, I27.8, I27.9, J68.4, J70.1, J70.3	
CCI dimension: Dementia	F00, F01, F02, F03, G30, F05.1, G31.1	
CCI / EHI dimension: Paralysis	G81, G82, G04.1, G11.4, G80.1, G80.2, G83.0, G83.1, G83.2, G83.3, G83.4, G83.9	
CCI dimension: Diabetes without sequelae*	E10.0, E10.1, E10.6, E10.8, E10.9, E11.0, E11.1, E11.6, E11.8, E11.9, E12.0, E12.1, E12.6, E12.8, E12.9, E13.0, E13.1, E13.6, E13.8, E13.9, E14.0, E14.1, E14.6, E14.8, E14.9	
CCI dimension: Diabetes with sequelae*	E10.2, E10.3, E10.4, E10.5, E10.7, E11.2, E11.3, E11.4, E11.5, E11.7, E12.2, E12.3, E12.4, E12.5, E12.7, E13.2, E13.3, E13.4, E13.5, E13.7, E14.2, E14.3, E14.4, E14.5, E14.7	
CCI dimension: Renal disease	N18, N19, N05.2, N05.3, N05.4, N05.5, N05.6, N05.7, N25.0, I12.0, I13.1, N03.2, N03.3, N03.4, N03.5, N03.6, N03.7, Z49.0, Z49.1, Z49.2, Z94.0, Z99.2	
CCI dimension: Mild Liver Disease	B18, K73, K74, K70.0, K70.1, K70.2, K70.3, K70.9, K71.7, K71.3, K71.4, K71.5, K76.0, K76.2, K76.3, K76.4, K76.8, K76.9, Z94.4	
CCI dimension: Moderate-severe liver disease	K704, K71.1, K72.1, K72.9, K76.5, K76.6, K76.7, I85.0, I85.9, I86.4, I98.2	
CCI dimension: Cancer	C00, C01, C02, C03, C04, C05, C06, C07, C08, C09, C10, C11, C12, C13, C14, C15, C16, C17, C18, C19, C20, C21, C22, C23, C24, C25, C26, C30, C31, C32, C33, C34, C37, C38, C39, C40, C41, C43, C45, C46, C47, C48, C49, C50, C51, C52, C53, C54, C55, C56, C57, C58, C60, C61, C62, C63, C64, C65, C66, C67, C68, C69, C70, C71, C72, C73, C74, C75, C76, C81, C82, C83, C84, C85, C88, C90, C91, C92, C93, C94, C95, C96, C97	
CCI / EHI dimension: Metastatic Carcinoma	C77, C78, C79, C80	
CCI dimension: Ulcers	K25, K26, K27, K28	
CCI dimension: Rheumatoid arthritis	M05, M32, M33, M34, M06, M31.5, M35.1, M35.3, M36.0	
CCI / EHI dimension: AIDS/HIV	B20, B21, B22, B24	

EHI dimension: Cardiac Arrhythmia	I44.1, I44.2, I44.3, I45.6, I45.9, I47, I48, I49, R00.0, R00.1, R00.8, T82.1, Z45.0, Z95.0	
EHI dimension: Valvular disease	A52.0, I05, I06, I07, I08, I09.1, I09.8, I34, I35, I36, I37, I38, I39, Q23.0, Q23.1, Q23.2, Q23.3, Z95.2, Z95.3, Z95.4	
EHI dimension: Pulmonary circulation disease	I26, I27, I28.0, I28.8, I28.9	
EHI dimension: Hypertension uncomplicated	I10	
EHI dimension: Hypertension complicated	I11, I12, I13, I15	
EHI dimension: Other neurological disorders	G10, G11, G12, G13, G20, G21, G22, G25.4, G25.5, G31.2, G31.8, G31.9, G32, G35, G36, G37, G40, G41, G931, G93.4, R47.0, R56	
EHI dimension: Diabetes uncomplicated*	E10.0, E10.1, E10.9, E11.0, E11.1, E11.9, E12.0, E12.1, E12.9, E13.0, E13.1, E13.9, E14.0, E14.1, E14.9	
EHI dimension: Diabetes complicated*	E10.2, E10.3, E10.4, E10.5, E10.6, E10.7, E10.8, E11.2, E11.3, E11.4, E11.5, E11.6, E11.7, E11.8, E12.2, E12.3, E12.4, E12.5, E12.6, E12.7, E12.8, E13.2, E13.3, E13.4, E13.5, E13.6, E13.7, E13.8, E14.2, E14.3, E14.4, E14.5, E14.6, E14.7, E14.8	
EHI dimension: Hypothyroidism	E00, E01, E02, E03, E89.0	
EHI dimension: Renal failure	I12.0, I13.1, N18, N19, N25.0, Z49.0, Z49.1, Z49.2, Z94.0, Z99.2	
EHI dimension: Liver disease	B18, I85, I86.4, I98.2, K70, K71.1, K71.3, K71.4, K71.5, K71.7, K72, K73, K74, K76.0, K76.2, K76.3, K76.4, K76.5, K76.6, K76.7, K76.8, K76.9, Z94.4	
EHI dimension: Peptic ulcer disease excluding bleeding	K25.7, K25.9, K26.7, K26.9, K27.7, K27.9, K28.7, K28.9	
EHI dimension: Lymphoma	C81, C82, C83, C84, C85, C88, C96, C90.0, C90.2	
EHI dimension: Solid Tumor without Metastasis	C00, C01, C02, C03, C04, C05, C06, C07, C08, C09, C10, C11, C12, C13, C14, C15, C16, C17, C18, C19, C20, C21, C22, C23, C24, C25, C26, C30, C31, C32, C33, C34, C37, C38, C39, C40, C41, C43, C45, C46, C47, C48, C49, C50, C51, C52, C53, C54, C55, C56, C57, C58, C60, C61, C62, C63, C64, C65, C66, C67, C68, C69, C70, C71, C72, C73, C74, C75, C76, C97	
EHI dimension: Rheumatoid arthritis/collagen	L94.0, L941, L94.3, M05, M06, M08, M12.0, M12.3, M30, M31.0, M31.1, M31.2, M31.3, M32, M33, M34, M35, M45, M46.1, M46.8, M46.9	
EHI dimension: Coagulopathy	D65, D66, D67, D68, D69.1, D69.3, D69.4, D69.5, D69.6	
EHI dimension: Obesity	E66	(2, 10, 24-27)
EHI dimension: Weight loss	E40, E41, E42, E43, E44, E45, E46, R63.4, R64	
EHI dimension: Fluid and electrolyte disorders	E22.2, E86, E87	
EHI dimension: Chronic blood loss anemia	D50.0	
EHI dimension: Deficiency anemias	D50.8, D50.9, D51, D52, D53	
EHI dimension: Alcohol abuse	F10, E52, G62.1, I42.6, K29.2, K70.0, K70.3, K70.9, T51, Z50.2, Z71.4, Z72.1	
EHI dimension: Drug abuse	F11, F12, F13, F14, F15, F16, F18, F19, Z71.5, Z72.2	
EHI dimension: Psychoses	F20, F22, F23, F24, F25, F28, F29, F30.2, F31.2, F31.5	

EHI dimension: Depression	F20.4, F31.3, F31.4, F31.5, F32, F33, F34.1, F41.2, F43.2

* The EHI and CCI will be calculated including all 31 and 17 original dimensions, respectively, even though both the EHI and CCI diabetes dimensions comprise codes that qualify for inclusion criteria in the overall study population.
† Alternative index scores resulting from applying different dimension weights were added as independent features after publication of the model development plan and before start of model development
‡ Exploratory features not identified through use in previous models
§ A detailed list of codes and weights used for the aDSCI dimensions can be found in Table S15

Additional File 15. Operationalisation of the individual dimensions of the aDSCI score

Complication (aDCSI dimension)	Diagnosis Label	ICD-10 Code	Points
Ocular	Diabetes with eye complications	E1*.3-	1
	Diabetic cataract	H28.0	1
	Diabetic retinopathy	H36.0	1
	Background retinopathy and retinal vascular changes	H35.0	1
	Other specified retinal disorders	H35.8	1
	Degeneration of macula and posterior pole	H35.3	1
	Retinal hemorrhage	H35.6	1
	Retinal disorder, unspecified	H35.9	1
	Other proliferative retinopathy	H35.2	2
	Vitreous hemorrhage	H43.1	2
	Retinal detachments and breaks	H33	2
	Visual impairment including blindness (binocular or monocular)	H54.-	2
Renal	Acute nephritic syndrome	N00	1
	Chronic nephritic syndrome	N03	1
	Nephrotic syndrome	N04	1
	Unspecified nephritic syndrome	N05	1
	Glomerular disorders in diabetes mellitus	N08.3	1
	Diabetes with renal complications	E1*.2	1
	Acute renal failure	N17	2
	Chronic renal failure	N18	2
	Not other specified renal failure	N19	2
	Disorder of kidney and ureter, unspecified	N28.9	2
Neurological	Diabetic polyneuropathy	G63.2	1
	Diabetes mellitus with neurological complications	E1x.4	1
	Diabetic arthropathy	M14.2	1
	Neuropathy arthropathy	M14.6	1
	Mononeuropathies of upper limb	G56	1
	Mononeuropathies of lower limb	G57	1
	Other mononeuropathies	G58	1
	Diabetic mononeuropathy	G59.0	1
	Hereditary and idiopathic neuropathy, unspecified	G60.9	1
	Myasthenic syndromes in endocrine diseases	G73.0	1
	Myasthenic syndromes in other diseases classified elsewhere	G73.3	1
	Autonomic neuropathy in endocrine and metabolic diseases	G99.0	1
	Injury of oculomotor nerve	S04.1	1
	Injury of trochlear nerve	S04.2	1
	Injury of abducent nerve	S04.4	1
	Third [oculomotor] nerve palsy	H49.0	1
	Fourth [trochlear] nerve palsy	H49.1	1
	Sixth [abducent] nerve palsy	H49.2	1
Cerebrovascular	Transient cerebral ischaemic attacks and related syndromes	G45	1
	Intracerebral haemorrhage	I61	2
	Cerebral infarction	I63	2
	Stroke, not specified as haemorrhage or infarction	I64	2
	Occlusion and stenosis of precerebral arteries, not resulting in cerebral infarction	I65	2
	Occlusion and stenosis of cerebral arteries, not resulting in cerebral infarction	I66	2

	Other cerebrovascular diseases	I67	2
	Sequelae of cerebrovascular disease	I69	2
Cardiovascular	Angina pectoris	I20	1
	Other acute ischaemic heart diseases	I24	1
	Chronic ischaemic heart disease	I25	1
	Atherosclerosis	I70	1
	Heart failure	I50	2
	Acute myocardial infarction	I21	2
	Certain current complications following acute myocardial infarction	I23	2
	Cardiac arrest	I46	2
	Ventricular fibrillation and flutter	I49.0	2
	Subsequent myocardial infarction	I22	2
	Atherosclerosis of arteries of extremities - pelvis-leg type with gangrene	I70.24	2
	Atherosclerosis of arteries of extremities - pelvis-leg type with ulceration	I70.25	2
	Paroxysmal tachycardia	I47	2
	Atrial fibrillation and flutter	I48	2
	Old myocardial infarction	I25.2	2
	Aortic aneurysm and dissection	I71	2
Peripheral vascular	Diabetes with peripheral vascular complications	E1*.5	1
	Aneurysm and dissection of artery of lower extremity	I72.4	1
	Peripheral angiopathy in diseases classified elsewhere	I79.2	1
	Other disorders of arteries, arterioles and capillaries in diseases classified elsewhere	I79.8	1
	Peripheral vascular disease, unspecified	I73.9	1
	Peripheral vascular disease, unspecified	I73.9	1
	Open wound of ankle and foot	S91.3	1
	Ulcer of lower limb, not elsewhere classified	L97	2
	Embolism and thrombosis of arteries of lower extremities	I74.3	2
	Gangrene, not elsewhere classified	R02	2
	Gas gangrene	A48.0	2
Metabolic	diabetes mellitus with ketoacidosis	E1*.1	2
	diabetes mellitus with coma	E1*.0	2

The ICD-10 codes used to operationalize the aDCSI dimensions were derived from three recent and independently published ICD-10 translations of the ICD-9 codes included in the DCSI(23) and aDCSI scores:(2, 22) While Kähm et al. and Wicke et al. provided ICD-10-GM translations of codes included in the original aDSCI(28) (thus an adaptation of the original DCSI which does not rely on additional laboratory results usually unavailable in claims data), Glasheen et al. provided an provided a refined ICD-10-CM translation of the original DCSI index,(29) which consisted of both laboratory values and ICD-9 diagnoses. ICD-10 codes were included in the aDCSI for our analysis if they were present in at least one out of the three recent ICD-10 translations. If a code was present in only one of the ICD-10 translations, but a sub-code present in another translation, we used the higher-level code, unless only the sub-code was specifically targeting a diabetes complication whereas the rest of the higher-level code was not. If ICD-10 codes were only suggested in a DCSI translation but the corresponding ICD-9 code could not be found in the original ICD-9 code list by Chang et al, they were disregarded. Codes used already in one complication dimension were not re-used in another complication dimension.

Additional File 16. Potential comorbidity related predictors based on previous models

Specific Diagnoses / Comorbidities	ICD-codes	Reference
Derailed diabetes (derailment of glucose metabolism)	E10-E14 plus 1, 3 or 5 as fifth digit	(2)
Severe hypoglycemic events	E16.x, E11.63, E13.63, E14.63	(10, 17)
Diabetes without complications	E10-E14 plus 9 as fourth digit	(2)
Hypertension	I10-I15	(2, 17, 24, 25, 27, 30-36)
Hypotension	I95	(23)
Mental and behavioural disorders due to use of alcohol*	F10	(2)
Mental and behavioural disorders due to use of tobacco†	F17	(2, 24, 25, 31-34, 37)
Depression	F32, F33, F34	(2, 10)
Cancer	C00-C99	(2)
Sleeping disorder	G47, F51	(2)
Proteinuria (incl. albuminuria)	R80, N39.1, N39.2, N06	(26, 27, 35, 37-41)
Tinea pedis	B35.3	(42)
Onychomycosis	B35.1, B37.2	(42)
Non-high-density lipoprotein cholesterol / Dyslipidemia	E78	(17, 31, 34-37, 41)
Atrial fibrillation	I48	(33, 35, 43)
Arterial embolism and thrombosis	I74	(44)
Left ventricular hypertrophy	I51.7	(45)
Right and left bundle branch block	I45.0, I45.1	(46)
Anemia	D60-64	(43)
COPD	J44	(36)
Lower urinary tract dysfunction ‡	N31.1, N31.2, N31.9	(23)
Diabetic liver ‡	K77.8	
Functional diarrhoea	K59.1	(23)
Other polyneuropathies and disorders of autonomic nervous system	G62, G90.09, G90.8 G90.9	(2, 23)
Kidney transplant	Renal transplant: OPS 5-555, Kidney transplant failure and rejection: T86.1, Kidney transplant status: Z94.0, Additional lump sum for treatment of a kidney or kidney-pancreas transplant carrier: EBM 13601	(47)
Cataract	H28: Diabetic cataract H26.9 Cataract, unspecified	

* Relabelled from “Hazardous alcohol consumption” in the model development plan to be more precise.
† Relabelled from “Hazardous smoking” in the model development plan to be more precise.
‡ Exploratory features not identified through use in previous models

Additional File 17. Potential medication related predictors based on previous models: Antidiabetic drugs*

Variable	Variable levels	Definition (ATC Codes)	Reference
**Anti-diabetic drugs †**			
Number and type of anti-diabetic medication categories ‡	0-3	Insulin only: A10A NOT (A10AE56 OR A10AE54) NOT A10B<br>OAD only: A10B NOT A10A<br>Both insulin and other glucose-lowering medication: (A10A AND A10B), (A10AE56 OR A10AE54)	(2, 8, 17, 36)
Any glucose-lowering medication excluding insulin	0, 1	A10B	(26, 36, 48)
Any insulin	0, 1	A10A	(8, 10, 17, 26, 36, 48, 49)
**Specific glucose lowering medication groups excluding insulin §**			
Number of specific glucose lowering medication groups (excluding insulin)	0-11	A10BF, A10BA, A10BH, A10BJ, A10BK, A10BB, A10BC, A10BG, A10BX05, (A10BX03 OR A10BX02), (A10BX01 OR A10BX06 OR A10BX15)	(10, 17)
Any alpha-glucosidase inhibitors single	0, 1	A10BF	
Any Biguanides/Non-sulfonylureas single	0, 1	A10BA	(10, 49)
Any Biguanides/Non-sulfonylureas single or in combination	0, 1	A10BA OR A10BD01 OR A10BD17 OR A10BD13 OR A10BD16 OR A10BD15 OR A10BD20 OR A10BD23 OR A10BD22 OR A10BD18 OR A10BD11 OR A10BD26 OR A10BD05 OR A10BD14 OR A10BD03 OR A10BD10 OR A10BD07 OR A10BD02 OR A10BD08 OR A10BD27 OR A10BD25	
Any Dipeptidyl peptidase 4 (DDP-4) inhibitors single	0, 1	A10BH	(8, 10)
Any Dipeptidyl peptidase 4 (DDP-4) inhibitors single or in combinations	0, 1	A10BH OR A10BD13 OR A10BD09 OR A10BD07 OR A10BD24 OR A10BD12 OR A10BH51 OR A10BD08 OR A10BD18 OR A10BH52 OR A10BD11 OR A10BD27 OR A10BD19 OR A10BD10 OR A10BD25 OR A10BD21 OR A10BD22	
Any Glucagon-like peptide-1 (GLP-1) analogues (Incretin mimetics)	0, 1	A10BJ	(8, 10)
Any Sodium-glucose co-transporter 2 (SGLT-2) inhibitors single	0, 1	A10BK	(10)
Any Sodium-glucose co-transporter 2 (SGLT-2) inhibitors single or in combination	0, 1	A10BK OR A10BD16 OR A10BD15 OR A10BD25 OR A10BD21 OR A10BD20 OR A10BD27 OR A10BD19 OR A10BD23 OR A10BD24	
Thiazolidinediones single	0, 1	A10BG	(8, 10)

Thiazolidinediones single or in combination	0, 1	A10BG OR A10BD03 OR A10BD04 OR A10BD05 OR A10BD06 OR A10BD09 OR A10BD12 OR A10BD26	
Any Meglitinides single	0, 1	A10BX03, A10BX02	
Any antidiabetic combination	0, 1	A10BD	(17)
**Specific insulin groups §**			
Any Insulins and analogues for injection, fast-acting	0, 1	A10AB01, A10AB02, A10AB03, A10AB30	(8, 10)
Any Rapid acting insulin	0, 1	A10AB04, A10AB05, A10AB06	(8, 10)
Any Insulins and analogues for injection, intermediate-acting	0, 1	A10AC	(8)
Any Insulins and analogues for injection, intermediate- or long-acting combined with fast-acting	0, 1	A10AD	(8)
Any Insulins and analogues for injection, long-acting	0, 1	A10AE	(8, 10)
Any Insulin and antidiabetic drug	0,1	A10AE56, A10AE54	
Any second-generation Sulfonylurea single	0, 1	A10BB01, A10BB04, A10BB09, A10BB12, A10BB07, A10BB08, A10BB11	(8)

*Listed variables were derived from international literature in combination with the WHO ATC catalogue without considering the current availability or prescription frequencies of specific drugs/drug classes in Germany. Certain drugs/drug classes may therefore not be prescribed to any patient in the data set.

† Variables “Other anti-diabetic medication type (A10X)”, “Any Sulfonylurea single or in combination (A10BB OR A10BD01 OR A10BD02 OR A10BD06 OR A10BD04)”,(8, 10, 49) “Any Sulfonamide (heterocyclic) (A10BC)”, “Any Amylin analogs (A10BX05)”(8, 10), “Any other glucose lowering medication groups (excluding insulin) (A10BX01, A10BX06, A10BX15)”, “Any Aldose reductase inhibitors (A10XA)”, “Any first-generation Sulfonylurea single (A10BB31, A10BB06, A10BB02, A10BB10, A10BB05, A10BB03”,(8) “Any Insulins and analogues for inhalation (A10AF)”, “Any alpha-glucosidase inhibitors in combination (A10BD17)”(8, 10), “Any Meglitinides in combination (A10BD14)”(8, 10, 49) were removed from the final data set because the respective medications were never prescribed. Variables “Metformin single” (A10BA02) and “Metformin single or in combination (A10BA02 OR A10BD17 OR A10BD13 OR A10BD16 OR A10BD15 OR A10BD20 OR A10BD23 OR A10BD22 OR A10BD18 OR A10BD11 OR A10BD26 OR A10BD05 OR A10BD14 OR A10BD03 OR A10BD10 OR A10BD07 OR A10BD02 OR A10BD08 OR A10BD27 OR A10BD25)“(8, 10) and “Any Sulfonylurea single (A10BB)”(8, 10, 49) were removed from the final dataset for perfect correlation with variables “Any Biguanides/Non-sulfonylureas single”, “Any Biguanides/Non-sulfonylureas single or in combination” and “Any second-generation Sulfonylurea single”, respectively.

‡ New categorical variable created merging information from originally planned dummy variables: “Insulin only”, “OAD only”, “Both insulin and other glucose-lowering medication”, “Any anti-diabetic drugs” and “Number of anti-diabetic medication types” to reduce collinearities.

§ If at least one medication group was listed as a potentially relevant predictor in the literature, we included all other medication groups at the same level as separate variables even if they had not previously been mentioned in the literature.

Additional File 18. Potential medication related predictors based on previous models: Antithrombotic drugs (Anticoagulants) *

Variable	Variable levels	Definition (ATC Codes)	Reference
**Antithrombotic drugs (Anticoagulants)**			
Any antithrombotic drugs	0, 1	B01A	(48, 50)
**Specific antithrombotic medication groups †**			
Number of specific antithrombotic medication groups	0-7	B01AA, B01AB B01AC, B01AD, B01AE, B01AF, B01AX	
Any Vitamin K antagonists	0, 1	B01AA	
Any Heparin group	0, 1	B01AB	
Any Platelet aggregation inhibitors excl. heparin	0, 1	B01AC	(48)
Any antithrombotic Enzymes	0, 1	B01AD	
Any Direct thrombin inhibitors	0, 1	B01AE	
Any Direct factor Xa inhibitors	0, 1	B01AF	
Any Other antithrombotic agents	0, 1	B01AX	
**Specific antithrombotic active ingredients ‡**			
Warfarin	0, 1	B01AA03	(8, 36)
Acetylsalicylic acid (aspirin)	0, 1	B01AC06	(36, 48, 51)

* Listed variables were derived from international literature in combination with the WHO ATC catalogue without considering the current availability or prescription frequencies of specific drugs/drug classes in Germany. Certain drugs/drug classes may therefore not be prescribed to any patient in the data set.

† If at least one medication group was listed as a potentially relevant predictor in the literature, we included all other medication groups at the same level as separate variables even if they had not previously been mentioned in the literature.

‡ At the level of active ingredients, only those explicitly mentioned separately in the literature were used to derive variables.

Additional File 19. Potential medication related predictors based on previous models: Anti-hypertensive drugs *

Variable	Variable levels	Definition (ATC Codes)	Reference
**Anti-hypertensive drugs**			
Any anti-hypertensive drug	0, 1	C02, C03, C07, C08, C09	(25, 26, 31, 32, 35, 48, 49)
Number of anti-hypertensive drug categories	0-5	C02, C03, C07, C08, C09	(50)
**Specific anti-hypertensive medication categories**			
Any antihypertensives	0, 1	C02	
Any diuretics	0, 1	C03	(51)
Any beta-blocking agents	0, 1	C07	(8, 51)
Any calcium channel blockers	0, 1	C08	(8, 51)
Any agents acting on the renin-angiotensin system	0, 1	C09	(8, 36)
**Specific anti-hypertensive medication types †**			
Any antiadrenergic agents, peripherally acting (alpha blockers)	0, 1	C02C	(8)
Any low ceiling diuretic, thiazides	0, 1	C03A	(8)
Any high ceiling diuretic (loop diuretics)	0, 1	C03C	(8)
Any aldosterone antagonist and other potassium-sparing agent	0, 1	C03D	(8)
Any ACE inhibitor, plain or combinations	0, 1	C09A, C09B	(8, 24, 26)
Any angiotensin II receptor blocker (ARB), plain or combinations	0, 1	C09C, C09D	(8, 24, 26, 51)
Any other agents acting on the renin-angiotensin system	0, 1	C09X	(24, 36)

* Listed variables were derived from international literature in combination with the WHO ATC catalogue without considering the current availability or prescription frequencies of specific drugs/drug classes in Germany. Certain drugs/drug classes may therefore not be prescribed to any patient in the data set.

† At the level of medication types, only those explicitly mentioned separately in the literature were used to derive variables.

Additional File 20. Potential medication related predictors based on previous models: Lipid modifying agents *

Variable	Variable levels	Definition (ATC Codes)	Reference
**Lipid modifying agents †**			
Any lipid modifying agents	0, 1	C10	(25, 26, 36)
**Specific lipid modifying medication types**			
Any lipid modifying agents, plain	0, 1	C10A	
Any lipid modifying agents, combinations	0, 1	C10B	
**Specific lipid modifying medication groups ‡**			
Any HMG CoA reductase inhibitors (statins)	0, 1	C10AA	(8, 48, 49, 51)
Any Fibrates	0, 1	C10AB	(8, 48)
Any Bile acid sequestrants	0, 1	C10AC	(8)
Any Other lipid lowering medication group	0, 1	C10AX	(51)
**Specific anti-hypertensive active ingredients §**			
Ezetimibe	0, 1	C10AX09	(8)

* Listed variables were derived from international literature in combination with the WHO ATC catalogue without considering the current availability or prescription frequencies of specific drugs/drug classes in Germany. Certain drugs/drug classes may therefore not be prescribed to any patient in the data set.
† Variable "Any Niacin (nicotinic acid and derivatives) (C10AD)"(8) was removed from the final data set because the respective medication was never prescribed
‡ If at least one medication group was listed as a potentially relevant predictor in the literature, we included all other medication groups at the same level as separate variables even if they had not previously been mentioned in the literature.
§ At the level of active ingredients, only those explicitly mentioned separately in the literature were used to derive variables.

Additional File 21. Potential medication related predictors based on previous models: Cardiac Medications *

Variable	Variable levels	Definition (ATC Codes)	Reference
**Cardiac Medications**			
Any cardiac therapy drugs	0, 1	C01	
**Specific cardiac medication types** †			
Cardiac glycosides	0, 1	C01A	
Anti-arrhythmic class I and III	0, 1	C01B	(8)
**Specific cardiac medications active ingredients** ‡			
Digoxin	0, 1	C01AA05	(8)

* Listed variables were derived from international literature in combination with the WHO ATC catalogue without considering the current availability or prescription frequencies of specific drugs/drug classes in Germany. Certain drugs/drug classes may therefore not be prescribed to any patient in the data set.
† If an active ingredient from a specific medication type was identified as potentially relevant predictor in the literature, the type was also included as a separate variable
‡ At the level of active ingredients, only those explicitly mentioned separately in the literature were used to derive variables.

Additional File 22. Potential medication related predictors based on previous models: CNS-related drugs *

Variable	Variable levels	Definition (ATC Codes)	Reference
**CNS drug categories**			
Any psychoanaleptics	0, 1	N06	
Any psycholeptics	0, 1	N05	
Any antiepileptics (anti-seizure)	0, 1	N03	(8)
Any analgesics	0, 1	N02	
**Specific CNS medication types †**			
Antidepressants	0, 1	N06A	(8)
Dementia drugs	0, 1	N06D	
Antipsychotic (neuroleptics)	0, 1	N05A	(8)
Anxiolytics	0, 1	N05B	
Hypnotics and sedatives	0, 1	N05C	
Opioids	0, 1	N02A	
**Specific CNS medication groups †**			
Anticholinesterases	0, 1	N06DA OR N07AA	(8)
Other anti-dementia drugs	0, 1	N06DX	
Benziodiazepine derivatives	0, 1	N05BA OR N05CD OR N05CF OR N03AE	(8)
**Specific CNS medications active ingredients ‡**			
Memantine	0, 1	N06DX01	(8)
Tramadol	0, 1	N02AX02	(8)

* Listed variables were derived from international literature in combination with the WHO ATC catalogue without considering the current availability or prescription frequencies of specific drugs/drug classes in Germany. Certain drugs/drug classes may therefore not be prescribed to any patient in the data set.

† If an active ingredient from a specific medication type or medication group was identified as potentially relevant predictor in the literature, the type or group was also included as a separate variable

‡ At the level of active ingredients, only those explicitly mentioned separately in the literature were used to derive variables.

Additional File 23. Potential medication related predictors based on previous models: Other drugs *

Variable	Variable levels	Definition (ATC Codes)	Reference
**Other medication categories †**			
Antianemic drugs	0, 1	B03	
Endocrine therapy	0, 1	L02	
Corticosteroids for systemic use	0, 1	H02	(8)
Thyroid therapy	0, 1	H03	
Cough and cold preparations	0, 1	R05	
Antibacterials for systemic use	0, 1	J01	
Vitamins	0, 1	A11	
Anti-inflammatory and anti-rheumatic products	0, 1	M01	
**Other medication types**			
Quinolone antibacterials	0, 1	J01M	(8)
Non-steroidal anti-inflammatory medications	0, 1	M01A	(48)
**Specific other medication groups**			
Estrogen agonists (anti estrogens)	0, 1	L02BA	(8)
**Specific other active ingredients**			
Epoetin (Erythropoietin, darbepoetin alfa, methoxy polyethylene glycol-epoetin beta)	0,1	B03XA01 OR B03XA02 OR B03XA03	(8)
Levothyroxine	0, 1	H03AA01	(8)
Codeine	0, 1	R05DA04	(8)
Calcitriol	0, 1	A11CC04	(8)

* Listed variables were derived from international literature in combination with the WHO ATC catalogue without considering the current availability or prescription frequencies of specific drugs/drug classes in Germany. Certain drugs/drug classes may therefore not be prescribed to any patient in the data set.
† If an active ingredient, medication group or medication type from a specific medication category was identified as potentially relevant predictor in the literature, the category was also included as a separate variable

Additional File 24. Feature engineering – technical details
Continuous features were standardized to a mean of 0 and a standard deviation of 1. Categorical features were recategorized, where necessary, to avoid computational challenges through sufficient class occupations, and subsequently dummy coded for all except the two deep learning models, which used one-hot encoding. Extreme values were kept, because exclusion of outliers may lead to biased predictive model performance if occurrence is not completely random.(4)

Additional File 25. Time windows used for prediction modeling

**Y**	**2014**				**2015**				**2016**				**2017**				**2018**				**2019**		
**Q**	1	2	3	4	5	6	7	8	9	10	11	12	13	14	15	16	17	18	19	20	21	22	23
					**O**	**O**	**O**	**O**	**B**	**B**	**T**	**T**	**T**	**T**	**T**	**T**	**T**	**T**	**T**	**T**	**T**	**T**	

Additional File 25. Time windows used for prediction modeling.
Legend: Y: Calendar years available for this project. Q: Quarters available for this project, numbered ascending in chronological sequence. O: One-year Observation period – used to apply eligibility criteria and measure potentially predictive features, with which the algorithms were trained. B: 6-month Buffer period – not used for analysis. T: Three-year Target period – used for measurement of outcomes, which the algorithms were trained to predict.

Additional File 26. Software and program codes
Data management and preparation was executed in *SAS 9.4.(52)* Training of the models using regression-based and tree-based algorithms was conducted in *RStudio 2022.02.3 (53)* using the *tidymodels*(54) package. Training of the deep learning algorithms was conducted in *Python 3.9*(55) using the libraries *Pytorch*(56) and *Tensorflow*,(57) with *Pytorch-Lightning*,(58) *Pytorch-Tabular*,(59, 60) and *Tensorboard*(57) extensions. A data dictionary defining the variables in the original datasets and the final derived dataset as well as the programs used for data management, model development, and model evaluation are publicly available at the OpenScienceFramework (OSF) under the following link: https://osf.io/v2h7d.

Additional File 27. Model development – Technical details
We trained three logistic regression models (a full model using all features in the dataset, forward selection, and regularization through Least Absolute Shrinkage and Selection Operator (LASSO)), and two models based on tree-based machine learning (random forests, gradient boosting) and ~~two~~ deep learning algorithms (multi-layer perceptron, feature tokenizer transformer), respectively, to identify diabetes patients with stroke or myocardial infarction events in the target period.
While the first two logistic regression approaches are relatively simple to implement with comparatively low computational effort, they can only deal with limited numbers of predictors, as they are prone to variance inflation and inflated coefficient size introduced by collinearities between predictors. LASSO overcomes the issue of inflated coefficient sizes by penalizing the sum of the regression coefficients' absolute values. All logistic models imply a linear predictor, making consideration of potential interactions between predictors tedious, as they need to be explicitly foreseen and introduced by the modeler. This challenge is overcome by tree-based methods which split the data consecutively based on the most predictive variable available for the respective data splitting step. Thus, each split in the tree represents an additional data-driven identification of relevant interactions between the predictor selected for the respective split and all predictors selected for preceding data splits. While random forests average over multiple independent trees, gradient boosting can improve efficiency by building each tree specifically with the objective to reduce the residual unexplained variance remaining from all previous tree building processes. Simply put, deep learning methods can be seen as trees of logistic models, adding a probabilistic component to tree-based methods. This further reduces explainability and increases requirements regarding computational resources and dataset size, but can lead to improved predictions, especially in fields like Natural Language Processing or Image Recognition.
Within the training set, we used 10-fold cross-validation with withhold-out data sets for parameter tuning in the machine learning and LASSO models. For the deep learning models, we used 75% of the training set for training a learner and dealing with early stopping to avoid overfitting and 25% of the training set for hyperparameter tuning. In addition to the abovementioned models, we created a null model without any predictors and therefore without any explanatory value for comparison.

To ensure comparability of model performance indicators between the logistic models and more sophisticated models for each outcome, the same set of variables was offered to all employed variable selection algorithms without any pre-selection.

Null model: Model without predictors

The null model was implemented as a logistic model without any predictors using the *glm* engine in *tidymodels*.

Logistic regression models

Two logistic regression models were fitted to the data set using a forward selection method and the full set of potential features, respectively. Since *tidymodels*(54) does not support forward selection, feature selection was implemented through the *stepAIC* function in the *MASS* package(61) and the resulting list of selected features included in the *tidymodels* recipe. Both the full model and the one with those forward selected predictors resulting in the lowest Akaike Information Criterion were then fitted using the *glm* engine in *tidymodels*. Model-specific relative variable importance was assessed via the *vi* function in the *vip* package(62) based on the size of the beta coefficients.

Regularization

The LASSO method is characterized by a penalty term (or shrinkage parameter), which reduces estimated coefficient size. Depending on the size of the shrinkage parameter, estimated coefficients of the less relevant variables can be shrunk to zero, allowing for automated feature selection. LASSO models were fitted using the *glmnet* engine in *tidymodels*. Ten-fold cross validation was used to identify the optimal shrinkage parameter with regard to training AUPRC based on grid searches with 100 different shrinkage parameters each. The initial grid was created as a regular grid with penalty terms ranging between 10^{-10} and 1. Tuning was repeated multiple times with adapted grids based on the results of the respective last tuning round and parallelized across folds using the *doParallel* package. After each tuning round, the average training AUPRC of each of the 100 tuned models based on the hold-out data sets from 10-fold cross validation in the training set was plotted against the size of the penalty term. Visual inspection of this plot guided the redefinition of the grid ranges for the next round of tuning. Grids were additionally adapted to result in models with at least one non-zero coefficient. The decision to stop tuning was made once changes in the maximum AUPRC differences, mean AUPRC and maximum AUPRC between the tuned models between tuning rounds became minimal or no further trends could be visually identified based on the AUPRC performance plots. As for the logistic models, model-specific relative variable importance was assessed via the *vi* function in the *vip* package based on the size of the beta coefficients.

Random Forests

Random forests for stroke or myocardial infarction consisted of multiple bootstrap-aggregated (bagged) classification trees. In this method, each tree is built based on a bootstrapped sample of observations. Tree building occurs in a greedy process which stratifies the predictor space in distinct regions, at each node using a random sample of all potential predictors p. At each node, the process selects the split which leads to the largest reduction in a pre-specified performance metric (in our case training AUPRC). Trees are subsequently aggregated by averaging predictions for each observation across all trees.

Random Forests were fitted using the *ranger* engine in *tidymodels*. Split points were chosen based on the Hellinger Distance instead of the pre-specified Gini Index, as Hellinger Distance gives high scores to a split separating the classes in the best way relative to the parent population instead of favoring splits which result in an uneven class distribution (as in the case of Gini Index as split criterion, for example). Therefore, we considered Hellinger Distance more adequate in our case with highly imbalanced outcome data.(63)

We tuned the number of trees in the forest, the number of random candidate features evaluated at each split, the minimal node size required to execute another split, and the size of the bootstrapped data set (fraction of the original sample without replacement) using the internal parallelization option *num.threads* in *tidymodels*.

Initial ranges for each tuning parameter (number of trees: 1-2000, number of random candidate features evaluated: 1-p, minimal node size: 2-40, sample fraction: 0.1-1.0) were chosen based on default values of the *ranger* package and literature.(64). The final set of values was selected by systematically varying each of the values in order to identify the combination which results in the best training AUPRC.(65) This was implemented through iterative grid searches with 20 different combinations of these parameters selected based on latin hypercube sampling, a space-filling design that aims to optimally cover the hyperparameter space with a grid. After each tuning round, the average training AUPRC of each of the 20 tuned models based on the hold-out data sets from 10-fold cross validation in the training set was plotted against each of the tuning parameters. Visual inspection of these plots guided the redefinition of each parameter's grid ranges for the next round of tuning. Where no clear pattern was recognized in these plots for a specific tuning parameter, the grid range from the last tuning round was kept for the next round. The decision to stop tuning was made once changes in the maximum AUPRC differences, mean

AUPRC and maximum AUPRC between the tuned models between tuning rounds became minimal or no further trends could be visually identified based on the AUPRC performance plots.
Model-specific relative variable importance was assessed via the *vi* function in the *vip* package which used the *importance="impurity"* option specified in *ranger* by averaging each variable's tree-specific contribution to the decrease in node impurity across all trees in the random forest. Impurity is measured by the Gini index.

Gradient Boosting

Gradient boosted forests consist of an additive function of multiple sequentially grown and subsequently shrunken classification trees built on the residuals of the function comprising all previously built trees. Gradient boosted forests were fitted using the *xgboost* engine in *tidymodels*. We tuned the number of trees in the forest, the maximum tree depth, the number of random candidate features evaluated at each split, the minimal node size and the minimum loss reduction required to execute another split, the size of the shrinkage parameter ("learn rate") and the size of the bootstrapped data set (fraction of the original sample without replacement), using the internal parallelization option *num.threads* in *tidymodels*.
Initial ranges for each tuning parameter (number of trees: 1-2000, maximum tree depth: 1-15, number of random candidate features evaluated: 1-*p*, minimal node size: 2-40, minimum loss reduction: 10^{-10}-$10^{1.5}$, learn rate: 10^{-3}-$10^{-0.5}$, sample fraction: 0.1-1.0,) were chosen based on default values of the *ranger* package. The final set of values was selected analogously to the tuning procedure described for random forests.
Model-specific relative variable importance was assessed via the default *vi_model* option (*type = "gain"*) in the vi function of the *vip* package as the fractional contribution of each feature to the model based on the total gain of the corresponding feature's splits.

Multi-Layer Perceptron

A Multi-Layer Perceptron (MLP) consists of three types of node layers: one input layer, one or more hidden layers, and one output layer. Except for the input nodes, each node is a neuron with a nonlinear activation function (66). We applied learning via Stochastic Gradient Descent (SGD), which searches for a local minimum to avoid issues related to collinearity among the covariates. Furthermore, we used a weighted binary cross entropy loss function (in which the contribution of each observation to the loss is weighted by the inverse of its class frequency), and combined it with random over- and undersampling during the training to address the imbalance of the data set. In addition to the first 80%:20% split into training and test sets that was identical for all models, for the deep learning models, 25% of the training data were sub-sampled for hyperparameter tuning. Initial training and dealing with the early stopping problem to avoid overfitting was therefore only done on the remaining 75% of the original training data set. After hyperparameter tuning, the best model was retrained on the whole training data and subsequently evaluated on the original test data set that was again identical for all models. In sum, a 60 : 20 : 20 split in was applied to the data for training (combined with early stopping), hyperparameter tuning, and validation of the deep learning models, respectively.
In a first step, models with between 2 and 15 hidden layers were initially compared in terms of validation sample AUPRC results within the training set, while holding the dimensions of the hidden layers (2^{10}), learning rate (0.001), batch size (128), and the number of epochs (20) constant. Where multiple models performed equally well, the model with the lower number of hidden layers was chosen. Subsequently, a grid search in the range 2^{10} until 2^{14} was performed for the dimensions of the layers. Where multiple models performed equally well, the one with the smoothest loss curve was chosen. Then, we tested if addition of hidden layers to create a diamond shape improved the validation sample AUPCR within the training set. We used an algorithm which finds an optimal learning rate before starting the actual training and implemented an early stopping algorithm, which prevents overfitting by checking whether the validation loss has increased for more than three epochs in a row and ends the training accordingly. Additionally, we fine-tuned dropout rates and the batch size.
As the oversampling of observations with and undersampling of observations without events that we used during training of the MLP to reach a balanced event occurrence of 50% produced overconfident probability estimates, we used histogram binning to obtain interpretable output probabilities. In histogram binning, both the observed and the predicted probability distribution are divided into bins and a scaling function is applied to each predicted probability within a bin to project it onto the respective observed bin, based on linear interpolation between bin means. Variable importance was not estimated.

Feature Tokenizer Transformer

The Feature Tokenizer Transformer is a neural network architecture designed for tabular data applications, consisting of two main components. The first component, the Feature Tokenizer Layer, is responsible for transforming the tabular data into a suitable input format for the Transformer algorithm. This layer maps each input feature to a continuous vector representation and generates an embedded input tensor that can be fed into the second component of the architecture. The second component is the Transformer Layer, which takes the embedded

input tensor generated by the Feature Tokenizer Layer and applies the Transformer architecture to calculate predictions. The Transformer Layer utilizes multihead self-attention and feedforward neural networks to model the relationships between input features and generate predictions. As in the MLP, also for the FTT the training sample was further subdivided into 75% initial training and 25% hyperparameter tuning data sets, and we again used a weighted binary cross entropy loss function combined with random over- and undersampling for training. In the Feed Forward Network of each Transformer layer, we systematically identified the adequate dimensionality and number of hidden layers similarly to the process described for the MLP architecture. For every multi-head self-attention layer, we stacked eight attention heads (h = 8) in each layer. Subsequently, we fine-tuned the learning rate, the dropout within the feedforward network and the batch size. To map the output of the Transformer layers to the desired output dimension, we used a linear classification head, which consists of a rectified linear activation function and a linear layer. Analogously to the MLP, histogram binning was used to obtain interpretable output probabilities. Variable importance was not estimated.

Additional File 28. Details on parameter tuning

Models without hyperparameter tuning
Forward selection resulted in logistic models with 89 and 78 predictors for MI and stroke, respectively. The full logistic models did not provide estimates for 9 out of 324 regression coefficients due to collinearity issues.

Models with hyperparameter tuning
For MI models, tuning for LASSO, random forests and gradient boosting was ended after 5, 5 and 8 rounds of grid search, and for stroke models after 5, 7 and 5 rounds of grid search, respectively. The Deep Learning models, Multi-Layer Perceptron and Feature Tokenizer Transformer, were trained for 20 epochs in the MLP for both outcomes and for 15 (MI) and 16 (stroke) epochs in the FTT using Stochastic Gradient Descent. After the respective number of epochs, the early stopping mechanism took effect to avoid overfitting. **S29 Table** provides a summary of initial ranges and final values for tuning parameters in each tuned model.

Additional File 29. Initial ranges and final values for tuning parameters

	**Tuning Parameter**	**Initial range**	**Final value**	
**Model**			**MI**	**Stroke**
LASSO	penalty term	$[10^{-10}, 1]$	0.000264856	0.000248362
Random Forest	number of trees	[1, 2000]	1102	1513
	number of features	$[1, p]$	25	10
	minimal node size	[2, 40]	21	25
	sample size fraction	[0.1, 1.0]	0.0885	0.372
Gradient Boosting	number of trees	[1, 2000]	1131	1363
	number of features	$[1, p]$	46	90
	maximum tree depth	[1, 15]	6	3
	minimal node size	[2, 40]	9	19
	sample size fraction	[0.1, 1.0]	0.250	0.393
	loss reduction	$[10^{-10}, 10^{1.5}]$	0.000000592	0.0000000154
	learning rate	$[10^{-3}, 10^{-0.5}]$	0.00948	0.00967
Multi-Layer Perceptron	number of hidden layers	[2, 15]	2	2
	layer dimensions	$[2^0,2^{10}]$	213-213	213-213
	diamond shape	[yes, no]	no	no
	learning rate	[0.00001, 0.01]	0.00015	0.00015
	batch size	[128, 2048]	2048	2048
	number of epochs	[0, 100]	20	20
	drop-out rates	[0, 0.25]	0.1	0.1
Feature Tokenizer Transformer	number of hidden layers	[2, 6]	3	3
	layer dimensions	$[2^0, 2^{10}]$	128−64−28	128−64−28
	learning rate	[0.00001, 0.01]	0.0001	0.0001
	batch size	[64, 512]	256	128
	number of epochs	[0, 100]	15	16
	drop-out rates	[0, 0.25]	0.1	0.1
	number of attention heads	8	8	8

Additional File 30. Overview and definition of discrimination metrics

Discrimination describes the ability to differentiate between individuals who will develop the outcome and those that will not. A good discriminative model therefore predicts on average higher event probabilities for individuals who will develop the outcome of interest and lower event probabilities for individuals who will not develop the outcome.(67) For a model with perfect discriminative ability, all predicted probabilities for individuals who will not develop the outcome of interest would be lower than the lowest probability that is predicted by the model for an individual who will develop the outcome.

Area Under the Receiver-Operator Characteristic (AUROC) curve

The Area Under the Receiver-Operator Characteristic curve (AUROC, concordance, c-statistic) is the most widely used discrimination metric. It plots, for each potential classification threshold (i.e. each probability output value between 0 and 1, that could be used to mark the cut-off to differentiate between future cases and non-cases), a model's respective true positive rate (sensitivity, see definition further below) against the probability of incorrectly classifying a non-case as a predicted case (false positives, 1 minus specificity, see definition further below).(68, 69) In other words, AUROC values reflect the probability θ that the model's predicted event probabilities from a randomly selected pair of observations *i* and *j* from the cases *m* and non-cases *n*, where one will develop the outcome of interest and the other will not, are correctly ordered, i.e. that the predicted risk X of the observation *i* that will develop the outcome is higher than the predicted risk Y of the observation *j* that will not develop the outcome (see formula below).(70, 71)

$$\boldsymbol{\theta} = \frac{\mathbf{1}}{\boldsymbol{mn}}\sum_{\boldsymbol{i}=\mathbf{1}}^{\boldsymbol{m}}\sum_{\boldsymbol{j}=\mathbf{1}}^{\boldsymbol{n}}\boldsymbol{v}(\boldsymbol{X_i}-\boldsymbol{Y_j})$$

$$v(X_i - Y_j) = \begin{cases} 1 & Y < X \\ 0.5 & Y = X \\ 0 & Y > X \end{cases}$$

ROC curves are by definition necessarily monotonically increasing with all potential thresholds ordered in descending sequence:(70) The bottom left coordinate reflects the threshold probability of 1, with all observations classified as non-cases, and the top right coordinate reflects the threshold probability of 0, with all observations classified as cases.

With continued threshold decreases starting from 1 and thereby moving along the ROC curve, at a certain point the first single individual with the highest predicted probability is classified as a predicted future case. If this individual is actually a future case (i.e. a true positive), sensitivity increases by the fraction that this individual represents among all future cases. If the two individuals with the next highest predicted probability are actually future non-cases (i.e. false positives), the sensitivity remains the same, but specificity starts decreasing, i.e. the curve starts moving rightwards along the x-axis, as each new individual that is classified wrongly as a future case increases the share of false positives, or, decreases the share of future non-cases that can correctly be classified as such. Therefore, with slowly decreasing threshold values, the curve starts stretching rightwards along the x-axis and upwards along the y-axis, until, at the right end of the x-axis and the top end of the y-axis, all individuals' predicted probabilities (both of future cases and non-cases) are above the threshold value.

For a model with perfect discriminative ability, the ROC curve would pass through the top left coordinate ((0,1) in the case when x- and y-axes are labeled "sensitivity" and "1-specificity").

Independently of disease prevalence,(70) the AUROC does put equal emphasis on the model's capability to correctly identify true positives via the sensitivity component and the model's capability to correctly identify true negatives via the specificity component. This makes it a suitable metric for balanced data sets where the number of negative cases is approximately equal to the number of positive cases and it is equally important to detect true positive and true negative cases.

AUROC values ranges from 0.5 to 1, where a perfect AUROC of 1 corresponds to a model that detects all positive cases without marking any negative cases as positive. An AUROC with no informative value ("no skill") is equal to 0.5, representing a scenario where true positive cases have on average the same predicted event risk as the true negative cases.

In our study, confidence intervals for the area under the receiver operating curves (AUROC) were bootstrapped using the *ci.auc* function in the *pROC* package.(72)

Area Under the Precision-Recall Curve (AUPRC)

The Area Under the Precision-Recall Curve (AUPRC) is a discrimination metric suggested to be suitable specifically for imbalanced classification tasks(70) as it focuses on the ability of the prediction model to identify future cases and ignores correctly classified future non-cases (true negatives), i.e. the prevailing group in the case

of a low-prevalence condition.(70) It plots, for each potential classification threshold across the full range of predicted probabilities,(4) a model's respective precision (positive predictive value, see definition further below) against its recall (sensitivity, true positive rate, see definition further below). The bottom left coordinate reflects the threshold probability of 1, with all observations classified as non-cases, and the bottom right coordinate reflects the threshold probability of 0, with all observations classified as cases.

The PR curve is not necessarily monotonic across all thresholds because a decrease in the threshold can increase both the true positive and the false positive rates.(70) This is most visible in the very left part of the figure, where with continued threshold decreases starting from 1, at a certain point the first single individual with the highest predicted probability is classified as a predicted future case. If this individual is actually a future case (i.e. a true positive), the PR curve jumps to a positive predictive value of 1 as soon as the threshold is lowered below the respective individuals' predicted probability. If the two individuals with the next highest predicted probability are actually future non-cases (i.e. false positives), the positive predictive value falls to 0.3. If the individuals with the fourth highest predicted probability is again a true positive, the positive predictive value will again jump to 0.5, and so on. At the same time, with slowly decreasing threshold values, the curve starts stretching along the x-axis depending on the share of correctly identified future cases (i.e. the true positives), until, at the right end of the x-axis, all actual future cases are above the threshold value. The closer the PR curve gets to the top right corner (i.e. to 100% precision and 100% recall), the better the model performance.(69)

As the AUPRC does not account for true negatives, it is a suitable metric for imbalanced data sets where the number of negative samples dominates the number of positive samples and it is specifically important to detect rare positive cases, such as in fraud detection or medical diagnosis.

The AUPRC ranges from the positive ratio (case prevalence, in our case, approximately 3.5% or 0.035, equivalent to the positive predictive values at full sensitivity, i.e. when the probability threshold is 0 and everyone is classified as a case) in the data set to 1 for a perfectly discriminative model.

The AUPRC can be calculated in different ways.(73) For this project, we chose the Average Precision (AP) estimator. The Average Precision estimator is calculated as the mean of the Precision values achieved at each threshold *k* across all thresholds *l*, where each Precision value is weighted with the increase in Recall from the previous threshold:

$$\boldsymbol{Average\ Precision} = \sum_{\boldsymbol{k=1}}^{\boldsymbol{l}} (\boldsymbol{R_k} - \boldsymbol{R_{k-1}}) * \boldsymbol{P_k}$$

P_k = precision at the k-th threshold
R_k = recall at the k-th threshold

The AUPRC was reported with bootstrapped confidence intervals based on 2000 replications.

Additional File 31. Overview and definition of calibration metrics

Calibration describes the agreement between predicted probabilities and observed event rates. Models that perform well in terms of discrimination may still considerably under- or overestimate individual risk, with undesirable consequences in practice if treatment decisions are based on the absolute value of predicted risk estimates.(74) Generally, observed over/under-estimation of risks affects the clinical usefulness of a prediction model, because in clinical practice both patients and their treating physicians weigh the predicted probability of event occurrence against the risks and costs of potential countermeasures. For example, higher risks may be deemed acceptable in order to avoid an adverse event that is predicted to occur with a high probability (e.g. 90%) compared to trying to prevent an adverse event that is predicted to occur with a lower probability (e.g. 10%). So-called mean, weak, and moderate calibration was therefore assessed to understand the accuracy of our models' predicted probabilities.(75)

Mean calibration

Mean calibration, also sometimes called "calibration-in-the-large" compares the average predicted risk with the observed overall event rate. It is calculated by subtracting the average predicted risk from the observed overall event rate. The resulting values can range between -1 and 1, with perfect calibration at a value of zero. Positive values indicate a tendency to underestimate average risk, while negative values indicate a tendency to overestimate average risk.

Similarly, mean calibration can be assessed by the intercept in a logistic regression of the individual-level dichotomous variable "observed event yes or no" against each individuals' predicted log-odds of experiencing the event. Also in this case, negative calibration intercepts suggest risk overestimation, while positive calibration intercepts suggest risk underestimation, with an optimal target value of zero.

Weak calibration

Weak calibration indicates how much the spread of the estimated risks deviates from its target value of 1. It can be assessed by the slope in a logistic regression of the individual-level dichotomous variable "observed event yes or no" against each individuals' predicted log-odds of experiencing the event. Calibration slopes <1 suggest that estimated risks are too extreme, i.e. a model's tendency to underestimate low risks and/or overestimate high risks. In contrast, calibration slopes >1 suggest that risk estimates are too moderate. i.e. a model's tendency to overestimate low risks and/or underestimate high risks.(74)

Moderate calibration

Moderate calibration can be assessed visually by inspecting the relationship between predicted and observed values using calibration plots. These can be output as flexible curves to show the relation between the mid-point in specified predicted risk bins (on the x-axis) and the observed proportion of events in those bins (y-axis), for example, using local regression (loess).(75) In our case, we used bins of the size 0.01 for predicted probabilities between zero and 0.1 and bins of the size 0.05 for predicted probabilities between 0.1 and 1.

Perfect moderate calibration occurs in a situation where estimated risks correspond to observed proportions in all specified bins of predicted probabilities, e.g., when among observations in a bin with an estimated risk mid-point of 10%, 10 in 100 actually develop the event. This would result in a curve along the diagonal. Curves running below this diagonal indicate a tendency to overestimate risks, whereas curves running above this diagonal indicate a tendency to underestimate risks.

The visual inspection of calibration plots additionally helps understand the range of risk predictions output by the model.

Additional File 32. Overview and definition of classification metrics

Once a specific cut-off within the range of a model's predicted probabilities has been chosen to classify new observations as either predicted cases or predicted non-cases, classification performance (i.e. the degree to which individuals are correctly classified) at this specific threshold can be evaluated with various metrics.

Sensitivity (Recall, True Positive Rate)

Sensitivity is sometimes also called "Recall" or "True Positive Rate". It is relevant when the model's ability to correctly predict individuals with upcoming events is compared to competing tools for clinical application. It represents the proportion of cases that a model correctly predicts out of the total number of actual cases.(69) Sensitivity values range between 0 and 1, with higher sensitivity indicating that the model does classify less cases erroneously as non-cases. A sensitivity of 0 is equal to a model that deterministically classifies everyone as a non-case.

$$\boldsymbol{Sensitivity = \frac{TP}{TP + FN}}$$

TP = number of true positives
FN = number of false negatives

Specificity

Specificity is relevant in cases when the model's ability to correctly predict individuals without upcoming events is compared to competing tools for clinical application. It represents the proportion of non-cases that a model correctly predicts out of the total number of individuals without a future event.(69) Specificity values range between 0 and 1, with higher specificity indicating that the model does classify less non-cases erroneously as cases. A specificity of 0 is equal to a model that classifies everyone deterministically as a case.
Specificity may be less relevant in contexts when the focus is on identification of the positive class, especially when this positive class is the minority class.

$$\boldsymbol{Specificity = \frac{TN}{TN + FP}}$$

TN = number of true negatives
FP = number of false positives

Precision (Positive Predictive Value, PPV)

Positive Predictive Values (PPV) are also sometimes called "precision". In contrast to sensitivity and specificity, the PPV depends on the prevalence of the target event and is therefore not an invariant characteristic of the model. Instead, it may change for the same model depending on the population and context in which the model is applied.(76)
The PPV represents the probability that an event actually occurs in an individual with a predicted event. It ranges from 0 to 1 and is calculated as the proportion of actual cases divided by all observations predicted to become a case.(69)

$$\boldsymbol{Precision = \frac{TP}{TP + FP} = \frac{sensitivity * prevalence}{sensitivity * prevalence + (1 - sensitivity) * (1 - prevalence)}}$$

TP = number of true positives
FP = number of false positives

Negative Predictive Value (NPV)

The proportion of actual non-cases divided by all observations predicted not to become a case. It ranges from 0 to 1. Because the NPV depends, just like the PPV, on the prevalence of the target event, NPV is typically high in cases when the outcome is rare.(69) In such situations, a high NPV may misleadingly suggest good model performance even if the predictive performance for the positive class is low.

$$\boldsymbol{Negative\ Predictive\ Value = \frac{TN}{TN + FN}}$$

TN = number of true negatives
FN = number of false negatives

Positive Likelihood Ratio (PLR)
The Positive Likelihood Ratio is the factor, by which an actual case is more likely to receive a prediction as future case from the model compared to a non-case.(76) Like the PPV, the PLR is a patient-relevant metric indicating how much a concrete positive screening result can be trusted. In contrast to the PPV, PLRs are independent of the prevalence. For a model which cannot differentiate between cases and non-cases, the PLR is equal to 1. The more the PLR exceeds 1, the better its predictive performance.(69) It is calculated as the probability that a person who becomes an actual case is predicted as future case by the model, divided by the probability that an actual non-case is predicted to become a future case.

$$\boldsymbol{Positive\ Likelihood\ Ratio\ = \frac{sensitivity}{1 - specificity}}$$

Negative Likelihood Ratios (NLR)
The Negative Likelihood Ratio is the factor, by which an actual case is less likely to receive a prediction as future non-case from the model compared to an actual non-case.(76) Like the NPV, the Negative Likelihood ratio is a patient-relevant metric indicating how much a concrete negative screening result can be trusted. In contrast to the NPV, NLRs are independent of the prevalence. For a model which cannot differentiate between cases and non-cases, the NLR is equal to 1. The more the NLR tends towards zero, the better its predictive performance.(69) It is calculated as the probability that a person who becomes an actual case is predicted as future non-case by the model, divided by the probability that an actual non-case is predicted to become a future non-case.

$$\boldsymbol{Negative\ likelihood\ ratio\ = \frac{1 -\ sensitivity}{specificity}}$$

Number Needed to Evaluate (NNE)
The NNE is the number of individuals that need to be screened with the prediction model to detect one future case. It is calculated as the inverse of the PPV, (e.g. a PPV = 20% is equivalent to a NNE = 5).(69)

$$\boldsymbol{Number\ Needed\ to\ Evaluate\ = \frac{1}{PPV} = \frac{TP + FP}{TP}}$$

TP = number of true positives
FP = number of false positives
PPV = Positive Predictive Value

Alert Rate
The alert rate can be seen as an indicator of resource demands that the models' use for screening purposes would imply.(77) It provides the number of alerts that can be expected per number of patients screened over a specified period of time. A risk of a high alert rate is that it may lead to "alert fatigue" both on the clinician and patient side, and increase the opportunity cost of implementing the model from a health care system perspective.(69)

$$\boldsymbol{Alert\ Rate\ = \frac{TP + FP}{TP + FP + TN + FN}}$$

TP = number of true positives
TN = number of true negatives
FP = number of false positives
FN = number of false negatives

F1-Score
The F1, also known as the F-measure or balanced F-score score, is a weighted average of precision (positive predictive value) and recall (sensitivity). The F1-Score therefore takes both false positives and false negatives into account, which is useful especially when dealing with imbalanced data sets. It ranges from 0 to 1, where 1 represents the best possible score,(69) and can be used to select an optimized classification threshold that balances PPV and sensitivity. In situations where high Precision is perceived as more desirable than high Recall or vice versa, using the F1-Score to select a classification threshold can however lead to a suboptimal classification threshold choice.

$$\boldsymbol{F1\ = 2 * \frac{Precision * Recall}{Precision + Recall}}$$

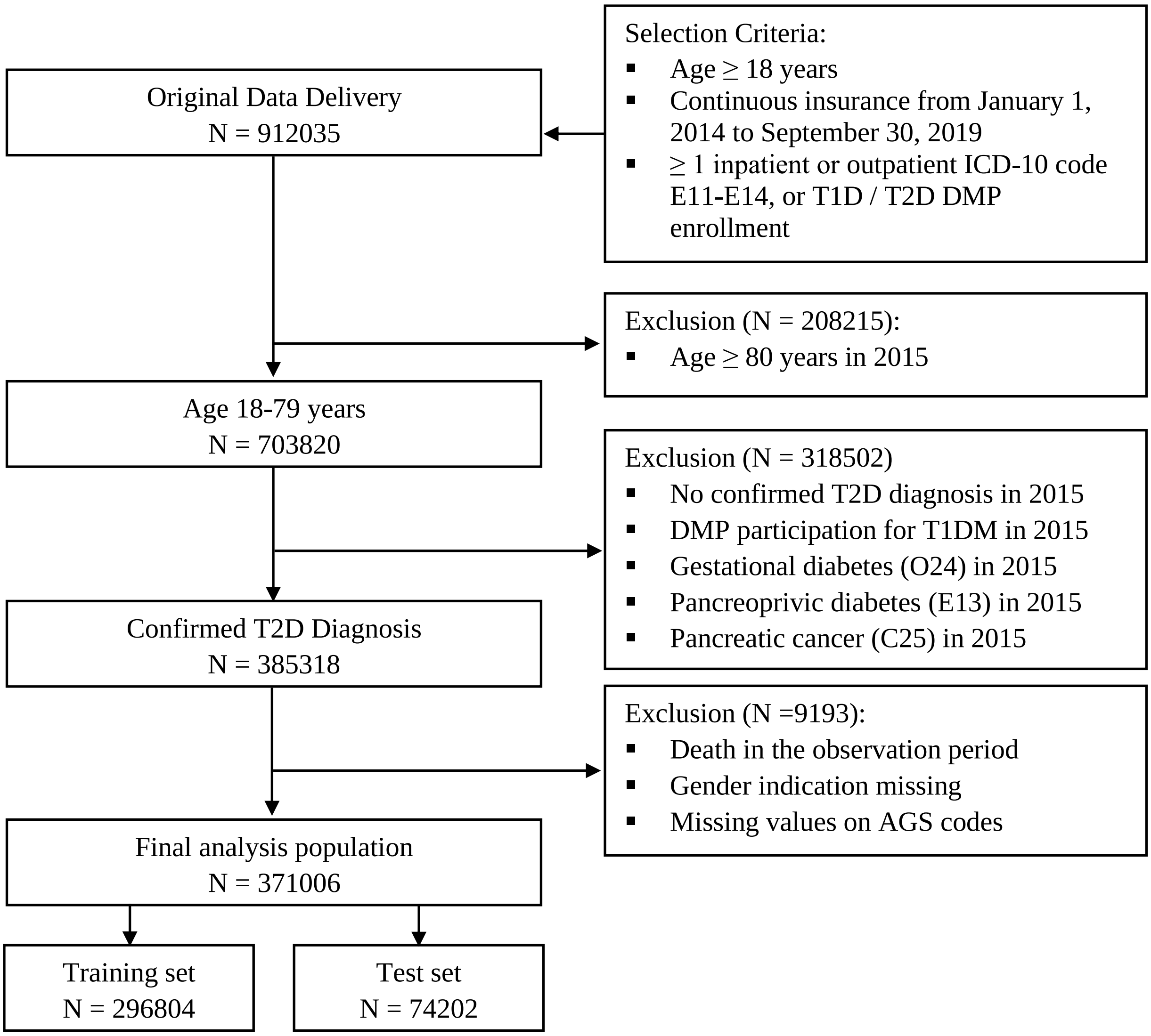

Additional File 33. Participant flow chart.

ICD10: International Classification of Diseases, 10th revision. T1D: Diabetes Mellitus Type 1. T2D: Diabetes Mellitus Type 2. DMP: Disease Management Program.

Additional File 34. Sample characteristics

	Overall		MI in Target Period		Stroke in Target Period	
**Characteristic**	**N**	**(%)**	**N**	**(%)**	**N**	**(%)**
N total (%)	371006	(100)	13030	(100)	12701	(100)
Female	205185	(55.31)	5222	(40.1)	6097	(48.0)
Observation Period (January 2015 - December 2015) – General Characteristics						
Age in years						
Mean, sd	67.2 (9.45)		69.8 (7.87)		70.7 (7.43)	
18-29	665	(0.2)	0	(0)	1	(0.0)
30-39	3643	(1.0)	22	(0.2)	27	(0.2)
40-49	15110	(4.1)	251	(1.9)	166	(1.3)
50-59	53142	(14.3)	1,211	(9.3)	905	(7.1)
60-69	117711	(31.7)	3749	(28.8)	3346	(26.3)
70-79	180735	(48.7)	7797	(59.8)	8256	(65.0)
CCI (mean, sd)	3.06 (2.16)		4.07 (2.49)		3.77 (2.38)	
DMP participation	242058	(65.2)	8384	(64.3)	8118	(63.9)
Newly recorded diabetes	31640	(8.5)	763	(5.9)	811	(6.4)
Antidiabetics						
Insulin only	27121	(7.3)	1979	(15.2)	1528	(12.0)
OAD only	157653	(42.5)	4736	(36.4)	5029	(39.6)
Insulin & OAD	56481	(15.2)	2795	(21.5)	2456	(19.3)
Lipid-modifying agents	167593	(45.2)	7292	(56.0)	6472	(51.9)
Anti-hypertensives	313194	(84.4)	11849	(90.9)	11346	(89.3)
Anti-thrombotic drugs	108837	(29.3)	6250	(48.0)	5564	(43.8)
MI events	3774	(1.02)	456	(3.5)	211	(1.7)
Stroke events	4358	(1.17)	262	(2.0)	527	(4.1)
Target Period (July 2016 - June 2019) – Deaths and Events						
Total MI events	13030	(3.51)	13030	(100)	1259	(9.7)
Total stroke events	12701	(3.42)	1259	(9.7)	12701	(100)
Total deaths	32237	(8.7)	5058	(38.8)	3534	(27.8)
Total MI deaths	3568	(1.0)	3568	(27.4)	356	(2.8)
Total stroke deaths	1593	(0.4)	295	(2.3)	1593	(12.5)
CCI: Charlson Comorbidity Index (original dimension weights), DMP: Disease Management Program, MI: Myocardial Infarction, OAD: Oral Anti-Diabetic drugs						

Additional File 35. Training and test set characteristics

**Characteristic**	**Training Set**		**Validation Set**	
	**N**	**(%)**	**N**	**(%)**
N total (%)	296804	(100)	74202	(100)
Female	164078	(55.3)	41107	(55.4)
Mean age in years 2015 (mean, sd)	67.2 (9.45)		67.3 (9.41)	
Charlson Comorbidity Index in 2015 (mean, sd)	3.06 (2.16)		3.05 (2.17)	
DMP participation	193658	(65.2)	48400	(65.2)
Newly recorded diabetes in 2015	25290	(8.5)	6350	(8.6)
MI events in observation period	3007	(1.0)	767	(1.0)
Stroke events in observation period	3494	(1.2)	864	(1.2)
Total MI events in target period	10458	(3.5)	2572	(3.5)
Total stroke events in target period	10149	(3.4)	2552	(3.4)

Additional File 36. Events per variable

As dummy coding was applied to all categorical features in the logistic regression based and tree-based methods, the number of Events per Variable (EPV) in the training set was above the pre-determined threshold of 20, with EPV=32.3 (10458 individuals with event divided by 324 degrees of freedom (DoF)(78)) for MI and EPV=31.3 (10149 individuals with event divided by 324 DoF) for stroke. In deep learning, DoF and EPV are not reliable indicators for overfitting control, due to the nonlinearity of the models. Instead, we used regularization methods like Dropout and Early Stopping, coupled with close monitoring of the training and validation loss.

Additional File 37. Descriptives on the buffer period

Characteristic	Overall		MI in Target Period		Stroke in Target Period	
	N	(%)	N	(%)	N	(%)
N total (%)	371006	(100)	13030	(100)	12701	(100)
Female	205185	(55.31)	5222	(40.1)	6097	(48.0)
Buffer Period – Deaths and Events						
MI events	2586	(0.7)	426	(3.3)	149	(1.2)
Stroke events	2587	(0.7)	154	(1.2)	553	(4.4)
Total deaths	5039	(1.4)	0	(0)	0	(0)
Fatal MIs*	578	(0.2)	28	(0.2)	7	(0.1)
Fatal Strokes*	295	(0.1)	7	(0.1)	29	(0.2)

MI: Myocardial Infarction

* In some cases, individuals with MI or stroke events in two adjacent analysis periods (i.e. observation and buffer period, or buffer period and target period), occur in the dataset. In rare instances, these observations are an artificial result of coding decisions taken at the feature coding stage. These artefacts originated where individuals had hospitalizations for stroke or MI *and* the hospital stay stretched beyond the end of the previous analysis period into the consecutive analysis period (e.g. when a person stayed in hospital during the transition from observation to buffer period from December 31, 2015 to January 1, 2016, or during the transition from buffer to target period from June 30, 2016 to July 1, 2016). Diagnoses coded for a hospital stay were generally assigned to all quarters covered by the stay (assuming the diagnosis was causing the stay and would only be successfully treated with the end of stay).
When a hospital stay with a recorded MI or stroke diagnosis ended with a patient's death, we assumed the MI or stroke was the cause of death, in these cases coding a fatal event both for the quarter in which the hospitalization was initiated and the quarter in which the hospitalization ended.

Additional File 38. Results on discrimination performance

	Myocardial Infarction				Stroke			
**Model / Metric**	**AUPRC**	**(LCL; UCL)***	**AUROC**	**(LCL; UCL)**	**AUPRC**	**(LCL; UCL) ***	**AUROC**	**(LCL; UCL)**
NM	0.0347	(0.0333, 0.0359)	0.5000	(0.5000; 0.5000)	0.0344	(0.0331; 0.0356)	0.5000	(0.5000; 0.5000)
LMFWD	0.0897	(0.0831; 0.0978)	0.7102	(0.7001; 0.7202)	0.0701	(0.0657; 0.0758)	0.6815	(0.6712; 0.6917)
LMFULL	0.0893	(0.0830; 0.0970)	0.7100	(0.6999; 0.7201)	0.0715	(0.0667; 0.0777)	0.6817	(0.6714; 0.6920)
LASSO	0.0900	(0.0836; 0.0981)	0.7107	(0.7007; 0.7208)	0.0676	(0.0629; 0.0729)	0.6745	(0.6642; 0.6848)
RF	0.0899	(0.0830; 0.0982)	0.6999	(0.6895; 0.7102)	0.0716	(0.0668; 0.0776)	0.6793	(0.6691; 0.6896)
GB	0.0920	(0.0851; 0.1005)	0.7123	(0.7022; 0.7224)	0.0729	(0.0682; 0.0790)	0.6873	(0.6772; 0.6975)
MLP †	0.0819	(0.0761; 0.0893)	0.6962	(0.6859; 0.7065)	0.0651	(0.0607; 0.0763)	0.6561	(0.6454; 0.6667)
FTT †	0.0878	(0.0815; 0.0953)	0.7032	(0.6930; 0.7135)	0.0706	(0.0659; 0.0763)	0.6808	(0.6705; 0.6910)

Abbreviations: AUPRC = Area Under the Precision-Recall Curve, AUROC = Area Under the Receiver-Operator Curve, FTT = Feature-Tokenizer Transformer, GB = Gradient Boosting, LCL = Lower Confidence Limit, LMFWD = Logistic model with forward selection, LMFULL = Full logistic model with all features, MLP = Multi-Layer Perceptron, NM = Null Model, RF = Random Forest, UCL = Upper Confidence Limit.

***** PRAUC confidence intervals were calculated based on bootstrap methods with 2000 independent draws
† Calculation based on scaled probabilities from histogram binning

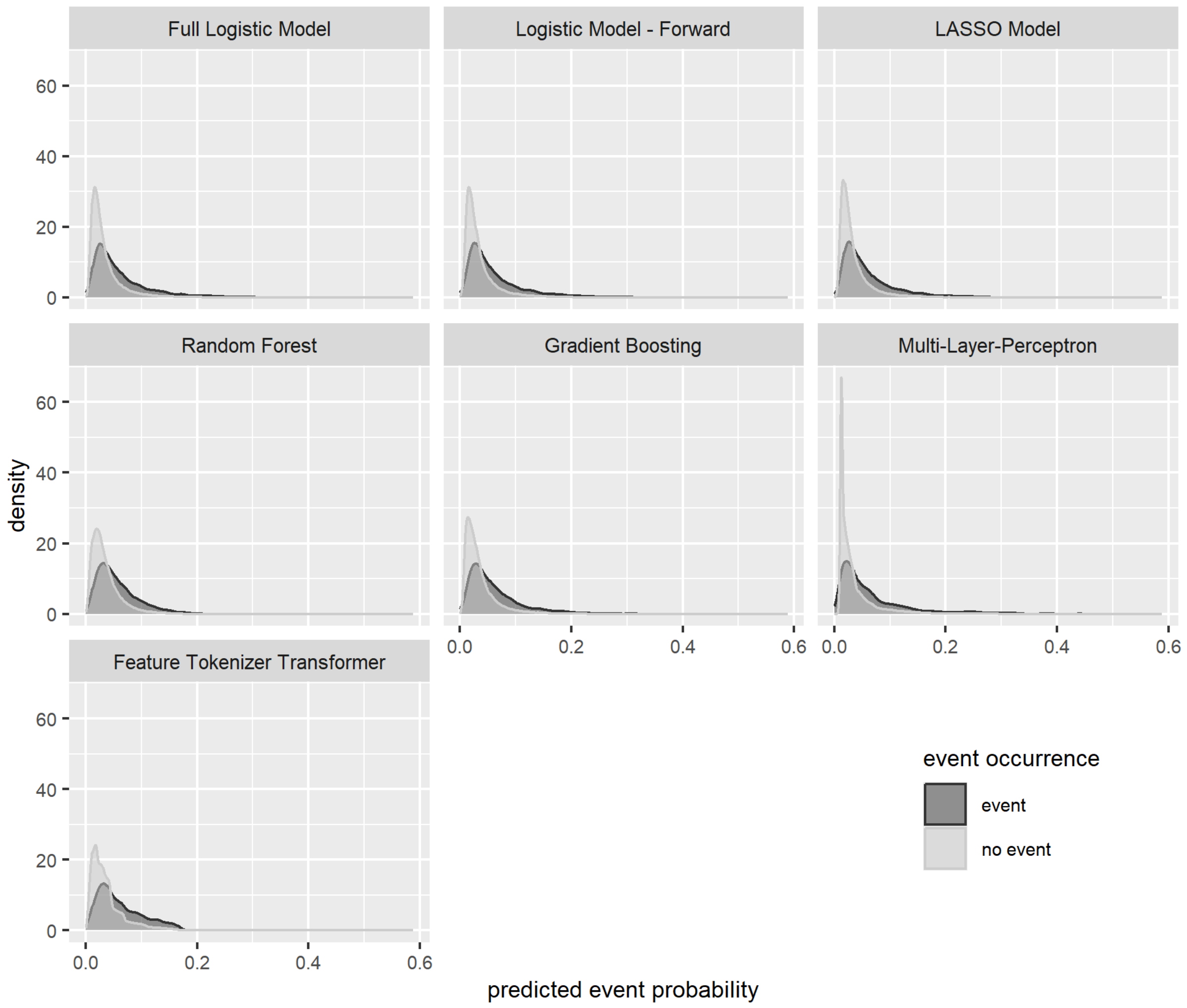

Additional File 39. Density plot of predicted MI probability for MI cases and non-cases

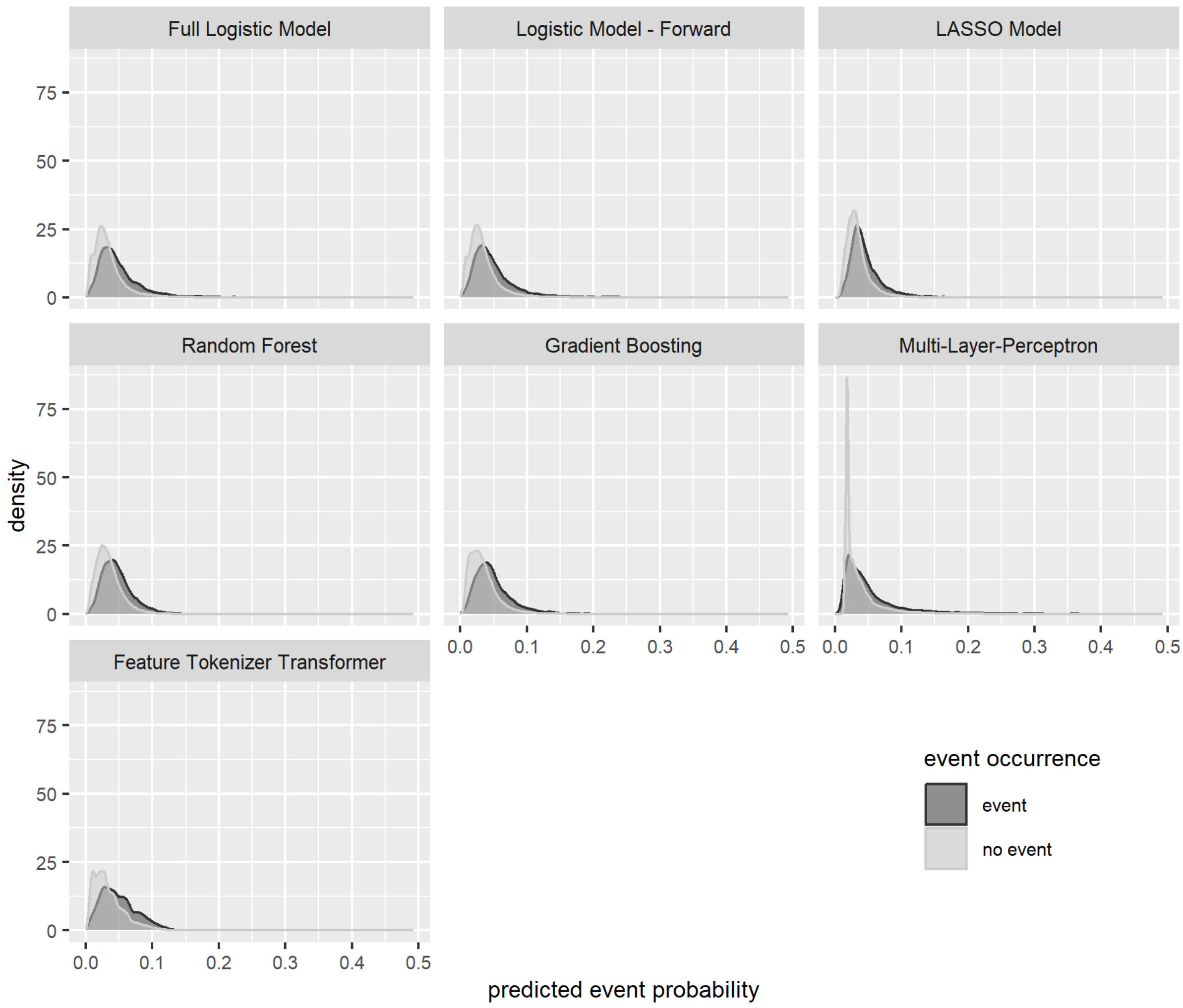

Additional File 40. Density plot of predicted Stroke probability for Stroke cases and non-cases

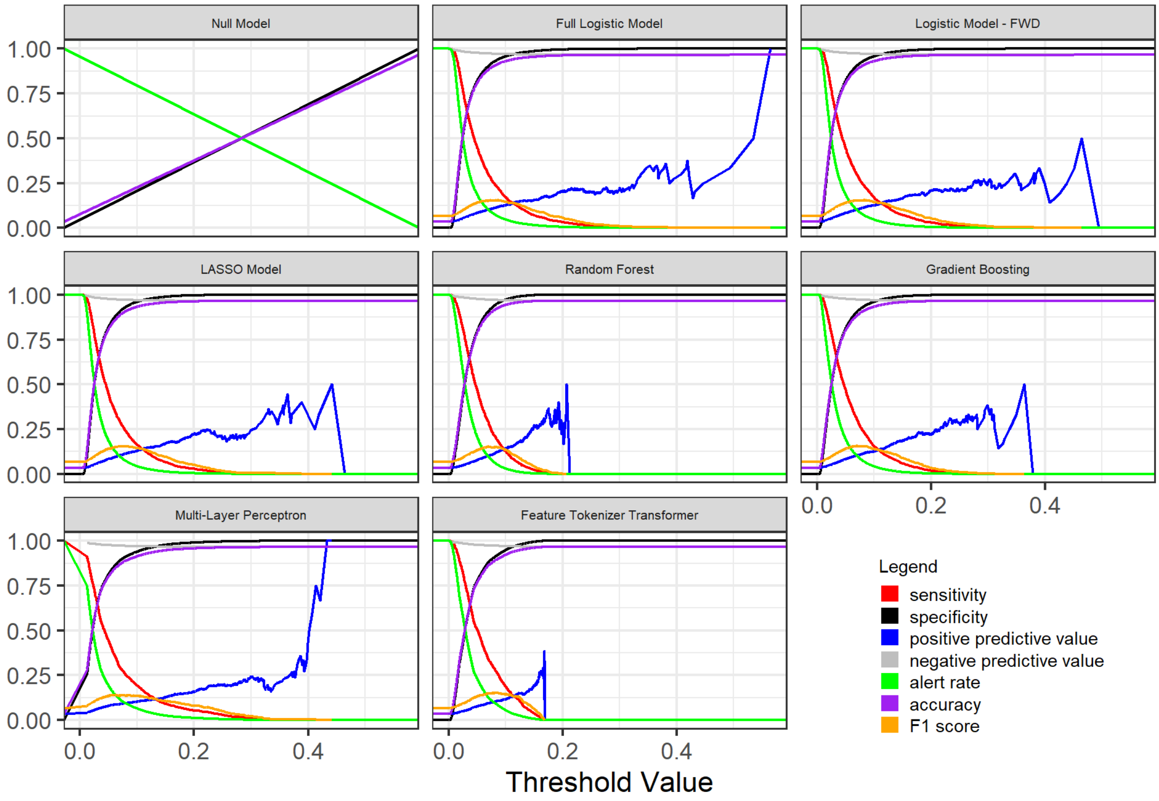

Additional File 41. Plot of classification metrics for MI against threshold.

Note: Ranges of predicted that could serve as classification thresholds varied between models. Depending on the classification metric, different thresholds maximized performance.

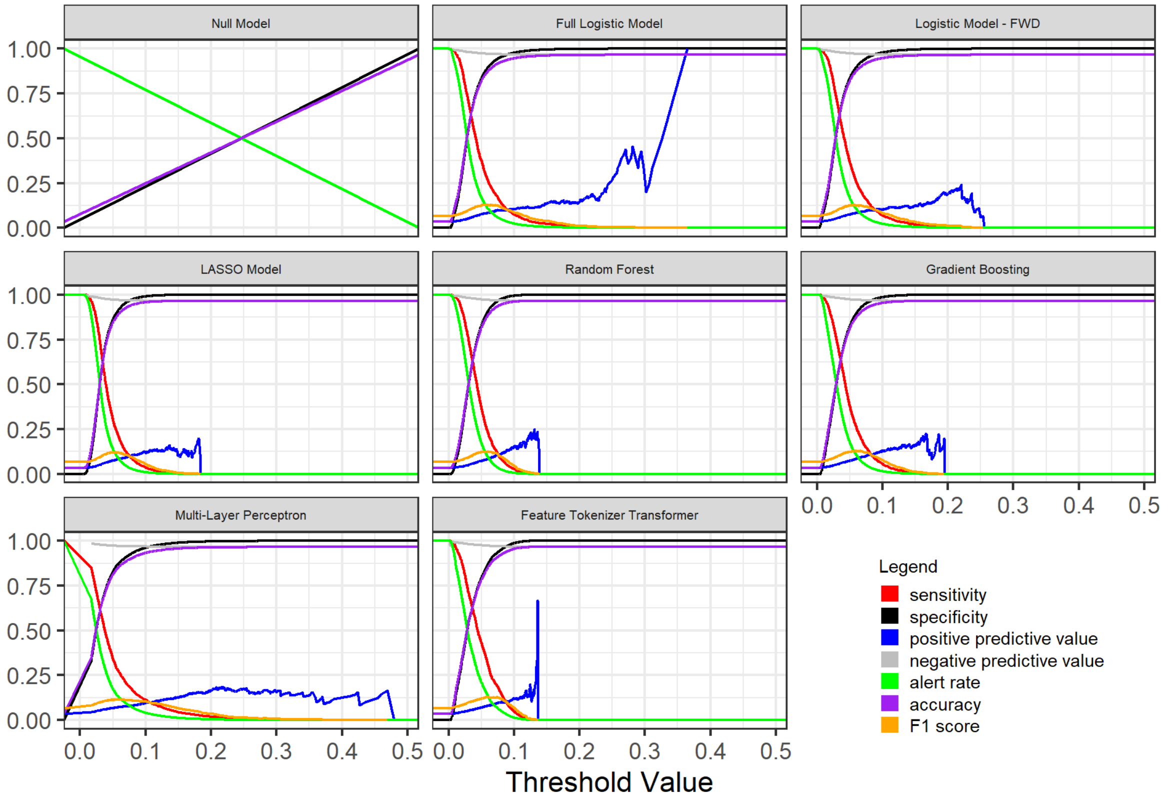

Additional File 42. Plot of classification metrics for Stroke against threshold.
Note: Ranges of predicted that could serve as classification thresholds varied between models. Depending on the classification metric, different thresholds maximized performance.

Additional File 43. Results on classification performance at the respective optimized classification threshold

Model / Metric	F1 score at threshold	Threshold value	PPV	NPV *	Sensitivity	Specificity *	NNE	Alert rate	Negative Likelihood Ratio*	Positive Likelihood Ratio	Accuracy
**MI**											
NM	0.067	0.0352	0.035	NaN	1	0	29	1	NaN	1	0.0347
LOGM FULL	0.1553	0.0835	0.116	0.972	0.237	0.935	9	0.071	0.8	3.6	0.9107
LOGM FWD	0.1555	0.0862	0.119	0.971	0.223	0.941	8	0.065	0.8	3.8	0.9163
LASSO	0.1556	0.0685	0.104	0.973	0.306	0.906	10	0.102	0.8	3.2	0.8849
RF	0.1528	0.0708	0.105	0.973	0.282	0.913	10	0.093	0.8	3.3	0.8915
GB	0.1568	0.0711	0.106	0.973	0.301	0.909	9	0.099	0.8	3.3	0.8877
FTT	0.1402	0.0637	0.088	0.974	0.342	0.873	11	0.135	0.8	2.7	0.8545
MLP	0.1525	0.0823	0.105	0.973	0.28	0.914	10	0.092	0.8	3.3	0.8923
**Stroke**											
NM	0.0665	0.0342	0.034	NaN	1	0	29	1	NaN	1	0.0344
LOGM FULL	0.1295	0.053	0.08	0.974	0.345	0.858	13	0.149	0.8	2.4	0.8405
LOGM FWD	0.1285	0.0561	0.082	0.973	0.301	0.879	12	0.127	0.8	2.5	0.8594
LASSO	0.1217	0.0498	0.076	0.973	0.314	0.863	13	0.143	0.8	2.3	0.8441
RF	0.1259	0.0602	0.086	0.971	0.236	0.911	12	0.094	0.8	2.6	0.8876
GB	0.1299	0.0663	0.094	0.971	0.212	0.927	11	0.078	0.9	2.9	0.9025
FTT	0.1151	0.0602	0.074	0.971	0.266	0.881	14	0.124	0.8	2.2	0.8595
MLP	0.128	0.0591	0.08	0.973	0.328	0.865	13	0.142	0.8	2.4	0.8464

NM = Null Model, LOGM FWD = Logistic model with forward selection, LOGM FULL = Full logistic model with all features, RF = Random Forest, GB = Gradient Boosting, MLP = Multi-Layer Perceptron, FTT = Feature Tokenizer Transformer, AUPRC = Area Under the Precision-Recall Curve, LCL = Lower Confidence Limit, UCL = Upper Confidence Limit, AUROC = Area Under the Receiver-Operator Curve

*Specificity, NPV and NLR may be less relevant in contexts when the focus is on identification of the positive class, especially when this positive class is the minority class. In such situations, low NLR, high specificity and high NPV may misleadingly suggest good model performance even if the predictive power for the positive class is low. This tendency is exacerbated with increasing class imbalance.

Additional File 44. Brier score

	Brier Score	
**Model**	**Myocardial Infarction**	**Stroke**
Null model	0.0335	0.0332
Logistic (forward selection)	0.0327	0.0327
Logistic (full model)	0.0326	0.0327
LASSO	0.0326	0.0328
Random Forest	0.0327	0.0327
Gradient Boosting	0.0326	0.0327
FTT	0.033	0.033
MLP	0.0327	0.0327

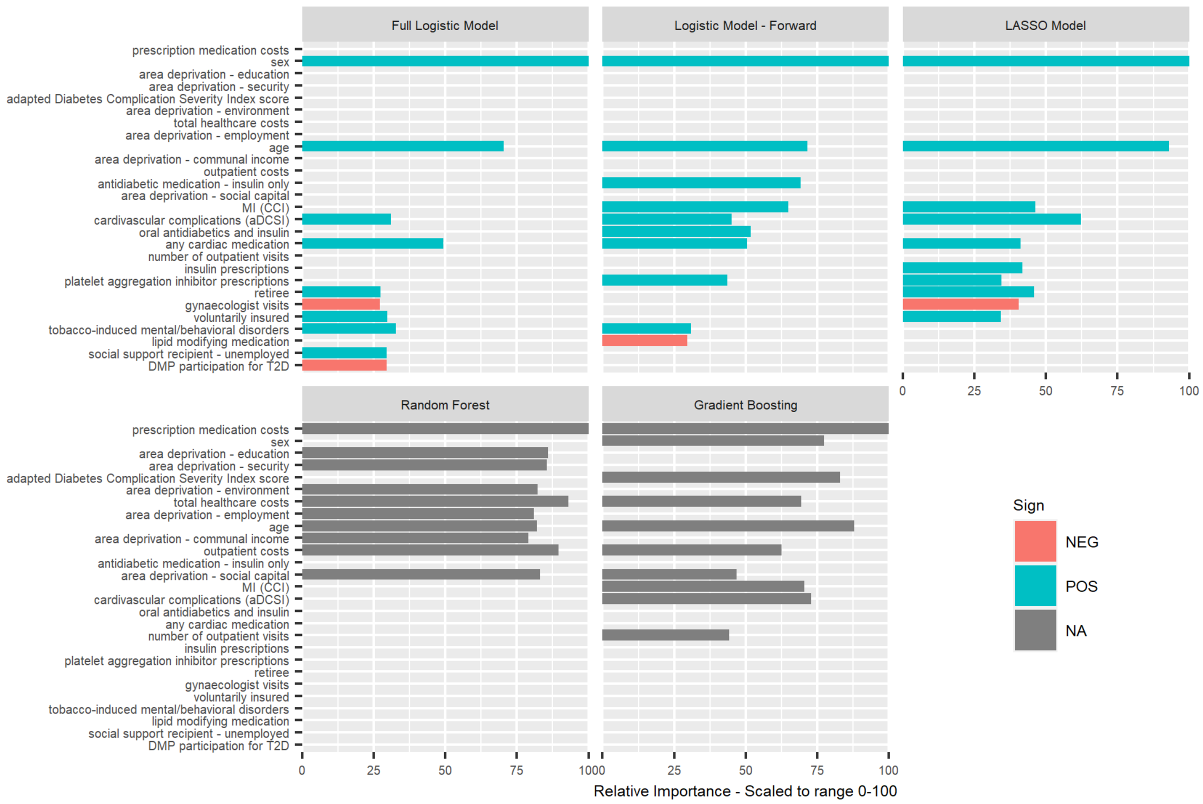

Additional File 45. Variable Importance for MI models (10 most important variables for each model)

Note: For each panel, the x-axis represents the relative variable importance of the variables depicted on the y-axis in the respective model. The y-axis lists all variables that are among the ten most important variables in at least one of the five depicted models. Variable importance is scaled to range between 0 (not important) and 100 (highest importance). Bar lengths indicate relative variable importance, but do not convey information on absolute importance. Variables without bars may have scaled importance values >0 for a model, but the respective values are not shown if a variable does not fall under the respective model's 10 most important variables. Color codes for bars in the logistic regression-based models indicate the direction of the respective variable's coefficient sign (negative coefficient indicating lower risk: red; positive coefficient indicating increased risk: blue).

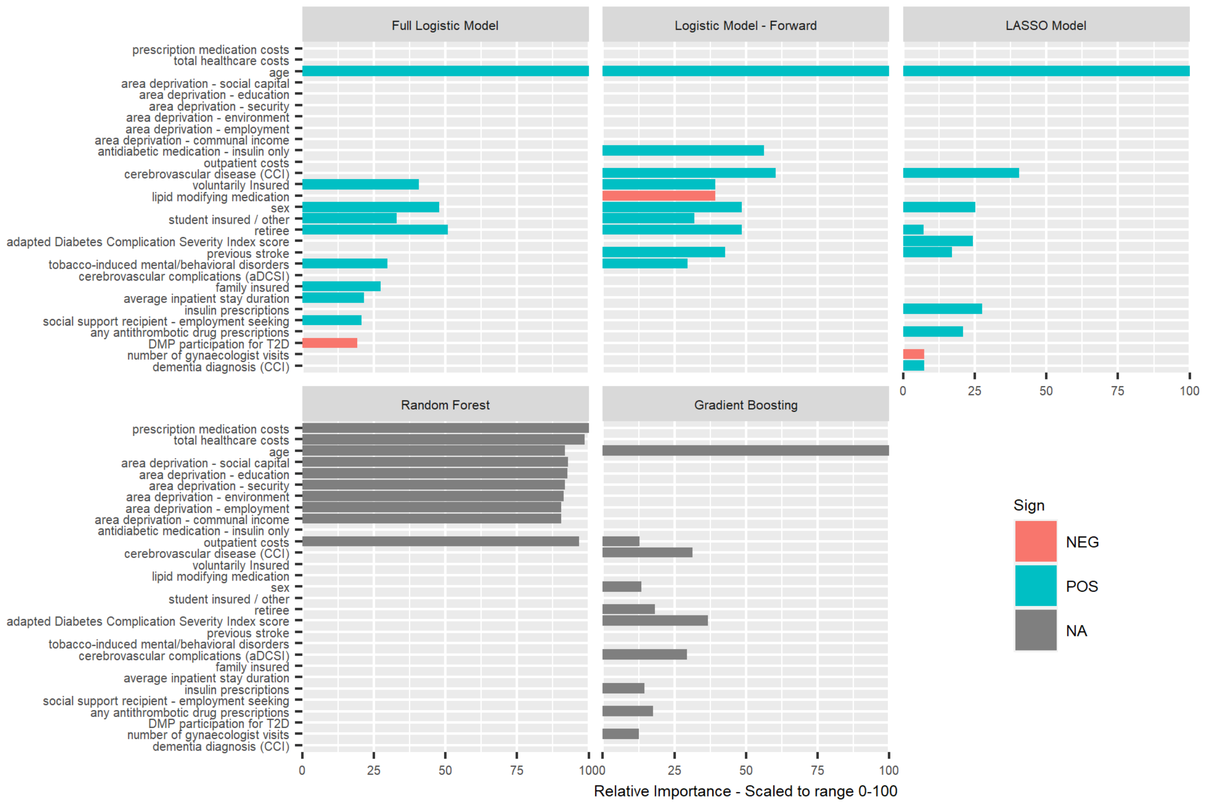

Additional File 46. Variable Importance for stroke models (10 most important variables for each model)

Note: For each panel, the x-axis represents the relative variable importance of the variables depicted on the y-axis in the respective model. The y-axis lists all variables that are among the ten most important variables in at least one of the five depicted models. Variable importance is scaled to range between 0 (not important) and 100 (highest importance). Bar lengths indicate relative variable importance, but do not convey information on absolute importance. Variables without bars may have scaled importance values >0 for a model, but the respective values are not shown if a variable does not fall under the respective model's 10 most important variables. Color codes for bars in the logistic regression-based models indicate the direction of the respective variable's coefficient sign (negative coefficient indicating lower risk: red; positive coefficient indicating increased risk: blue).

Additional File 47. Most important variables for MI prediction models

	**Model**				
**Rank**	**Full Logistic Model**	**Logistic Model - FWD**	**LASSO**	**Random Forest**	**Gradient Boosting**
**1**	Sex	Sex	Sex	Sum of prescription medication costs	Sum of prescription medication costs
**2**	Age	Age	Age	Overall healthcare costs	Age
**3**	any cardiac medication	Type of antidiabetic medication prescriptions: Insulin only	cardivascular complications (aDCSI)	Sum of outpatient costs	Adapted Diabetes Complications Severity Index: Total score
**4**	mental and behavioural disorders due to use of tobacco	Previous MI (CCI)	Previous MI (CCI)	area deprivation - security	cardivascular complications (aDCSI)
**5**	cardivascular complications (aDCSI)	Type of antidiabetic medication prescriptions: Insulin and OAD	Retiree	area deprivation - education	Sex
**6**	Voluntarily insured	any cardiac medication	Any insulin prescriptions	Age	Sum of outpatient costs
**7**	Social support recipient – unemployment (SGBII)	cardivascular complications (aDCSI)	any cardiac medication	area deprivation - social capital	Overall healthcare costs
**8**	DMP participation for T2D	Thrombosis medication – platelet aggregation inhibitors prescriptions	Number of gynaecologist visits	area deprivation - environment	Previous MI (CCI)
**9**	Retiree	mental and behavioural disorders due to use of tobacco	Thrombosis medication – platelet aggregation inhibitors prescriptions	area deprivation - employment	area deprivation - security
**10**	Number of gynaecologist visits	Any Lipid modifying medication prescriptions	Voluntarily insured	area deprivation - income	area deprivation - income
**11**	ischemic heart disease record	DMP participation for T2D	Diabetes medication – oral antidiabetic drug prescriptions	area deprivation - communal income	area deprivation – education
**12**	CNS medication – analgesics	Voluntarily insured	Type of antidiabetic medication prescriptions: Insulin only	German Index of Multiple Deprivation – Total Score	area deprivation – employment
**13**	Social support recipient – employment seeking (SGBIII)	Social support recipient – unemployment (SGBII)	Social support recipient – unemployment (SGBII)	Sum of outpatient visits	Sum of outpatient visits
**14**	Number of urologist visits	CNS medication – analgesics prescriptions	Federal state: Sachsen	Elixhauser Index: Total score with Swiss weights	ischemic heart disease record
**15**	Federal state: Sachsen	Atrial fibrillation	Any Lipid modifying medication prescriptions	Number of general practitioner visits	area deprivation – environment
**16**	Atrial fibrillation	Retiree	DMP participation for T2D	Elixhauser Index: Weighted total score	area deprivation - social capital
**17**	Peripheral vascular complications (aDCSI)	Number of gynaecologist visits	Number of gynaecology endocrinologist and reporoductive medicine physician visits	Number of internist visits	German Index of Multiple Deprivation – Total Score
**18**	Corticosteroid prescriptions	Diabetes medication – second generation sulfonylurea prescriptions	mental and behavioural disorders due to use of tobacco	Number of laboratory medicine visits	area deprivation - communal income
**19**	Cardiac medication – cardiac glycosides prescriptions	thyroid therapy prescriptions	Peripheral vascular complications (aDCSI)	Sum of aids and remedies costs	Thrombosis medication – platelet aggregation inhibitors prescriptionsCharlson Comorbidity Index: Unweighted total score
**20**	Number of dermatologist visits	5 antihypertensive drug types prescribed	Atrial fibrillation	Elixhauser Index: Unweighted total score	Number of gynaecologist visits

**21**	Charlson Comorbidity Index: Updated weighted total score	ischemic heart disease record	Solid tumor without metastasis (EHI)	Number of ophthalmologist visits	
**22**	Cardiac medication – antiarrhythmic prescriptions	Peripheral vascular complications (aDCSI)	CNS medication – analgesics prescriptions	Sum of inpatient costs	Thrombosis medication – platelet aggregation inhibitors prescriptions
**23**	Number of vascular surgeon visits	4 antihypertensive drug types prescribed	Hypertension medication – diuretics prescriptions	Sum of devices costs	Elixhauser Index: Total score with Swiss weights
**24**	CNS medication – tramadol prescriptions	3 antihypertensive drug types prescribed	thyroid therapy prescriptions	Sum of visits to a physiotherapist	Sum of inpatient costs
**25**	Number of psychological psychotherapist visits	Federal state: Sachsen	Diabetes medication – second generation sulfonylurea prescriptions	adapted Diabetes Complication Severity Index score	Charlson Comorbidity Index: Total score
**26**	Family insured	Social support recipient – employment seeking (SGBIII)	Rheumatoid arthritis (CCI)	Average duration in hospital	Sum of devices costs
**27**	COPD record	Number of urologist visits	Number of psychological psychotherapist visits	Charlson Comorbidity Index: Total score	Number of general practitioner visits
**28**	dyslipidemia	CNS medication – antipsychotics prescriptions	Social support recipient – employment seeking (SGBIII)	Charlson Comorbidity Index: Updated weighted total score	Sum of oupatient visits to internist (family doctor) in 2015
**29**	Sum of devices costs	Type of antidiabetic medication prescriptions: OAD only	CNS medication – antipsychotics prescriptions	Number of orthopedic visits	Sum of days spent in hospital
**30**	Newly recorded diabetes	adapted Diabetes Complication Severity Index score	Renal complications (aDCSI)	Sum of days spent in hospital	Sum of aids and remedies costs

Additional File 48. Most important variables for stroke prediction models

	Model				
**Rank**	**Full Logistic Model**	**Logistic Model - FWD**	**LASSO**	**Random Forest**	**Gradient Boosting**
**1**	Age	Age	Age	Sum of medication costs	Age
**2**	Retiree	Cerebrovascular disease (CCI)	Cerebrovascular disease (CCI)	Overall healthcare costs	adapted Diabetes Complication Severity Index score
**3**	Sex	Type of antidiabetic medication prescriptions: Insulin only	Diabetes medication – insulin prescriptions	Sum of outpatient costs	Cerebrovascular disease (CCI)
**4**	Voluntarily insured	Retiree	Sex	area deprivation - security	Cerebrovascular complications (aDCSI)
**5**	student insured / other	Sex	adapted Diabetes Complication Severity Index score	area deprivation - social capital	Retiree
**6**	mental and behavioural disorders due to use of tobacco	Previous cerebrovascular complications	Thrombosis medication – antithrombotic drugs prescriptions	area deprivation - education	Thrombosis medication – antithrombotic drugs prescriptions
**7**	Family insured	Voluntarily insured	Previous cerebrovascular complications	Age	Diabetes medication – insulin prescriptions
**8**	Average duration in hospital	Any Lipid modifying medication prescriptions	Dementia (CCI)	area deprivation - environment	Sum of outpatient costs
**9**	Social support recipient – employment seeking (SGBIII)	student insured / other	Number of gynaecologist visits	area deprivation - communal income	Sex
**10**	DMP participation for T2D	mental and behavioural disorders due to use of tobacco	Retiree	area deprivation - employment	Sum of medication costs
**11**	Previous cerebrovascular complications	Type of antidiabetic medication prescriptions: Insulin and OAD	Thrombosis medication – platelet aggregation inhibitors prescriptions	area deprivation - income	Number of gynaecologist visits
**12**	Sum of inpatient costs	Family insured	Paralysis (CCI)	German Index of Multiple Deprivation – Total Score	Overall healthcare costs
**13**	Number of gynaecologist visits	Obesity (EHI)	Other neurological disorders (EHI)	Sum of outpatient visits	Previous cerebrovascular complications
**14**	Sum of stays in rehabilitation	Average duration in hospital	Peripheral vascular disease (CCI)	Elixhauser Index: Total score with Swiss weights	Number of ophthalmologist visits
**15**	Social support recipient – unemployment (SGBII)	Hypertension medication – renin-angiotensin system acting agents presciptions	low outpatient care users (< 3 outpatient visits)	Number of general practitioner visits	Charlson Comorbidity Index: Unweighted total score
**16**	Lipid modifying medication – statin prescriptions	Hypertension records	any antihypertensive drugs prescriptions	Elixhauser Index: Weighted total score	Charlson Comorbidity Index: Total score
**17**	Plain lipid modifying medication prescription	5 antihypertensive drug types prescribed	Diabetes medication – second generation sulfonylurea prescriptions	Number of internist visits	Sum of inpatient costs
**18**	Number of urologist visits	Sum of inpatient costs	diabetic foot record	Number of laboratory medicine visits	Cardiovascular complications (aDCSI)
**19**	Any antihypertensive drugs prescriptions	mental and behavioural disorders due to use of alcohol	mental and behavioural disorders due to use of tobacco	Sum of aids and remedies costs	area deprivation - security
**20**	Thrombosis medication – warfarin prescriptions	Elixhauser Index: Weighted total score	Type of antidiabetic medication prescriptions: Insulin only	Elixhauser Index: Unweighted total score	Thrombosis medication – platelet aggregation inhibitors prescriptions
**21**	area deprivation - environment	Diabetes medication – second generation sulfonylurea prescriptions	mental and behavioural disorders due to use of alcohol	Number of ophthalmologist visits	Sum of outpatient visits

**22**	Cardiac medication – cardiac glycosides prescriptions	DMP participation for T2D	Average duration in hospital	Sum of visits to a physiotherapist	Dementia (CCI)
**23**	mental and behavioural disorders due to use of alcohol	Dementia (CCI)	Cardiac medication – cardiac glycosides prescriptions	Sum of inpatient costs	Sum of days spent in hospital
**24**	proteinuria (incl. albuminuria)	Social support recipient – employment seeking (SGBIII)	1 thrombosis medication category prescribed	adapted Diabetes Complication Severity Index score	Elixhauser Index: Weighted total score
**25**	Obesity (EHI)	Other neurological disorders (EHI)	Neuropathy records	Charlson Comorbidity Index: Total score	Elixhauser Index: Total score with Swiss weights
**26**	Rural district with population concentration	Thrombosis medication – heparin prescriptions	area deprivation - income	Number of orthopedic visits	area deprivation - education
**27**	Urban district	4 antihypertensive drug types prescribed	area deprivation - employment	Number of orthopedic visits	Type of antidiabetic medication prescriptions: Insulin only
**28**	Any Lipid modifying medication prescriptions	Paralysis (CCI)	area deprivation - education	Sum of devices costs	area deprivation - social capital
**29**	Hypertension medication – other agents acting on the renin-angiotensin system prescribed	diabetes without complications	area deprivation - communal income	Average duration in hospital	Average duration in hospital
**30**	End-stage renal disease	Sum of outpatient visits	area deprivation - social capital	Sum of days spent in hospital	Paralysis (CCI)

Additional File 49. Discussion of additional technical aspects

The comparative approach of our project allows the conclusion that, depending on the data context, computationally intensive methods do not necessarily outperform simpler approaches. However, comparability required the same set of features, even though for some methods (e.g. the full logistic model) a more stringent exclusion of collinear features would have been preferable, while others (e.g. tree-based) would have handled even more collinearities.

Surprisingly, for stroke LASSO performed worse than the logistic models, even though LASSO in theory comprises the possibility to set penalties to zero, resulting in a full logistic model. However, the stroke LASSO encountered convergence problems for small penalties starting with the 25th lambda value (presumably due to the remaining multicollinearities in the features), thus returning only solutions for larger-than ideal penalty values. Apparently, also the *glmnet* function with which LASSO was implemented handles collinearities differently from the *glm* function which was used for the logistic models: When we tried to replicate the full logistic model with *glmnet*, forcing in a close-to-zero penalty, *glmnet* still set most coefficients to zero, whereas the full model *glm* did only remove a handful of coefficients to avoid collinearity issues. Also the forward selection kept a relatively large number of variables in the model. In sum, it is possible that for this research question the logistic models (in contrast to, for example the deep learning models) did not actually encounter overfitting issues, which makes variable selection less important and would suggest that models should use as much of the available information as possible. Therefore, Ridge regression and the approaches that do not use variable selection (such as the full logistic model) or use it less strictly (such as the forward selection), may potentially outperform LASSO in our case.

Competing events occurred also in our project. For individuals who died during the target period, keeping them in the dataset allowed them to contribute stroke and myocardial infarction events before death at the cost of ignoring competing risks.

Keeping individuals who died during the buffer period in the data set may have weakened the predictive power of our models because all individuals who died from stroke and MI during the buffer period were misclassified throughout the target period. This was a conservative decision for pragmatic reasons, as mortality information for the buffer period may be unavailable or incomplete at the time of model application. No additional adjustments were made to account for this possible source of error in the analysis. Assigning main diagnoses for hospitalizations always to the start date of the hospitalization period may have introduced some additional misclassification as deaths introduce the end of the stay. This led to the counterintuitive fact that a fatal MI was attributed to the beginning of a hospitalization. For those hospitalizations that ended in death and bridged two quarters assigned to different participant time windows (e.g. observation and buffer period, or buffer and target period), this means that a fatal MI in the beginning of the latter period was erroneously assigned to the preceding period.

Lastly, the single split into training and test sets is considered an inefficient way to correct for optimism (4, 78) due to the reduced training sample(4). However, we were confident that this split-sample approach left large enough samples to produce sufficiently precise estimates and feel that the simplicity of the approach outweighed potential efficiency losses. Furthermore, even though in our case the chosen unconditional sampling approach resulted in the desired balance of events across training and test data sets, we would like to note that in some cases it may be worth considering a stratified sampling approach when conducting the train-test sample split. Such stratified sampling ensures an even distribution of events across training and test data sets, and may be especially helpful in situations with smaller overall sample sizes.

It should be noted that it is frequently recommended to use over- and undersampling techniques to create a balanced dataset for training purposes in situations with highly imbalanced outcome classes. We applied such techniques for the two deep learning but not for other approaches. The fact that approaches with and without over- and undersampling yielded similar performance results suggests that this may not have been a main driver of observed model performance. Though not particularly effective in our specific DL models, we would like to point out that authors might want to consider the use of over- and undersampling techniques in similar situations for with highly imbalanced outcome data, independently of the specific applied algorithm. In cases where such over- and undersampling is applied, a special focus should be on potential implications for the resulting model's predicted probabilities. Rescaling methods such as histogram binning or temperature scaling may subsequently be required.

With regard to variable importance, we would like to add a last note of caution, as for highly imbalanced datasets, identification of important variables may become less reliable.(79) Also, for random forests, variable importance based on impurity has been reported to have a tendency to pick metric predictors or predictors with many categories more often than dichotomous predictors, but might still be the preferable option for cases with highly skewed outcome distributions.(64)

References

1. Cieslak DA, Hoens TR, Chawla NV, Kegelmeyer WP. Hellinger distance decision trees are robust and skew-insensitive. Data Mining and Knowledge Discovery. 2012;24:136-58.
2. Kähm K, Laxy M, Schneider U, Rogowski WH, Lhachimi SK, Holle R. Health care costs associated with incident complications in patients with type 2 diabetes in Germany. Diabetes Care. 2018;41(5):971-8.
3. Robert-Koch-Institut. Nationale Diabetes-Surveillance - Diabetes in Deutschland - Erwachsene - Mortalität 2020 [Available from: https://diabsurv.rki.de/Webs/Diabsurv/DE/diabetes-in-deutschland/4-36_Mortalitaet.html.
4. Moons KG, Wolff RF, Riley RD, Whiting PF, Westwood M, Collins GS, et al. PROBAST: a tool to assess risk of bias and applicability of prediction model studies: explanation and elaboration. Annals of internal medicine. 2019;170(1):W1-W33.
5. Maier W. Indices of Multiple Deprivation for the analysis of regional health disparities in Germany: Experiences from epidemiology and healthcare research. Bundesgesundheitsblatt, Gesundheitsforschung, Gesundheitsschutz. 2017;60(12):1403-12.
6. Maier W, Schwettmann L, editors. Regionale Deprivation in Deutschland: Der ‚German Index of Multiple Deprivation (GIMD)'. Public Health Forum; 2018: De Gruyter.
7. Schederecker F, Kurz C, Fairburn J, Maier W. Do alternative weighting approaches for an Index of Multiple Deprivation change the association with mortality? A sensitivity analysis from Germany. BMJ open. 2019;9(8):e028553.
8. Karter AJ, Warton EM, Lipska KJ, Ralston JD, Moffet HH, Jackson GG, et al. Development and validation of a tool to identify patients with type 2 diabetes at high risk of hypoglycemia-related emergency department or hospital use. JAMA internal medicine. 2017;177(10):1461-70.
9. Mueller L, Berhanu P, Bouchard J, Alas V, Elder K, Thai N, et al. Application of machine learning models to evaluate hypoglycemia risk in type 2 diabetes. Diabetes Therapy. 2020;11(3):681-99.
10. Schroeder EB, Xu S, Goodrich GK, Nichols GA, O'Connor PJ, Steiner JF. Predicting the 6-month risk of severe hypoglycemia among adults with diabetes: development and external validation of a prediction model. Journal of Diabetes and its Complications. 2017;31(7):1158-63.
11. Vogt V, Koller D, Sundmacher L. Continuity of care in the ambulatory sector and hospital admissions among patients with heart failure in Germany. The European Journal of Public Health. 2016;26(4):555-61.
12. Ionescu-Ittu R, McCusker J, Ciampi A, Vadeboncoeur A-M, Roberge D, Larouche D, et al. Continuity of primary care and emergency department utilization among elderly people. Cmaj. 2007;177(11):1362-8.
13. Warren JR, Falster MO, Tran B, Jorm L. Association of continuity of primary care and statin adherence. PLoS One. 2015;10(10):e0140008.
14. Mehta HB, Dimou F, Adhikari D, Tamirisa NP, Sieloff E, Williams TP, et al. Comparison of comorbidity scores in predicting surgical outcomes. Medical care. 2016;54(2):180.
15. Quan H, Sundararajan V, Halfon P, Fong A, Burnand B, Luthi J-C, et al. Coding algorithms for defining comorbidities in ICD-9-CM and ICD-10 administrative data. Medical care. 2005:1130-9.
16. Charlson ME, Pompei P, Ales KL, MacKenzie CR. A new method of classifying prognostic comorbidity in longitudinal studies: development and validation. Journal of chronic diseases. 1987;40(5):373-83.
17. Han K, Yun J-S, Park Y-M, Ahn Y-B, Cho J-H, Cha S-A, et al. Development and validation of a risk prediction model for severe hypoglycemia in adult patients with type 2 diabetes: a nationwide population-based cohort study. Clinical epidemiology. 2018;10:1545.
18. Quan H, Li B, Couris CM, Fushimi K, Graham P, Hider P, et al. Updating and Validating the Charlson Comorbidity Index and Score for Risk Adjustment in Hospital Discharge Abstracts Using Data From 6 Countries. American Journal of Epidemiology. 2011;173(6):676-82.

19. Elixhauser A, Steiner C, Harris DR, Coffey RM. Comorbidity measures for use with administrative data. Medical care. 1998:8-27.
20. van Walraven C, Austin PC, Jennings A, Quan H, Forster AJ. A modification of the Elixhauser comorbidity measures into a point system for hospital death using administrative data. Medical care. 2009:626-33.
21. Sharma N, Schwendimann R, Endrich O, Ausserhofer D, Simon M. Comparing Charlson and Elixhauser comorbidity indices with different weightings to predict in-hospital mortality: an analysis of national inpatient data. BMC health services research. 2021;21(1):1-10.
22. Wicke FS, Glushan A, Schubert I, Köster I, Luebeck R, Hammer M, et al. Performance of the adapted Diabetes Complications Severity Index translated to ICD-10. The American Journal of Managed Care. 2019;25(2):e45-e9.
23. Glasheen WP, Renda A, Dong Y. Diabetes complications severity index (DCSI)—update and ICD-10 translation. Journal of Diabetes and its Complications. 2017;31(6):1007-13.
24. Elley CR, Robinson T, Moyes SA, Kenealy T, Collins J, Robinson E, et al. Derivation and validation of a renal risk score for people with type 2 diabetes. Diabetes care. 2013;36(10):3113-20.
25. Cederholm J, Eeg-Olofsson K, Eliasson B, Zethelius B, Nilsson PM, Gudbjörnsdottir S. Risk prediction of cardiovascular disease in type 2 diabetes: a risk equation from the Swedish National Diabetes Register. Diabetes care. 2008;31(10):2038-43.
26. Yang X, Ma RC, So W-Y, Kong AP, Ko GT, Ho C-S, et al. Development and validation of a risk score for hospitalization for heart failure in patients with Type 2 diabetes mellitus. Cardiovascular diabetology. 2008;7(1):1-8.
27. Cichosz SL, Johansen MD, Knudsen ST, Hansen TK, Hejlesen O. A classification model for predicting eye disease in newly diagnosed people with type 2 diabetes. Diabetes research and clinical practice. 2015;108(2):210-5.
28. Chang H-Y, Weiner JP, Richards TM, Bleich SN, Segal J. Validating the adapted Diabetes Complications Severity Index in claims data. The American journal of managed care. 2012;18(11):721-6.
29. Young BA, Lin E, Von Korff M, Simon G, Ciechanowski P, Ludman EJ, et al. Diabetes complications severity index and risk of mortality, hospitalization, and healthcare utilization. The American journal of managed care. 2008;14(1):15.
30. Mehlsen J, Erlandsen M, Poulsen PL, Bek T. Identification of independent risk factors for the development of diabetic retinopathy requiring treatment. Acta ophthalmologica. 2011;89(6):515-21.
31. Donnan PT, Donnelly L, New JP, Morris AD. Derivation and validation of a prediction score for major coronary heart disease events in a UK type 2 diabetic population. Diabetes Care. 2006;29(6):1231-6.
32. Folsom AR, Chambless LE, Duncan BB, Gilbert AC, Pankow JS, Investigators ARiCS. Prediction of coronary heart disease in middle-aged adults with diabetes. Diabetes care. 2003;26(10):2777-84.
33. Kothari V, Stevens RJ, Adler AI, Stratton IM, Manley SE, Neil A. Risk of Stroke in Type 2 Diabetes Estimated by the UK Prospective Diabetes Study Risk Engine (UKPDS 60). Stroke. 2002;33:1776-81.
34. Stevens RJ, Kothari V, Adler AI, Stratton IM, Holman RR, Group UKPDS. The UKPDS risk engine: a model for the risk of coronary heart disease in Type II diabetes (UKPDS 56). Clinical science. 2001;101(6):671-9.
35. Kengne AP, Patel A, Marre M, Travert F, Lievre M, Zoungas S, et al. Contemporary model for cardiovascular risk prediction in people with type 2 diabetes. European Journal of Cardiovascular Prevention & Rehabilitation. 2011;18(3):393-8.
36. Aminian A, Zajichek A, Arterburn DE, Wolski KE, Brethauer SA, Schauer PR, et al. Predicting 10-year risk of end-organ complications of type 2 diabetes with and without metabolic surgery: a machine learning approach. Diabetes Care. 2020;43(4):852-9.

37. Yang X, So W-Y, Kong AP, Ma RC, Ko GT, Ho C-S, et al. Development and validation of a total coronary heart disease risk score in type 2 diabetes mellitus. The American journal of cardiology. 2008;101(5):596-601.
38. Aspinall PA, Kinnear PR, Duncan LJ, Clarke BF. Prediction of diabetic retinopathy from clinical variables and color vision data. Diabetes Care. 1983;6(2):144-8.
39. Keane WF, Zhang Z, Lyle PA, Cooper ME, de Zeeuw D, Grunfeld J-P, et al. Risk scores for predicting outcomes in patients with type 2 diabetes and nephropathy: the RENAAL study. Clinical journal of the American Society of Nephrology. 2006;1(4):761-7.
40. Yang X, So W-Y, Kong AP, Ho C-S, Lam CW, Stevens RJ, et al. Development and validation of stroke risk equation for Hong Kong Chinese patients with type 2 diabetes: the Hong Kong Diabetes Registry. Diabetes care. 2007;30(1):65-70.
41. Davis W, Knuiman M, Davis T. An Australian cardiovascular risk equation for type 2 diabetes: the Fremantle Diabetes Study. Internal medicine journal. 2010;40(4):286-92.
42. Boyko EJ, Ahroni JH, Cohen V, Nelson KM, Heagerty PJ. Prediction of diabetic foot ulcer occurrence using commonly available clinical information: the Seattle Diabetic Foot Study. Diabetes care. 2006;29(6):1202-7.
43. Williams BA, Geba D, Cordova JM, Shetty SS. A risk prediction model for heart failure hospitalization in type 2 diabetes mellitus. Clinical cardiology. 2020;43(3):275-83.
44. Li T-C, Wang H-C, Li C-I, Liu C-S, Lin W-Y, Lin C-H, et al. Establishment and validation of a prediction model for ischemic stroke risks in patients with type 2 diabetes. Diabetes research and clinical practice. 2018;138:220-8.
45. Mukamal K, Kizer J, Djoussé L, Ix J, Zieman S, Siscovick D, et al. Prediction and classification of cardiovascular disease risk in older adults with diabetes. Diabetologia. 2013;56(2):275-83.
46. Pfister R, Cairns R, Erdmann E, Schneider CA. A clinical risk score for heart failure in patients with type 2 diabetes and macrovascular disease: an analysis of the PROactive study. International journal of cardiology. 2013;162(2):112-6.
47. Reitzle L, Schmidt C, Du Y, Icks A, Hagen B, Ziese T, et al. Einschätzungen zur Prävalenz mikrovaskulärer Folgeerkrankungen bei Diabetes mellitus in Deutschland. Analyse von Versichertendaten aller gesetzlichen Krankenkassen für die Jahre 2012 und 2013. Bundesgesundheitsblatt-Gesundheitsforschung-Gesundheitsschutz. 2020;63(10):1219-30.
48. Basu S, Sussman JB, Berkowitz SA, Hayward RA, Yudkin JS. Development and validation of Risk Equations for Complications Of type 2 Diabetes (RECODe) using individual participant data from randomised trials. The Lancet Diabetes & Endocrinology. 2017;5(10):788-98.
49. Chow LS, Zmora R, Ma S, Seaquist ER, Schreiner PJ. Development of a model to predict 5-year risk of severe hypoglycemia in patients with type 2 diabetes. BMJ Open Diabetes Research and Care. 2018;6(1):e000527.
50. Dunkler D, Gao P, Lee SF, Heinze G, Clase CM, Tobe S, et al. Risk prediction for early CKD in type 2 diabetes. Clinical Journal of the American Society of Nephrology. 2015;10(8):1371-9.
51. McEwen LN, Karter AJ, Waitzfelder BE, Crosson JC, Marrero DG, Mangione CM, et al. Predictors of mortality over 8 years in type 2 diabetic patients: Translating Research Into Action for Diabetes (TRIAD). Diabetes care. 2012;35(6):1301-9.
52. SAS Institute Inc. SAS 9.4. Cary, NC.
53. RStudio Team. RStudio: Integrated Development Environment for R. Boston, MA: RStudio, PBC.; 2022.
54. Kuhn M, Wickham H. Tidymodels: a collection of packages for modeling and machine learning using tidyverse principles. 2020.
55. Python Software Foundation. Python programming language.
56. Paszke A, Gross S, Massa F, Lerer A, Bradbury J, Chanan G, et al. Pytorch: An imperative style, high-performance deep learning library. Advances in neural information processing systems. 2019;32.

57. Abadi Mı, Agarwal A, Barham P, Brevdo E, Chen Z, Citro C, et al. Tensor-Flow: Large-Scale Machine Learning on Heterogeneous Systems Software available from: https://www.tensorflow.org/2015 [
58. Falcon WA. Pytorch lightning. GitHub. 2019;3.
59. Joseph M. Pytorch tabular: A framework for deep learning with tabular data. arXiv preprint arXiv:210413638. 2021.
60. Joseph M, Sunil J, Borovec J, Fonnesbeck C, jxtrbtk, Andreas, et al. manujosephv/pytorch_tabular: v1.0.1. 2023.
61. Venables W, Ripley B. Modern Applied Statistics with S. Fourth edition. New York: Springer; 2002.
62. Greenwell B, Boehmke B. Variable Importance Plots—An Introduction to the vip Package. The R Journal. 2020;12(1):343–66.
63. Dubov E. Classifying Imbalanced Data Using Hellinger Distance 2019 [Available from: https://medium.com/@evgeni.dubov/classifying-imbalanced-data-using-hellinger-distance-f6a4330d6f9a.
64. Boulesteix AL, Janitza S, Kruppa J, König IR. Overview of random forest methodology and practical guidance with emphasis on computational biology and bioinformatics. Wiley Interdisciplinary Reviews: Data Mining and Knowledge Discovery. 2012;2(6):493-507.
65. Chirikov VV, Shaya FT, Onukwugha E, Mullins CD, dosReis S, Howell CD. Tree-based claims algorithm for measuring pretreatment quality of care in Medicare disabled hepatitis C patients. Medical care. 2017;55(12):e104-e12.
66. Hagan MT, Demuth HB, Jesús OD. An introduction to the use of neural networks in control systems. International Journal of Robust and Nonlinear Control: IFAC-Affiliated Journal. 2002;12(11):959-85.
67. Steyerberg EW, Vickers AJ, Cook NR, Gerds T, Gonen M, Obuchowski N, et al. Assessing the performance of prediction models: a framework for some traditional and novel measures. Epidemiology (Cambridge, Mass). 2010;21(1):128.
68. Perkins NJ, Schisterman EF. The Youden Index and the optimal cut-point corrected for measurement error. Biometrical Journal: Journal of Mathematical Methods in Biosciences. 2005;47(4):428-41.
69. Lo-Ciganic W-H, Huang JL, Zhang HH, Weiss JC, Wu Y, Kwoh CK, et al. Evaluation of Machine-Learning Algorithms for Predicting Opioid Overdose Risk Among Medicare Beneficiaries With Opioid Prescriptions. JAMA Network Open. 2019;2(3):e190968-e.
70. Ozenne B, Subtil F, Maucort-Boulch D. The precision–recall curve overcame the optimism of the receiver operating characteristic curve in rare diseases. Journal of clinical epidemiology. 2015;68(8):855-9.
71. Statistical Odds & Ends. 2020. Available from: https://statisticaloddsandends.wordpress.com/2020/06/07/what-is-the-delong-test-for-comparing-aucs/.
72. Robin X, Turck N, Hainard A, Tiberti N, Lisacek F, Sanchez J-C, et al. pROC: an open-source package for R and S+ to analyze and compare ROC curves. BMC bioinformatics. 2011;12(1):1-8.
73. Tran-The TD. Towards Data Science [Internet]2022. Available from: https://towardsdatascience.com/the-wrong-and-right-way-to-approximate-area-under-precision-recall-curve-auprc-8fd9ca409064.
74. Huang Y, Li W, Macheret F, Gabriel RA, Ohno-Machado L. A tutorial on calibration measurements and calibration models for clinical prediction models. Journal of the American Medical Informatics Association. 2020;27(4):621-33.
75. Van Calster B, McLernon DJ, van Smeden M, Wynants L, Steyerberg EW, Bossuyt P, et al. Calibration: the Achilles heel of predictive analytics. BMC Medicine. 2019;17(1):230.

76. Sedighi I. Interpretation of diagnostic tests: likelihood ratio vs. predictive value. Iranian Journal of Pediatrics. 2013;23(6):717-.
77. Romero-Brufau S, Huddleston JM, Escobar GJ, Liebow M. Why the C-statistic is not informative to evaluate early warning scores and what metrics to use. Critical Care. 2015;19(1):1-6.
78. Austin PC, Steyerberg EW. Events per variable (EPV) and the relative performance of different strategies for estimating the out-of-sample validity of logistic regression models. Statistical methods in medical research. 2017;26(2):796-808.
79. Bradter U, Altringham JD, Kunin WE, Thom TJ, O'Connell J, Benton TG. Variable ranking and selection with random forest for unbalanced data. Environmental Data Science. 2022;1:e30.
